# Enhanced Weight Management Program for Veterans With Posttraumatic Stress Disorder: A Randomized Clinical Trial

**DOI:** 10.1001/jamanetworkopen.2026.1904

**Published:** 2026-03-27

**Authors:** Katherine D. Hoerster, Nadiyah Sulayman, Rachel Hunter-Merrill, Scott Coggeshall, Lamont Tanksley, Moriah Brier, Lucas Donovan, Tiffanie Fennell, Kristen Gray, Dakota H. Evans, Kristin Rosendahl, Brian E. Saelens, Tracy Simpson, Karin M. Nelson

**Affiliations:** 1 Mental Health Service, Veterans Affairs (VA) Puget Sound Healthcare System, Seattle Division, Seattle, Washington; 2 Research and Development, VA Puget Sound Healthcare System, Seattle Division, Seattle, Washington; 3 Department of Psychiatry and Behavioral Sciences, School of Medicine, University of Washington, Seattle; 4 Department of Health Systems and Population Health, School of Public Health, University of Washington, Seattle; 5 Anesthesiology Service, VA Puget Sound Healthcare System, Seattle, Washington; 6 Department of Medicine, School of Medicine, University of Washington, Seattle; 7 General Medicine Service, VA Puget Sound Healthcare System, Seattle, Washington; 8 Seattle Children’s Research Institute, Seattle, Washington; 9 Department of Pediatrics, School of Medicine, University of Washington, Seattle; 10 Center of Excellence in Substance Addiction Treatment and Education, VA Puget Sound Health Care System, Seattle, Washington

## Abstract

**Importance:**

Posttraumatic stress disorder (PTSD) increases the risk of obesity and is associated with less weight loss in the Veterans Health Administration’s (VHA’s) weight management program (MOVE!). MOVE!+UP was developed to address unique barriers to weight loss among veterans with PTSD and elevated body mass index.

**Objective:**

To examine whether weight change differed between MOVE!+UP and MOVE! participants.

**Design, Setting, and Participants:**

This randomized clinical trial was conducted from October 6, 2020, to February 28, 2024, at 1 VHA site. Six- and 12-month outcomes were collected by masked assessors. Participants were veterans with PTSD and a body mass index of 25 or greater who were engaged in PTSD care.

**Interventions:**

Participants were randomly assigned to MOVE!+UP or MOVE!. Both groups received 16 weekly sessions of group-based standard behavioral weight management counseling via video. MOVE!+UP included cognitive behavior therapy–based content (eg, coping, relationship support, and community engagement) and a community-based walk to facilitate exercise and exposure to feared experiences (a PTSD treatment target). MOVE!+UP offered 2 individualized dietitian sessions to enhance benefit.

**Main Outcomes and Measures:**

The prespecified primary outcome was weight change at 6 months measured on a scale provided by the study.

**Results:**

Among 174 randomized participants (mean [SD] age, 55 [13] years; 113 men [65%]; 85 participants randomized to MOVE!+UP and 89 randomized to MOVE!), unadjusted mean (SD) weight loss at 6 months among MOVE!+UP participants was 8.9 (13.7) lb (decreased from 224.0 [42.0] to 215.1 [38.1] lb compared with 7.8 [13.8] lb among MOVE! participants (decreased from 232.9 [48.4] to 225.2 [50.2] lb). Adjusted intervention minus control mean weight change was −1.5 lb (95% CI, −5.9 to 2.9; *P* = .50). Secondary outcomes (change in PTSD symptoms at 6 and 12 months and weight change at 12 months) did not significantly differ between groups. No participants were withdrawn due to adverse events.

**Conclusions and Relevance:**

In this randomized clinical trial, weight change did not differ between a tailored weight management program for veterans with PTSD (MOVE!+UP) and VHA’s standard weight management program (MOVE!). These results suggest that research must continue to address weight loss among those with PTSD.

**Trial Registration:**

ClinicalTrials.gov Identifier: NCT04563741

## Key Points

**Question:**

Does an enhanced behavioral weight management intervention tailored to address unique barriers to weight loss for veterans with a body mass index of 25 or higher and posttraumatic stress disorder (PTSD) improve weight loss more than a Veterans Health Administration program?

**Findings:**

In this randomized clinical trial of 174 participants, 6-month weight loss was modest and did not differ significantly between the study groups. The 6- and 12-month PTSD symptom improvements and 12-month weight loss also did not differ between the study groups.

**Meaning:**

This study’s results suggest that further research is needed to improve care for veterans with PTSD and an elevated body mass index.

## Teaser

This randomized clinical trial examines whether a weight management program designed to address barriers to weight loss unique to veterans with posttraumatic stress disorder (PTSD) was more effective than an established Veterans Health Administration weight management program among veterans with PTSD.

## Introduction

Posttraumatic stress disorder (PTSD) increases the risk of obesity.^1^ Despite comparable engagement to those without PTSD, veterans with PTSD lose less weight in the Veterans Health Administration’s (VHA’s) weight management program (MOVE!).^2^ PTSD symptoms (eg, insomnia and avoidance) may affect exercise and diet, impeding weight loss.^3,4,5,6,7,8,9,10,11,12,13,14,15,16^ To address these MOVE! weight loss disparities, we developed MOVE!+UP.^17^ MOVE!+UP supplements MOVE!’s weight loss education and support with cognitive behavior therapy (CBT) to address PTSD-specific barriers. We previously piloted and iteratively refined MOVE!+UP among 44 participants; the cohort who received the final version reported high satisfaction and meaningful reductions in weight loss (mean [SD], 14.0 [3.7] lb) and PTSD symptoms (mean [SD], −17.9 [12.2]).^17^ Given these preliminary proof-of-concept findings, this study tested MOVE!+UP’s effectiveness relative to MOVE! in a randomized clinical trial. We hypothesized that MOVE!+UP participants would experience significantly greater weight loss than MOVE! participants by 6 months (primary outcome).

## Methods

The Veterans Affairs (VA) Puget Sound Healthcare System institutional review board approved this study. Eligible and interested veterans provided verbal informed consent (we obtained a waiver of written documentation from the institutional review board). This report adheres to the 2025 Consolidated Standards of Reporting Trials (CONSORT) reporting guidelines for randomized clinical trials.

### Participants

From October 6, 2020, to February 28, 2024, we enrolled 179 veterans (Figure). The trial protocol is available in Supplement 1.^18^ Approximately every 6 to 8 months, we sent recruitment materials to a cohort (9 cohorts in total) of randomly selected veterans meeting several VHA electronic health record (EHR)–based criteria: VA Puget Sound patients receiving PTSD care but not currently doing MOVE! with a body mass index (BMI; calculated as weight in kilograms divided by the square of height in meters) of 25 or greater and no dementia diagnosis. Using EHR-based race and ethnicity, we oversampled those who had a race other than only White. We aimed to recruit 20% or more women but achieved this without targeted sampling. Potentially eligible patients were also identified from self or clinician referral. We then used manual EHR review to determine whether patients were receiving guideline-concordant PTSD treatment: 2 or more PTSD-focused psychotherapy visits in the past 3 months plus an upcoming psychotherapy visit and/or current prescription of guideline-recommended medication.^19^

**Figure.**
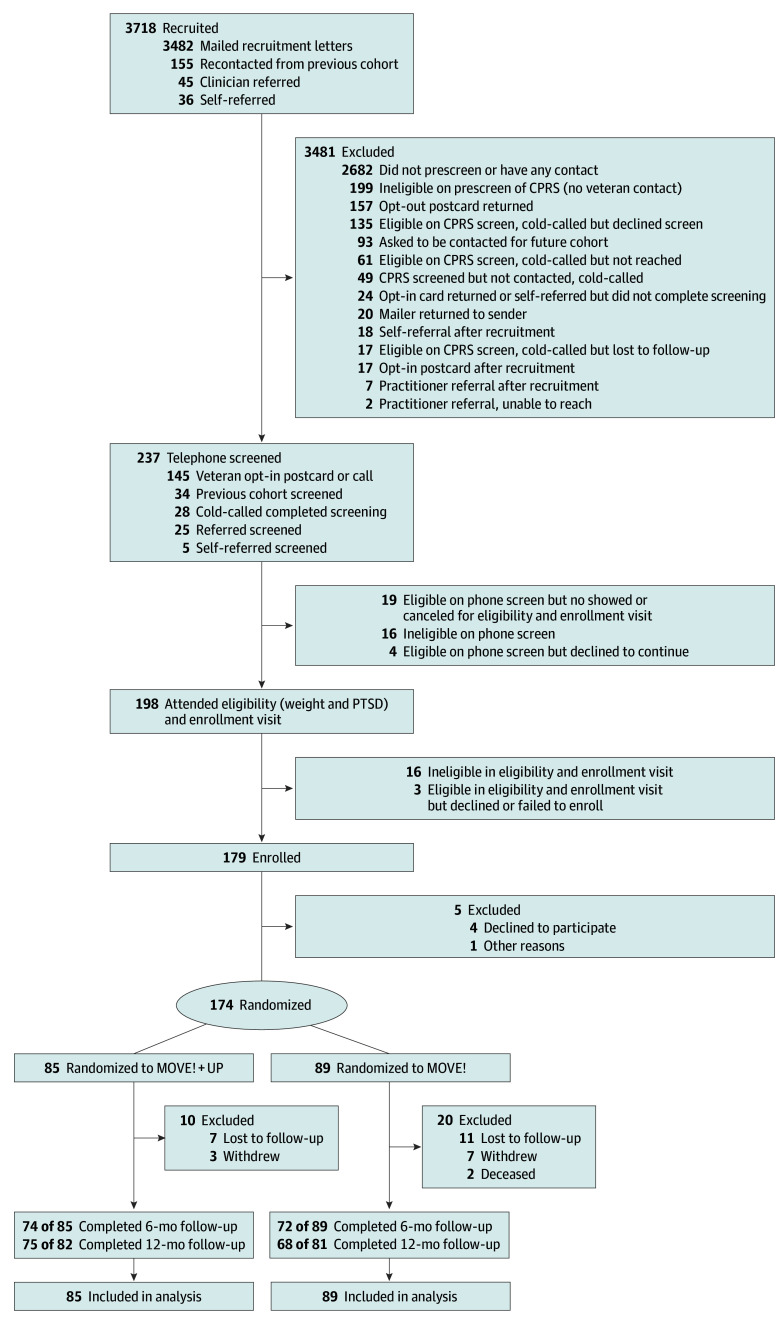
CONSORT Flow Diagram Comprehensive list of inclusion and exclusion criteria are presented in the trial protocol in Supplement 1. CONSORT indicates Consolidated Standards of Reporting Trials; CPRS, Computerized Patient Record System; PTSD, posttraumatic stress disorder.

We then phone screened participants for self-reported eligibility. Patients could self-report mental health treatment if they were only receiving non-VHA care at this stage. In addition, we confirmed self-reported weight and PTSD symptoms using the brief Primary Care PTSD Screen for *DSM-5* screener.^20^ To determine final eligibility, study staff conducted a video visit using VHA’s online platform VA Video Connect during which weight was measured on a scale we mailed to them (to confirm BMI ≥25 using height from the EHR). Veterans can join VA Video Connect visits from any video-enabled device (eg, smartphone); veterans without such a device were provided one free of charge from the VA. We confirmed the patient currently met PTSD screening criteria with the PTSD Checklist for the *DSM-5* (PCL-5).^21^

### Randomization and Masking

We randomized 174 of 179 enrolled participants to MOVE! (control) or MOVE!+UP (intervention) in a 1:1 ratio within each recruitment cohort (Figure), using a permuted block design, with varying block sizes (2 or 4). People performing data collection and analysis remained masked until after 12-month data collection.

### Control and Intervention Conditions

MOVE!+UP and MOVE! both provided 16 weekly sessions of group-based behavioral weight management content and support, including evidence-based education regarding physical activity and healthy eating, goal setting, and self-monitoring.^22^ MOVE!+UP and MOVE! were both delivered via VA Video Connect. Facilitators for both encouraged participants to send them weekly weight as well as food and activity tracking logs so facilitators could provide feedback and support accountability. Facilitators entered brief EHR notes for participants.

MOVE!+UP delivered not only all standard MOVE! content but also several tailored approaches.^18^ Although MOVE! sessions lasted 1 hour, MOVE!+UP sessions lasted 2 hours to allow for content targeting PTSD-related barriers and promoting PTSD recovery, including community engagement, CBT to address unhelpful thinking patterns, relationship support, and sleep hygiene. The 2-hour MOVE!+UP sessions also included a 30-minute neighborhood walk during each session to provide opportunities to get exercise while addressing hypervigilance-based barriers though exposure to feared activities (a common PTSD treatment target), after which participants discussed observations and learning. A psychologist and dietitian cofacilitated MOVE! (T.F. and K.R.), whereas a psychologist with PTSD training and a veteran peer support counselor cofacilitated MOVE!+UP (M.B. and L.T.). Because individualized dietician support can enhance outcomes, MOVE!+UP also provided 2 individual dietitian visits to address the previously observed outcome disparities.

### Measures

Participants completed a staff-administered survey and weight measurement at baseline, 6 months, and 12 months, self-completed a 16-week satisfaction survey, and provided accelerometer data at baseline and 6 months. We compensated participants up to $120 to encourage measurement completion ($20 for each measurement component).

We performed double data entry of 5% of baseline and follow-up surveys for randomized participants. Less than 1% of items had discrepancies, most attributable to open-text responses with slightly different wording but identical meanings. Discrepancies were reviewed and reentered. Four surveys with more than 3% discrepancies were reentered.

Baseline surveys assessed self-reported demographic characteristics (age, gender, race, ethnicity, educational attainment, employment status, and annual household income), experiences of weight-related stigma,^23^ use of assistive devices, and adaptive late-life function and disability.^24^ We determined baseline service–connected status (0%-100% for any condition) from the EHR. Race and ethnicity categories included Black, White, multiple races, and other race (including American Indian or Alaskan Native, Filipino, Korean, multiple categories, other Asian, other Pacific Islander, and some other race). We collected race and ethnicity data so that we could characterize our sample and how representative it was of the VA patient population and because race is associated with weight loss.^2^

#### Primary Outcome

The primary outcome was weight change at 6 months (follow-up minus baseline such that a negative number indicates weight loss and a positive number indicates weight gain). We selected 6- instead of 12-month weight change as the primary outcome because it aligns with how MOVE! assesses weight outcomes. Likewise, we present weight data in pounds for its comparability with VA administrative data. We measured weights on scales provided by the study, with weight visualized by study staff over video. If technology was a barrier, veterans could securely send a photograph of their weight on the study scale or provide self-reported weight. We documented the collection method, classifying it into 9 categories (eTable 1 in Supplement 2). For a sensitivity analysis, we dichotomized categories into those collected per protocol vs other.

We assessed weights for implausible or erroneous values using prespecified criteria.^25^ Using these criteria, only one weight was identified as being likely implausible (31% lost from baseline weight, without medical explanation). It was judged by 2 coinvestigators (B.E.S. and K.M.N.) masked to treatment assignment to be a data entry error and excluded.

#### Secondary and Exploratory Outcomes

We prespecified 6-month PTSD symptom change (follow-up minus baseline, measured with the PCL-5^21^) and 12-month weight change and PTSD symptom change as secondary outcomes. We prespecified several exploratory outcomes, including self-reported depression (Patient Health Questionnaire 8),^26^ diet quality (Starting the Conversation),^27^ eating habits (adult PACE measure),^28^ emotional overeating (modified Emotional Overeating Questionnaire),^29^ night eating (modified Night Eating Questionnaire),^30^ binge eating (modified Patient Health Questionnaire eating disorder module; yes vs no),^31^ insomnia (Insomnia Severity Index),^32^ internalized weight stigma (Weight Bias Internalization Scale),^33^ and encouragement and discouragement for physical activity and healthy eating (measure modified from previously developed measures).^34,35^

We measured 7-day physical activity^36^ using a wrist-worn accelerometer (Actigraph wGT3X-BT; Ametris), with 1-minute epochs. We used the Choi wear validation algorithm^37^ and required 12 h/d (valid day) for 3 or more valid days of wear. Participants were asked to log when they wore the accelerometer. We found no discrepancies between logs and accelerometer data. Vector magnitude of the 3 axes were compared with existing wrist-worn cut points: sedentary (<2860 counts/min), light (2860-3941 counts/min), and moderate or vigorous (>3941 counts/min).^38^ From those standard cut points, we calculated 2 exploratory outcomes: average light, moderate, and vigorous physical activity and average moderate and vigorous physical activity minutes per valid day. We did not identify any outliers (ie, light, moderate, and vigorous physical activity >4 h/d and moderate and vigorous physical activity >3 h/d on average) from potential faulty data capture.

#### Additional Prespecified Descriptive Variables

We assessed the percentage losing 5% or more of baseline weight^39^ and 9 points or more on the PCL-5 from baseline, at 6 months, and at 12 months. We assessed participation in weight-related and mental health care via self-report and from the EHR using primary or secondary stop codes (ie, codes used by the VHA to designate clinic visit types) for MOVE!, nutrition and dietitian visits, and outpatient psychotherapy. From the EHR, we collected information on bariatric surgery (none underwent it) and weight management and psychiatric medication prescriptions during the study period. We tracked participation in MOVE!+UP and MOVE! activities, such as joining sessions, and for MOVE!+UP, such as completing the walking component. Reasons for session nonparticipation were tracked by manual EHR review. We assessed program satisfaction via self-report questionnaire on program completion at 16 weeks.

We collected information related to adverse events (AEs; eg, new diagnoses, changes in health status, hospitalizations, and visits to emergency departments). We documented AEs reported to facilitators and study staff and at follow-up assessments. Two raters (K.D.H., L.D., and/or K.M.N.) assessed AE severity, relatedness, expectedness, and body system(s).

### Sample Size Calculation

We calculated the target sample size (N = 164; 82 per group) to detect a difference in weight change between MOVE!+UP and MOVE!, accounting for clustered randomization, using the inflation factor 1 + (μ − 1)ρ for cluster size m and intraclass correlation (ρ), and attrition.^40^ Based on prior weight loss trials and our pilot study, we expected MOVE!+UP participants to lose a mean of 12 lb, which corresponds to the minimal clinically important difference of 5% or more loss of baseline weight based on anticipated mean baseline weights.^39^ We expected those in MOVE! to lose a mean of 3.6 lb, consistent with MOVE! participants with PTSD, according to national VHA EHR data. Additional assumptions were made based on prior work: the outcome’s SD (13),^2,41^ the ICC (0.03),^42^ within-cohort, within-randomization treatment group cluster size of 8 veterans per randomized group, 90% power, a 2-sided α = .05, and 6-month attrition (25%).^43^ This sample size also provided 90% power (at α = .05) to detect a meaningful 9-point difference in PTSD symptom reduction^44^ between conditions at 6 months. We ultimately exceeded our randomization target by 10 participants to ensure sufficiently large treatment groups for the final cohort.

### Statistical Analysis

Along with our primary analyses, we conducted secondary, exploratory, fidelity, and sensitivity analyses. Analyses were conducted with R, version 4.4.1 (R Foundation of Statistical Computing). A 2-sided *P* < .05 was considered statistically significant.

#### Primary Analysis

We conducted an intention-to-treat analysis, whereby participants were analyzed as randomized regardless of intervention adherence, using a 2-sided α = .05 with no adjustment for multiplicity given the sole test.^45^ We used a 2-sided test to allow the evaluation of the possibility of an effect in both directions. We tested the primary hypothesis that MOVE!+UP would result in significantly greater weight loss than MOVE! using a linear mixed-effects regression model. The regression model accounted for clustering (random effect) among participants in the group-based MOVE!+UP and MOVE! treatments. To increase precision of outcome effect estimates,^46,47^ the regression also adjusted for baseline weight and an indicator of self-reported Black racial identity (Hispanic or non-Hispanic Black vs other), given Black individuals often lose less weight in weight management interventions due to social determinants of health (eg, inadequate access to healthful foods).^2,48^ This analysis approach was also used for the secondary 12-month weight analysis.

#### Prespecified Secondary and Exploratory Analyses

We used a similar model to weight outcome analyses but removed Black racial identity as a precision variable. Secondary and exploratory, hypothesis-generating outcomes were also afforded α levels of .05. For the dichotomous binge eating measure, we used a random intercept logistic regression model. For the 2 physical activity outcomes, we adjusted for number of valid days of accelerometer wear. Findings from secondary and exploratory analyses should be considered hypothesis-generating.

#### Missing Data

The numbers of patients with missing data in MOVE!+UP and MOVE! are given in Table 1, and descriptive data for those with and without missing 6-month data are given in eTable 2 in Supplement 2. We used race reported in the EHR for 3 participants who declined to answer. Otherwise, we addressed missing data in primary, secondary, and exploratory analyses using multiple imputation by chained equations (MICE), which can accommodate mixed data types and clustered data.^49,50^ MICE was performed using the outcome and baseline variables in the primary analytic model and prespecified auxiliary variables that are likely associated with missingness and/or the outcome based on a prior trial^25^ (gender, age, race, employment status, educational attainment, and medication use associated with substantial weight change). We created 50 imputed datasets, which were then analyzed and combined according to Rubin rules for multiple imputation.^51^

**Table 1.** Baseline Characteristics of the Study Sample

Characteristic	No. (%) of participants^a^		
	Overall (N = 174)	MOVE! (control) group (n = 89)	MOVE!+UP (intervention) group (n = 85)
Gender			
Men	113 (65)	57 (64)	56 (66)
Women	61 (35)	32 (36)	29 (34)
Age, mean (SD), y	55 (13)	55 (13)	54 (13)
Race			
Black or African American	31 (18)	12 (13)	19 (22)
White	107 (61)	58 (65)	49 (58)
Multiple categories	24 (14)	12 (13)	12 (14)
Other^b^	12 (7)	7 (8)	5 (6)
Any Black or African American race^c^	41 (24)	17 (19)	24 (28)
Hispanic Latino or Spanish origin	17 (10)	9 (10)	8 (9)
Employment status			
Employed full or part time	44 (25)	22 (25)	22 (26)
Disabled or receiving disability	64 (37)	34 (38)	30 (35)
Retired	23 (13)	11 (12)	12 (14)
Other (student, unemployed, or multiple)	41 (24)	21 (24)	20 (24)
Missing	2 (1)	1 (1)	1 (1)
Educational attainment			
Completed high school or less	6 (3)	5 (6)	1 (1.0)
Some college or vocational training	50 (29)	22 (25)	28 (33)
Completed associates degree	29 (17)	17 (19)	12 (14)
Completed college	50 (29)	28 (31)	22 (26)
Completed a graduate degree	37 (21)	16 (18)	21 (25)
Missing	2 (1)	1 (1)	1 (1)
Military branch			
US Air Force	20 (11)	11 (12)	9 (11)
US Army	81 (47)	41 (46)	40 (47)
US Coast Guard	3 (2)	1 (1)	2 (2)
US Marines	16 (9)	11 (12)	5 (6)
US Navy	44 (25)	21 (24)	23 (27)
Multiple categories	8 (5)	3 (3)	5 (6)
Missing	2 (1)	1 (1)	1 (1)
Relationship status			
Married or living with significant other	107 (61)	56 (63)	51 (60)
Other (divorced, never married, separated, widowed, or multiple)	65 (37)	32 (36)	33 (39)
Missing	2 (1)	1 (1)	1 (1)
Household annual family income, $			
≤40 000	36 (21)	18 (20)	18 (21)
40 001-60 000	34 (20)	20 (22)	14 (16)
60 001-80 000	26 (15)	12 (13)	14 (16)
80 001-100 000	30 (17)	16 (18)	14 (16)
>100 000	42 (24)	20 (22)	22 (26)
Missing	6 (3)	3 (3)	3 (4)
Service-connected status, %			
0	7 (4)	2 (2)	5 (6)
10-90	75 (43)	38 (43)	37 (44)
100	92 (53)	49 (55)	43 (51)
BMI, mean (SD)	34.3 (5.5)	34.7 (5.7)	33.9 (5.2)
Experienced weight stigma (range, 0 [best] to 3 [worst])			
Mean (SD)	1.4 (1.2)	1.4 (1.3)	1.4 (1.2)
Missing	2 (1)	1 (1)	1 (1)
Uses an assistive device			
Yes vs no	37 (22)	21 (24)	16 (20)
Missing	5 (3)	2 (2)	3 (4)
Adaptive late-life function and disability (range, 11 [worse] to 55 [better])			
Mean (SD)	34 (11)	33 (12)	36 (10)
Missing	6 (3)	4 (4)	2 (2)

Abbreviation: BMI, body mass index (calculated as weight in kilograms divided by the square of height in meters).

^a^ Unless otherwise indicated.

^b^ Racial categories included in the other response include American Indian or Alaskan Native, Filipino, Korean, multiple categories, Other Asian, Other Pacific Islander, and some other race. Although other options were provided, they were not selected by any participants. Categories were combined into other due to small cell sizes. eTable 2 in Supplement 2 gives the percentages that people selected for each racial category.

^c^ This variable includes anyone who noted they identified as Black or African American (eg, those who selected multiple categories), which is why there are higher numbers of Black or African American veterans in this variable than the race variable above, which has a multiple categories option.

#### Fidelity Analyses

We randomly selected 20% of recorded MOVE!+UP intervention sessions for fidelity review, selecting a range of cohorts and session numbers. Two raters (N.S. and D.H.E.) independently completed standard fidelity checklists developed for each session. Raters met to identify and resolve rating discrepancies. We also reviewed 6 recorded MOVE! sessions using comparable checklists.

#### Sensitivity Analyses

We performed several sensitivity analyses, including adjustment for weight management and mental health care before study enrollment, additional weight management treatment during the intervention period, and updating the MICE model to include additional variables associated with missingness (eTable 2 in Supplement 2): mean moderate and vigorous physical activity, PCL-5 score, and Weight Bias Internalization Scale score. Lastly, we examined how potential underreporting in follow-up weights that were not visualized^52^ could impact primary findings (eTable 7 in Supplement 2 presents sensitivity analysis methods and findings).

## Results

### Study Participants

A total of 174 participants (mean [SD] age, 55 [13] years; 113 men [65%] and 61 women [35%]; 31 [18%] Black, 107 [61%] White, 24 [14%] multiple races, and 12 [7%] other race) were randomized; 85 participants were randomized to MOVE!+UP and 89 were randomized to MOVE!. MOVE!+UP and MOVE! participants were similar, although baseline weight was slightly higher for MOVE! participants (full demographic details are presented in Table 1).

### Primary Outcome

Six-month weights were missing for 28 participants (11 in the intervention group and 17 in the control group) (Figure). At 6 months, MOVE!+UP participants lost an unadjusted mean (SD) of 8.9 (13.7) lb (mean [SD] baseline weight, 224.0 [42.0]; mean [SD] 6-month weight, 215.1 [38.1]), and MOVE! participants lost an unadjusted mean (SD) of 7.8 (13.8) lb (mean [SD] baseline weight, 232.9 [48.4] lb; mean [SD] 6-month weight, 225.2 [50.2] lb) (Table 2). In adjusted analyses (Table 3), there was no statistically significant difference between groups in weight change (mean adjusted between-group, intervention minus control, −1.52 lb; 95% CI, −5.93 to 2.89 lb; *P* = .50).

**Table 2.** Unadjusted Primary, Secondary, and Exploratory Outcomes

Outcome	Mean (SD)					
	MOVE! (control) group			MOVE!+UP (intervention) group		
	Baseline (n = 89)	Follow-up (n = 72)^a^	Difference (n = 72)^a^	Baseline (n = 85)	Follow-up (n = 74)^a^	Difference (n = 74)^a^
**Primary and secondary outcomes**						
Weight						
6 mo	232.9 (48.4)	225.2 (50.2)	−7.8 (13.8)	224.0 (42.0)	215.1 (38.1)	−8.9 (13.7)
12 mo^a^^,^^b^	235.1 (49.5)	227.1 (51.1)	−8.0 (16.4)	224.8 (41.8)	216.0 (39.5)	−8.8 (17.7)
**PTSD symptoms (range, 0 [best] to 80 [worst])**						
6 mo	54.2 (10.5)	46.6 (12.7)	−7.6 (9.9)	52.0 (9.8)	44.1 (13.2)	−7.9 (12.7)
12 mo^a^^,^^b^	53.9 (10.6)	44.2 (14.8)	−9.6 (12.9)	51.8 (9.8)	44.4 (13.4)	−7.4 (14.1)
**Exploratory outcomes^a^**						
Depression symptoms at 6 mo (range, 0 [best] to 24 [worst])^a^	14.4 (4.9)	12.5 (4.6)	−1.9 (4.6)	13.1 (4.0)	11.8 (5.2)	−1.4 (4.9)
Insomnia severity at 6 mo (range, 0 [best] to 28 [worst])	17.6 (5.6)	16.6 (6.8)	−0.9 (5.0)	16.6 (5.5)	15.4 (6.5)	−1.2 (6.0)
Diet quality at 6 mo (range, 0 [best] to 16 [worst])	7.5 (2.6)	6.0 (2.3)	−1.5 (2.7)	7.9 (2.5)	6.4 (2.6)	−1.5 (2.6)
Eating habits at 6 mo (range, 0 [best] to 4 [worst])	1.5 (0.7)	1.2 (0.7)	−0.3 (0.5)	1.6 (0.8)	1.2 (0.7)	−0.5 (0.6)
Emotional overeating at 6 mo (range, 0 [best] to 4 [worst])	1.3 (0.9)	0.9 (0.9)	−0.4 (0.9)	1.4 (0.9)	1.0 (0.9)	−0.5 (1.0)
Night eating at 6 mo (range, 0 [best] to 24 [worst])	7.2 (4.9)	5.5 (4.0)	−1.7 (3.8)	7.2 (4.6)	5.6 (4.1)	−1.6 (3.6)
Binge eating at 6 mo (yes vs no)	0.1 (0.3)	0.1 (0.2)	−0.1 (0.4)	0.2 (0.4)	0.1 (0.3)	−0.1 (0.4)
Light, moderate, and vigorous physical activity per day at 6 mo	238.1 (104.5)	230.3 (108.6)	−7.8 (76.0)	208.8 (88.6)	210.6 (89.2)	1.8 (82.8)
Moderate and vigorous physical activity per day at 6 mo	73.9 (63.0)	71.6 (57.3)	−2.3 (40.5)	61.9 (49.7)	61.9 (50.8)	0.0 (47.7)
Social support for healthy eating at 6 mo (range, 0 [worst] to 4 [best])	0.9 (0.6)	0.9 (0.6)	0.0 (0.7)	0.8 (0.7)	1.0 (0.6)	0.1 (0.6)
Discouragement for healthy eating at 6 mo (range, 0 [best] to 4 [worst])	0.6 (0.5)	0.6 (0.6)	0.0 (0.6)	0.6 (0.6)	0.5 (0.5)	−0.1 (0.4)
Social support for physical activity at 6 mo (range, 0 [worst] to 4 [best])	0.7 (0.7)	0.9 (0.7)	0.1 (0.6)	0.8 (0.7)	0.8 (0.6)	−0.1 (0.5)
Discouragement for physical activity at 6 mo (range, 0 [best] to 4 [worst])	0.6 (0.5)	0.5 (0.4)	−0.0 (0.5)	0.5 (0.6)	0.5 (0.5)	0.0 (0.5)
Weight bias internalization at 6 mo (range, 1 [best] to 7 [worst])	3.6 (1.2)	3.4 (1.5)	−0.2 (0.9)	3.5 (1.0)	3.3 (1.4)	−0.3 (1.1)

Abbreviation: PTSD, posttraumatic stress disorder.

^a^ Follow-up and difference sample sizes that differ from those presented are as follows: 12-month weight: n = 68 in MOVE! and 75 in MOVE!+UP; 12-month PTSD Checklist for the *DSM-*5 score: n = 67 in MOVE! and 74 in MOVE!+UP. The remaining sample sizes for baseline, follow-up, and change scores are presented in eTable 9 in Supplement 2.

^b^ Baseline values that were used in the 12-month unadjusted, complete case analyses differ from those used in the 6-month analyses because of differential follow-up samples across the 2 time points. The weight change numbers are different from the values for follow-up minus baseline because the denominator for the change statistics differs from the denominators for follow-up sample size minus baseline sample size due to missing data (missing data were accounted for in the primary analysis using multiple imputation). A positive number for the weight change statistic reflects weight gain. Weight loss is reflected when a negative number is presented. For example, the intervention arm lost an average of 8.9 lb at 6 months after baseline.

**Table 3.** Between-Group Differences and Adjusted Secondary and Exploratory Outcomes

Outcome	Difference (95% CI)		
	Unadjusted between-group difference^a^	Adjusted treatment effect	*P* value
**Secondary outcomes**			
Weight change, lb			
6 mo	−0.83 (−5.28 to 3.62)	−1.52 (−5.93 to 2.89)	.50
12 mo	−0.63 (−6.17 to 4.91)	−1.88 (−7.29 to 3.53)	.49
PCL-5 reduction			
6 mo	−0.17 (−3.84 to 3.5)	−0.65 (−4.22 to 2.92)	.72
12 mo	1.55 (−2.93 to 6.03)	0.85 (−3.46 to 5.15)	.70
**Exploratory outcomes at 6 mo**			
Depression symptoms	0.57 (−0.99 to 2.13)	0.01 (−1.46 to 1.47)	>.99
Insomnia Severity Index	−0.08 (−1.92 to 1.76)	−0.35 (−2.14 to 1.44)	.70
Diet quality	0.11 (−1.02 to 1.25)	0.17 (−0.81 to 1.15)	.73
Eating habits	−0.11 (−0.32 to 0.09)	−0.08 (−0.27 to 0.1)	.37
Night eating	0.19 (−1.03 to 1.42)	0.06 (−1.04 to 1.16)	.91
Emotional eating	−0.05 (−0.4 to 0.29)	−0.00 (−0.26 to 0.27)	.99
Binge eating prevalence	−0.02 (−0.12 to 0.08)	−0.02 (−0.12 to 0.07)	.65
Light, moderate and vigorous physical activity	6.07 (−23.53 to 35.67)	−1.81 (−29.39 to 25.76)	.90
Moderate and vigorous physical activity	0.48 (−15.31 to 16.28)	−1.73 (−16.44 to 12.99)	.82
Social support for healthy eating	0.09 (−0.11 to 0.29)	0.05 (−0.12 to 0.22)	.56
Discouragement for healthy eating	−0.12 (−0.28 to 0.05)	−0.11 (−0.25 to 0.04)	.15
Social support for physical activity	−0.15 (−0.36 to 0.05)	−0.14 (−0.32 to 0.05)	.16
Discouragement for physical activity	0.04 (−0.18 to 0.25)	0.02 (−0.13 to 0.17)	.80
Weight bias internalization	−0.06 (−0.41 to 0.3)	−0.07 (−0.43 to 0.28)	.69

Abbreviation: PCL-5, PTSD Checklist for the *DSM-5*.

^a^ Calculated as follows: ([intervention follow-up − intervention baseline] − [control follow-up − control baseline]).

### Secondary and Exploratory Outcomes

There were no statistically significant differences between MOVE!+UP and MOVE! for secondary or exploratory outcomes in unadjusted and adjusted analyses (Table 2 and Table 3). However, the primary regression analysis precision variable of Black race was significantly associated with 12-month weight gain in adjusted analyses (8.09; 95% CI, 1.67-14.52; *P* = .01).

### Descriptive Analyses

More than one-third of participants had clinically meaningful weight (≥5%) and PTSD symptom (≥9 points) reductions from baseline. Descriptively, a slightly greater proportion of MOVE!+UP participants had meaningful changes at 6 months, but at 12 months slightly more MOVE! participants did (eTable 3 in Supplement 2). On average, participants engaged in more than half of MOVE!+UP and MOVE! sessions, with comparable participation for both groups (Table 4). Satisfaction with MOVE!+UP and MOVE! was high, with mean (SD) ratings across all items of 4.3 (1.0) and 4.2 (1.0) of 5, respectively (eTables 4 and 5 in Supplement 2).

**Table 4.** Engagement in MOVE! and MOVE!+UP and Use of Other Care

Measure	Mean (SD)^a^	
	MOVE! (control) group (n = 89)	MOVE!+UP (intervention) group (n = 85)
**Weight management care**		
Intervention vs control group participation from baseline to 6 mo after baseline		
MOVE!+UP manual study intervention session attendance tracking^b^^,^^c^	NA	9.7 (5.5)
MOVE! manual study control group session tracking^b^	9.5 (5.6)	NA
Additional behavioral weight management care		
MOVE! sessions 12 mo before baseline according to EHR^d^	0.5 (1.4)	1.1 (4.5)
Nutrition or dietician visits 6 mo before baseline according to EHR^d^	0.2 (0.8)	0.4 (1.4)
Nutrition or dietician visits from baseline to 12 mo after baseline according EHR^d^	0.2 (1.0)	0.4 (1.4)
Non-VA weight loss care baseline to 6 mo after baseline according to self report, No. (%)	4 (4)	1 (1)
Non-VA weight loss care baseline to 12 mo after baseline according to self report, No. (%)	10 (11)	1 (1)
≥1 Weight loss medication according to EHR, No. (%)^e^		
12 mo before baseline	31 (35)	23 (27)
6 mo after baseline	32 (36)	24 (28)
12 mo after baseline	36 (40)	28 (33)
**Mental health care**		
Mental health visits		
12 mo before baseline according to EHR^d^	12.0 (16.0)	12.0 (14.0)
Baseline to 6 mo after baseline according to EHR^d^	4.0 (6.0)	16.0 (8.0)
Baseline to 12 mo after baseline according to EHR^d^	8.0 (13.0)	20.0 (13.0)
Non-VA from baseline to 6 mo after baseline according to self-report, No. (%)	14 (21)	17 (24)
Non-VA from baseline to 6 mo after baseline according to self-report, No. (%)	20 (29)	18 (24)
≥1 Psychiatric medication, No. (%)^f^		
12 mo before baseline according to EHR	49 (55)	49 (58)
Baseline to 6 mo after baseline according to EHR	52 (58)	49 (58)
Baseline to 12 mo after baseline according to EHR	53 (60)	53 (62)

Abbreviations: EHR, electronic health record; EVA, Veterans Affairs; NA, not applicable.

^a^ Unless otherwise indicated.

^b^ Reasons for nonparticipation in sessions included travel or vacation, illness or injury, technology issues, scheduling conflict (eg, another appointment), work related, weather, dropped by facilitators after inadequate participation, and no reason stated.

^c^ The mean number of visits among the 85 MOVE!+UP participants engaged in the walking component (mean [SD], 8.34 [5.35]).

^d^ MOVE! (stop codes 372 and 373), nutrition and dietician visits (stop codes 123, 124, and 175), and outpatient psychotherapy (stop codes 509, 510, 527, 533, 534, 539, 550, 552, 564, 565, 567, 568, 571, 573, 574, 575, and 719).

^e^ Dulaglutide, empagliflozin, fluvoxamine, furosemide, lamotrigine, liraglutide, losartan, phentermine, semaglutide, or topiramate.

^f^ Sertraline, paroxetine, fluoxetine, venlafaxine, prazosin, nefazodone, imipramine, or phenelzine.

### Fidelity

The percentage agreement between raters at first review was high (97.5% in the MOVE!+UP group and 95.3% in the MOVE! group). Fidelity across all sessions was 90% for MOVE!+UP and 75.7% for MOVE!.

### Sensitivity Analyses

Results of sensitivity analyses were consistent with the primary analysis (eTable 6 and eFigure 1 in Supplement 2). Results from the weight visualization sensitivity analysis suggested findings were robust to potential overreporting or underreporting bias among weight measurements not visualized by study staff, irrespective of the magnitude of bias explored (eTable 7 and eFigure 2 in Supplement 2).

### Adverse Events

There were 10 serious AEs (SAEs) (5 in the MOVE!+UP group and 5 in the MOVE! group) (eTable 8 in Supplement 2). Two MOVE! participants died. One death occurred before follow-up and was deemed unrelated. The other death occurred during 12-month follow-up. We had no information regarding the cause of the latter death, so we could not assign a body system or rate relatedness or expectedness. The remaining 8 SAEs involved hospitalizations and were deemed unrelated. The most common SAE body system was gastrointestinal (40%) for MOVE!+UP and cardiovascular (75%) for MOVE! participants. Among 268 non-SAEs (140 in the intervention group and 128 in the control group), the 3 most common systems for MOVE!+UP and MOVE! participants, respectively, were musculoskeletal (26.4% and 34.4%), psychological (22.1% and 14.1%), and respiratory (13.6% and 14.8%). No randomized participants withdrew due to AEs.

## Discussion

Among VHA patients with a BMI of 25 or greater and PTSD, a behavioral weight management intervention tailored to address unique needs of veterans with PTSD did not produce greater weight loss than VHA’s MOVE! program. Findings suggest that implementing MOVE!+UP in the VHA would not improve outcomes for veterans with PTSD compared with the VHA’s existing weight management program. Although MOVE!+UP included substantially greater investment (eg, longer sessions, a walking component, and dietician visits), both MOVE!+UP and MOVE! participants experienced modest 6-month weight loss, suggesting the enhancements did not adequately target weight loss. Prior national VHA EHR data that we used for power calculations indicated that MOVE! participants with PTSD lost a mean of 3.6 lb, yet the current study’s MOVE! group lost a mean of 7.9 lb. The relatively higher (yet still modest) weight loss we observed for MOVE! participants in the current study may, in part, be due to higher MOVE! engagement (in our study, participants completed a mean of approximately 10 sessions, higher than typical).^22^ However, even in a prior study^2^ of veterans with intensive MOVE! engagement (≥8 sessions), those with PTSD lost only a mean (SD) of 5.8 (13.0) lb at 6 months and only 23.7% lost 5% or more of their baseline weight, as opposed to the slightly higher 35% in MOVE! and 41% in MOVE!+UP in the current study. Importantly, weight loss seen at 6 months seems to be largely maintained through 12 months in both groups, a testament to the value of MOVE!. The difference between these national figures and the current study may be due to the substantial variability in MOVE! delivery throughout VHA, which affects participation and outcomes.^53,54^ It may be that if MOVE! is delivered by highly experienced staff with adequate fidelity to motivated participants willing to enroll in a year-long trial—as in our study—a substantial number of veterans with PTSD can achieve meaningful weight loss. It is therefore important to continue efforts to ensure consistent, high-quality delivery of MOVE! throughout the VHA, adequate resources (eg, supporting cofacilitation), and interventions to encourage motivation. Given modest weight loss in VA’s behavioral weight management programs,^22^ more robust interventions (eg, weight management medication) are needed to yield population-wide benefits.

As seen previously,^2,48^ Black veterans lost significantly less weight than veterans of races other than Black at 12 months. There are numerous reasons for such disparities, driven by social determinants of heath.^48^ Recent research found that Black veterans want more representative MOVE! facilitators, more weight- and culture-inclusive content, and content to address stress and mental health.^55^ Such changes should be considered, and further research is needed to reduce such disparities.

### Limitations

This study has several limitations. Our sample size was smaller than some trials because we designed it so that a statistically significant difference between groups would reflect a clinically meaningful difference, requiring a somewhat smaller sample size than some trials (especially those powered to detect a minimal difference between groups). Prompted by the COVID-19 pandemic and before launching the trial, we modified our procedures from previously planned in-person assessments to hold visits via video, which at times impeded visualization of participants’ weights. Although it is unclear how this might have impacted results, sensitivity analyses suggest this did not impact the validity of findings. In addition, due to the COVID-19 pandemic, we modified our intervention (and control groups) to be delivered by video, which may have affected their effectiveness; however, prior work has shown that telehealth-delivered interventions are just as efficacious while improving access.^56^ We used wrist-worn accelerometers to promote ease and adherence, which can overestimate activity^38^—less concerning because we were comparing within-person change. We used substantial recruitment efforts to achieve our target sample, thereby suggesting participants might not be broadly representative of veterans and that this level of outreach would be difficult to scale. Lastly, findings may not generalize to non-VHA populations given that veterans are provided many resources unavailable to most (eg, transportation to care and free MOVE! care). Furthermore, the PTSD experienced by veterans may be different than that experienced by the general population, which might further hinder generalizability of findings to the general population.

## Conclusions

This randomized clinical trial found that a behavioral weight management program tailored to address unique weight loss barriers for veterans with PTSD (MOVE!+UP) was not superior to MOVE! at promoting weight loss. Further research is needed regarding how to improve weight loss outcomes among veterans with a BMI of 25 or greater who have PTSD.

## Notes

Supplement 1. Trial Protocol

Supplement 2. **eTable 1.** Quality of weights taken during study visits **eTable 2.** Characteristics at baseline, stratified by missingness on primary outcome (N=174) **eTable 3.** Clinically meaningful change in weight and PTSD symptoms **eTable 4.** Satisfaction with MOVE! **eTable 5.** Satisfaction with MOVE!+UP **eTable 6.** Sensitivity analyses **eTable 7.** Weight visualization sensitivity analysis **eTable 8.** Body systems involved in adverse events through 12 months **eTable 9.** Number of participants in baseline and follow-up for exploratory measures **eFigure 1.** Sensitivity analyses **eFigure 2.** Weight visualization sensitivity analyses

Supplement 3.
Data Sharing Statement
