# Supplementary material for: Enhanced Weight Management Program for Veterans With Posttraumatic Stress Disorder: A Randomized Clinical Trial
Source: JAMA Netw Open. 2026 Mar 27;9(3):e261904. doi: 10.1001/jamanetworkopen.2026.1904 (PMC13032152; doi:10.1001/jamanetworkopen.2026.1904)
Supplement: Supplement 1. — Trial Protocol [file jamanetwopen-e261904-s001.pdf]

Human Subjects Protocol

VA Puget Sound IRB

MOVE!+UP: Testing a Tailored Weight Management Program for Veterans with PTSD

MIRB # 01865

Funding Agency: VA HSR&D

Principal Investigator: Katherine D. Hoerster

Version 23, 6/17/2025

Abstract

1. Objective(s) and Hypotheses:
Post-traumatic stress disorder (PTSD), prevalent among Veterans, puts Veterans at increased risk for obesity and related conditions. Veterans with PTSD lose less weight in VA's MOVE! weight management program, due to PTSD symptoms that interfere with activity and healthy diet. In addition, many Veterans who receive evidence-based PTSD treatment remain symptomatic. A behavioral weight loss program that augments standard PTSD care and targets PTSD-related weight loss barriers called MOVE!+UP was piloted and iteratively refined among 44 overweight Veterans with PTSD. MOVE!+UP is led by a psychologist and a Veteran peer support counselor, who provide complementary expertise. It includes 16 group sessions with 90 minutes of general weight loss support, coupled with Cognitive Behavior Therapy skills to address PTSD-specific barriers. Each session also includes a 30-minute community walk to address hypervigilance-based activity barriers and enhance classroom-based learning. Veterans receive two individual dietician visits, and counseling calls as needed. The cohort receiving the final *MOVE!*+UP package during the pilot reported high satisfaction and clinically meaningful weight loss and PTSD symptom reduction outcomes. Treatment targets like eating behaviors and insomnia also improved. *MOVE!*+UP effectiveness must be tested in a randomized trial.

We sought to test MOVE!+UP by comparing it to VA's standard MOVE! program in a randomized controlled trial. We hypothesize that MOVE!+UP participants will experience significantly greater weight loss and PTSD symptom improvement than those in the control condition.

2. Research Design:
Using a hybrid type 1 trial framework, 179 overweight/obese Veterans with PTSD enrolled in PTSD care enrolled in the study and n=174 were randomized to either usual care enhanced with enrollment in *MOVE!* (control) or usual care enhanced with *MOVE!*+UP (intervention). This study is guided by three aims: 1) Test whether intervention participants have greater 6-month weight loss (primary outcome) and 6-month PTSD symptom reduction and 12-month weight loss and PTSD symptom reduction (secondary outcomes), relative to controls; 2) Assess whether compared to control, intervention participants have greater improvements on 6-month treatment targets: physical activity, eating behavior, insomnia, depression, and social support; 3) Estimate intervention and control condition costs and utilization, and identify MOVE!+UP implementation barriers and facilitators, to contextualize Aim 1 and inform future implementation.

3. Methodology
Veterans were randomly assigned to participate in *MOVE!*+UP or *MOVE!*. Participating in the study took a total of about one year. Assessment visits were held when participants first enrolled and 6 months after enrolling in the study. A

satisfaction questionnaire was mailed to all participants at 16 weeks and a final weight and PTSD symptom reduction check was be done one year after enrollment.

4. Findings/Progress to Date:
There are no findings to date.

5. Relevance to VA Mission
MOVE!+UP is timely and efficient, simultaneously addressing physical and mental health of a priority Veteran group. *MOVE!*+UP is positioned to address HSR&D priorities by promoting mental health and improving PTSD symptoms, access to care, and whole health. This study is aligned with HSR&D and ORD methodological priorities by using a hybrid type 1 trial. This study's implementation-related data will place effectiveness findings in context and facilitate rapid translation to the field if *MOVE!*+UP is effective. This study will also provide insights about ways that general *MOVE!* and PTSD care can be enhanced to improve reach and effectiveness.

List of Abbreviations

Provide a list of all abbreviations used in the protocol and their associated meanings.

ACOS: Associate Chief of Staff
BMI: Body Mass Index
CBT: Cognitive-Behavioral Therapy
CDA: Career Development Award
CFIR: Consolidated Framework for Implementation Research
COIN: Center of Innovation
COLA: Cost-of-Living Adjustment
CPT: Current Procedural Terminology
CRU: Clinical Research Unit
CSU: Clinical Services Unit
CVD: Cardiovascular Disease
D&I: Dissemination and Implementation
DoD: Department of Defense
DMAP: Data Management and Access Plan
DSM-5: Diagnostic and Statistical Manual of Mental Disorders, 5th Edition
DSMC: Data and Safety Monitoring Committee
HERC: Health Economics Resource Center
HSR&D: Health Services Research and Development
M: Mean
MCA: Managerial Cost Accounting
MHS: Mental Health Service
NCP: National Center for Health Promotion and Disease Prevention
NIH: National Institutes of Health
OMHSP: Office of Mental Health and Suicide Prevention Services
PA: Physical Activity
PBSCI: Department of Psychiatry and Behavioral Sciences
PCL-5: PTSD Checklist for DSM-5
PTSD: Posttraumatic Stress Disorder
QUERI: Quality Enhancement Research Initiative
RCT: Randomized Controlled Trial
RE-AIM: Reach, Effectiveness, Adoption, Implementation, and Maintenance Framework
REP: Replicating Effective Programs Framework for Health Care Interventions
SD: Standard Deviation
TMS: Talent Management System
UW: University of Washington
VA: Department of Veterans Affairs
VAPSHCS: VA Puget Sound Health Care System
VEB: Veteran Engagement Board
VVC: VA Video Connect
WL: Weight Loss

Contents

Protocol Title: 6
1.0 Key Study Personnel 6
2.0 Introduction 8
3.0 Objectives 12
4.0 Resources and Study Roles 12
5.0 Study Procedures 15
5.1 Study Design 15
5.2 Recruitment Methods 19
5.3 Informed Consent Procedures 23
5.4 Inclusion/Exclusion Criteria 24
5.5 Study Evaluations 25
5.6.a. Intervention Content and Delivery 33
5.6.b. Data Analysis 37
5.7 Withdrawal of Subjects 44
5.8 Union Notification 44
6.0 Reporting 45
7.0 Privacy and Confidentiality 47
8.0 Project Management and Communication Plan 48
8.1 Project Management 48
8.2 Communication Plan 49
9.0 Information Security and Data Storage/Movement 49
10.0 References 51

Protocol Title: *MOVE!*+UP: Testing a Tailored Weight Management Program for Veterans with PTSD

1.0 Key Study Personnel

- **VA Puget Sound Healthcare System**

Principal Investigator
Katherine Hoerster, PhD, MPH
HSR&D Core Investigator and Staff Psychologist
Mental Health Clinic
HSR&D COIN
VA Puget Sound Health Care System
Assistant Professor
Department of Psychiatry & Behavioral Sciences
University of Washington
Phone: 206-277-4203
Email: Katherine.Hoerster@va.gov

Co-Investigators:
Karin Nelson, MD, MSHS
HSR&D Core Investigator and Staff Physician
Primary Care and Women's Clinic
HSR&D COIN
VA Puget Sound Health Care System
Professor
Department of Medicine Division of General Internal Medicine
University of Washington
Email: Karin.Nelson@va.gov

Tracy Simpson, PhD
Investigator and Staff Psychologist
Center of Excellence in Substance Addiction Treatment and Education (CESATE)
VA Puget Sound Health Care System
Professor
Department of Psychiatry & Behavioral Sciences
University of Washington
Email: Tracy.Simpson@va.gov

Edwin Wong, PhD
HSR&D Investigator
HSR&D COIN
VA Puget Sound Health Care System
Research Assistant Professor

Department of Health Services
University of Washington
Email: Edwin.Wong@va.gov

Kristen E. Gray, PhD, MS
HSR&D Core Investigator
HSR&D COIN
VA Puget Sound Health Care System
Research Assistant Professor
Department of Health Services
University of Washington
Phone: 206-764-2056
Email: Kristen.Gray2@va.gov

Study Coordinator

Nadiyah Sulayman
VA Puget Sound Health Care System
Email: Nadiyah.Sulayman@va.gov

Collaborating Sites

Boise VA Health Care System
Site Principal Investigator

Moriah Brier, PhD
Boise VA Health Care System
Psychologist, V20 CRH TelePain
500 W Fort St, Boise, ID 83702
Phone: 206-277-1062
Email: Moriah.Brier@va.gov

- **Consultants**
  (At other institutions, not covered under the VA IRB approval; will not have access to study data or be involved in any study recruitment or data collection procedures.)

Brian Saelens, PhD
Professor
Department of Pediatrics
Department of Psychiatry & Behavioral Sciences
Adjunct Professor
School of Public Health, Department of Health Services
University of Washington
Researcher

Seattle's Children's Research Institute
Email: brian.saelens@seattlechildrens.org

2.0 Introduction

Posttraumatic stress disorder (PTSD), common among Veterans, adversely affects health in part due to inactivity and poor diet. PTSD is associated with increased risk for premature mortality[2] in part due to the high burden of overweight, and chronic diseases like cardiovascular disease (CVD) and diabetes.[1] VA administrative data indicate 83% of VA users with PTSD are overweight or obese, compared with 74% of the general VA patient population. Health outcomes are worse[2] and healthcare costs are higher[15] for Veterans with PTSD than Veterans with other or no mental health conditions. Being physically active and having a healthy diet can prevent and address CVD, diabetes, and obesity.[4] Unfortunately, as detailed in a systematic review co-authored by PI, Dr. Hoerster, PTSD is associated with unhealthy diet and inactivity.[21] Research led by Dr. Hoerster shows that Veterans with PTSD are more likely to have binge eating disorder than those without PTSD,[22] and other recent research shows that PTSD is associated with emotional[33,34] and night[35] eating, all of which can contribute to excess weight. It is critically important to better promote healthy lifestyles among Veterans with PTSD to address their high obesity, CVD, and diabetes burden.

Veterans with PTSD lose less weight in VA's weight management program, *MOVE!*. Implemented in VA in 2006,[17] *MOVE!* is an evidence-based program for obesity management.[36] The curriculum uses cognitive- behavioral techniques like goal setting and self-monitoring and applying autonomy-supporting counseling like motivational interviewing[37] mostly in in-person group sessions.[17] Nearly 20% of those who participated in at least two sessions lost ≥ 5% baseline weight at six months,[17] an amount associated with reduced CVD and diabetes risk.[18] Rates of clinically meaningful weight loss nearly double (31.6%) for the 14% of *MOVE!* patients with "intense and sustained" participation (≥8 sessions four-to-six months after enrollment).[17] While promising, Dr. Hoerster found that among those with intense and sustained participation, compared to those without mental health conditions, Veterans with PTSD were less likely to lose a clinically meaningful amount of weight (23.7% vs. 28.7% at 6 months; aOR=0.89; p=.008) and lost significantly less total weight at 6 and 12 months.[19]

PTSD symptoms may interfere with physical activity and healthy diet, impacting *MOVE!* outcomes. Dr. Hoerster and colleagues found Veterans with PTSD in *MOVE!* were significantly more likely to report barriers to weight loss than those without mental health conditions[20] and that they would benefit from strategies to manage mental health symptoms in *MOVE!*.[23] PTSD symptoms include behavioral avoidance, diminished interest in activities, negative beliefs and emotions, social isolation, and sleep disturbance,[14] all of which are potential barriers to physical activity and healthy eating behavior.[38-42] PTSD hyperarousal has been found to interfere with exercise.[26] Dr. Hoerster found that depression co-morbid with PTSD also affects healthy lifestyles.[43] Behavioral weight management could address disproportionate burden of obesity among Veterans with PTSD but barriers to physical activity and healthy diet likely undermine such interventions' effectiveness, suggesting tailoring is needed. Tailored behavioral weight management programs can be effective for those with other mental health conditions,[44,45] but there are no tailored programs for people with PTSD.

Many with PTSD remain symptomatic after evidence-based care. Incorporating PTSD treatment elements into weight loss programs could help Veterans with PTSD lose weight and improve PTSD. Treating PTSD is a top VA priority, with VA promoting delivery of evidence-based cognitive-behavioral therapy (CBT) such as Cognitive Processing Therapy and Prolonged Exposure.[11] These and other effective PTSD treatments[46] are based on CBT models that highlight the role of beliefs, and behavioral conditioning and learning in developing irrational fear structures that can be addressed through systematic exposure to feared stimuli (in vivo exposure), resulting in fear extinction, reduced unhelpful beliefs, and increased engagement in valued activities.[47] While Cognitive Processing Therapy and Prolonged Exposure have the strongest evidence for reducing PTSD symptom severity,[46] only one-third of Veterans who receive them reduce symptoms below the PTSD diagnostic criteria threshold.[12] VA also encourages select psychiatric medications if psychotherapy is not available or preferred,[11] but PTSD remission is even less common with those medications.[13] It is therefore important to offer various treatment options as part of mental health treatment for chronic PTSD.[14] Because physical activity improves PTSD,[7,9] including in an RCT on which Dr. Hoerster collaborated,[8] and a behavioral weight loss program in the general population reduced PTSD symptoms,[10] **behavioral weight management that addresses PTSD-related barriers with CBT may improve weight and PTSD, providing an adjunctive treatment to address limitations of weight management and PTSD care.**

Key Preliminary Study: MOVE!+UP, a tailored weight loss program for overweight Veterans with PTSD, may address MOVE!'s limitations while also improving PTSD symptoms among Veterans with PTSD. Funded by PI Dr. Hoerster's 5-year HSR&D Career Development Award (CDA), we built upon Dr. Hoerster's clinical and research expertise in PTSD and health behavior change to develop MOVE!+UP, which addresses the unique barriers to weight loss for Veterans with PTSD. We piloted MOVE!+UP with five cohorts (total N=44), modifying it between each cohort based on quantitative and qualitative data.[24,25] Initially, MOVE!+UP was led by a peer support counselor in recovery from mental illness, a model that can improve weight[48] and other health and psychosocial outcomes,[49,50] with strong VA support.[27,51] Originally, MOVE!+UP was designed to augment MOVE! with four in-person group and six brief phone counseling sessions. Cohorts 1-4 were satisfied, and had modest improvements in physical activity, diet quality and PTSD.[24,25] However, despite substantial efforts to promote MOVE! engagement to obtain general weight management support, few were willing to attend, contributing to modest weight loss (-1.8 pounds).[24,25] Many requested MOVE! be integrated with MOVE!+UP's highly-valued PTSD content. While participants appreciated the peer counselor's support, they requested health professional involvement to address specialized PTSD and weight loss needs.

Based on data from cohorts 1-4, we made substantial changes to MOVE!+UP, and re-piloted the final package with cohort 5 (n=8 Veterans), whose data are presented below and suggest **MOVE!+UP is acceptable and may improve weight and PTSD symptoms for Veterans with PTSD.** The final MOVE!+UP program is co-led by a psychologist with specialized PTSD training, and

Table 1. Cohort 5 16-Week Weight, PTSD, and Engagement

**Weight and PTSD outcomes**	Mean (SD) or %
Total weight lost, pounds*	13.9 (4.0)
% baseline weight lost	6.1 (2.1)
% lost ≥ 5% baseline weight	71%
PTSD symptom severity (range: 0-80)*	-17.9 (12.2)
**MOVE!+UP sessions attended**	
In-person group (16 total)	10.5 (4.4)
% attended ≥ 8 sessions	75%
% of MOVE!+UP walks attended	95%
Dietician visits (2 offered)	1.8 (0.5)
Telephone calls (as needed)	2.6 (1.7)

*p<.05 change from baseline to 16-week follow-up

a Veteran peer support counselor. It consists of 16 in-person group sessions that involve general MOVE! weight loss support (e.g., goal setting, nutrition guidance, activity/diet monitoring). To address PTSD-related barriers, MOVE!+UP also incorporates CBT elements such as addressing unhelpful thinking patterns, relationship support, and sleep hygiene. Each session includes a 30-minute community walk because activity increases retention in MOVE!,[52] and to address hypervigilance-based activity barriers[26] through experiential learning. Participants walk and talk together about how they are applying MOVE!+UP lessons and coping with anxiety from being active in public, with the goal of increasing community-based physical activity and engagement. Participants receive two individual dietician visits. As-needed counseling calls and coordination with medical, mental health, and/or social services are provided.

Table 1 presents 16-week weight, PTSD, and participation rates for those receiving MOVE!+UP's final version (pilot cohort 5). Compared to national VA data we published on Veterans with PTSD in MOVE!,[19] more MOVE!+UP participants achieved clinically meaningful weight loss (71% vs. 23.7% lost ≥ 5% baseline weight); findings were comparable at 6 months. Average improvement in PTSD exceeded the threshold for meaningful clinical improvement.[53] Reductions were large (Cohen's d=1.47), exceeding effect sizes found in trials of psychotherapy for PTSD (g = 1.12)[29] and VA effectiveness studies (d=0.87).[54] PTSD changes were comparable at 6 months. Participation was high, with more MOVE!+UP participants engaging in at least 8 sessions than Veterans with PTSD in MOVE! (75% vs. 14%).[19] Table 2 presents cohort 5's 16-week changes on *treatment targets*. Eating behavior improved on numerous indicators, and while not significant, the proportion with binge eating disorder decreased (25% vs. 0%). Although weekly physical activity did not increase significantly, the proportion meeting recommendations[30] increased by 29%. Average improvement in insomnia met the threshold for meaningful clinical improvement.[55] While depression severity did not decrease significantly, the proportion meeting depression screening criteria decreased by 40%. Participants reported significant improvements on social support for diet and activity.

Across 30 items with a 1-5 Likert scale, average satisfaction was M=4.3 (SD=0.3). Qualitative interviews reinforced Veteran satisfaction with all aspects of MOVE!+UP (Table 3).

Table 2. Cohort 5 16-week *Treatment Targets*

Measures	Mean Diff (SD)
Diet quality (range: 0-16; lower=better)*	-2.9 (3.1)
Night eating (range 0-24)*	-1.9 (1.9)
Emotional eating (range 0-4)*	-0.54 (0.4)
Weekly physical activity minutes	290.0 (611.2)
Insomnia severity (range: 0-28)*	-7.7 (4.2)
Depression severity (range: 0-24)	-5.0 (7.2)

*p≤.05 change from baseline to 16-week follow-up

Table 3. Qualitative Feedback from Cohort 5 Participants: Characteristic Quotes

Overall	"If I was going to sum [MOVE!+UP] up into one phrase, it would be: a program designed to help an individual to help themselves to a better, healthy life and lifestyle. I liked all of it." [MOVE!+UP helps you] "get some balance in your life, learn how to eat right, learn some nutrition, learn how to get out, learn some social skills..to better your whole life."
Behavior	"My..eating..changed, my physical activity..changed…now it has become a habit..."
PTSD	"It opened up my eyes to how..and the amount of food I was eating. Basically, if I was getting..depressed or into my thoughts, or being alone or with the PTSD…I'd eat more." "[It helped me] do things that are more relaxing for me to do, and get me out of my shell..and interact with other people that are suffering from PTSD too." "[MOVE!+UP] made me want to leave the house...when I'm doing physical activity, I don't

	have to focus on the PTSD or the negative." "I kind of did some healing with the PTSD. This has been a 10-year journey for me and I'm just now kind of starting my life."
Maintenance	"I want to keep going. I don't want to plateau…I want to keep going down."
Facilitators	"Besides just the support, knowledge and positive feedback that they gave us, they were just people that I trusted, which says a lot for someone with PTSD." "I liked..that [peer counselor] had..been in the service and..knows..the things that go on."
Walk	"We'd talk about what was going on at home and how we were trying to follow the program. We formed a little camaraderie."
Compared to MOVE!	"I think this one is better…The other MOVE!, they didn't ask you what your symptoms were. [MOVE!+UP] is geared specifically for people with PTSD."

Summary. Despite VA efforts to promote access to evidence-based care for obesity and PTSD,[11,32] many Veterans with PTSD do not benefit sufficiently from those services. We found that weight loss in *MOVE!* is suboptimal for Veterans with PTSD,[19] likely due to activity and healthy eating barriers.[20] PTSD often remains symptomatic after completing evidence-based treatment, necessitating adjunctive treatments.[12] Our pilot results showed *MOVE!*+UP is feasible and valued; and pilot cohort 5 had greater weight loss than Veterans with PTSD in *MOVE!*,[19] and meaningful improvements on PTSD and treatment targets. We propose to compare overweight Veterans with PTSD enrolled in PTSD treatment assigned to usual care enhanced with enrollment in *MOVE!* (control) vs. usual care enhanced with *MOVE!*+UP (intervention) in a two-site hybrid type 1 trial.[31] Figure 1 illustrates how we expect *MOVE!*+UP to address PTSD-specific weight loss barriers to improve health behaviors and mental health indicators, which synergistically affect each other, to reduce weight and PTSD symptoms.

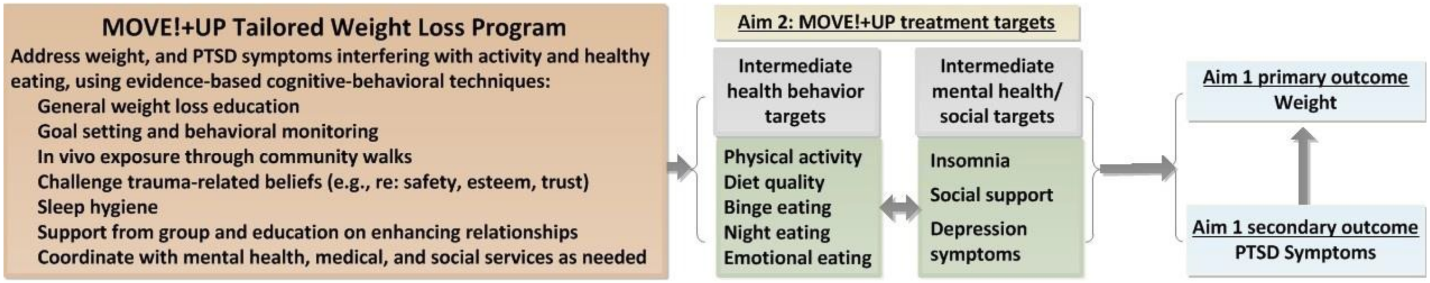

Figure 1. MOVE!+UP Conceptual Model and RCT Aims

3.0 Objectives

Posttraumatic Stress Disorder (PTSD) is common among Veterans and results in poor psychological functioning, quality of life, and physical health. This includes having disproportionately high rates of obesity, in part due to PTSD symptoms interfering with physical activity and healthy diet. Unfortunately, Veterans with PTSD have poorer weight loss outcomes than those without PTSD in VA's existing weight management program, *MOVE!*. Based on pilot work, it appears that MOVE!+UP, a weight management program that augments standard PTSD care and targets PTSD-related barriers to weight loss, improves weight and PTSD symptoms. Whether it improves these issues more than standard VA care is in need of study, which is the focus of the proposed research. The proposed study also seeks to understand factors that would interfere with and facilitate implementing the program in VA if it is effective.

We expected to enroll approximately 164 overweight or obese Veterans with PTSD who are currently receiving PTSD care at VA Puget Sound into this hybrid type 1 randomized controlled trial. We enrolled an additional 15 participants (a total of 179) to ensure adequate participants in the final cohort to have robust groups and to ensure 164 Veterans were randomized, per primary analysis power calculations. Participating in the study took a total of a year. Assessment visits were held when participants first enroll and 6 months after enrolling in the study. A final weight and PTSD symptomology assessment was done one year after enrollment. A questionnaire was also mailed to all participants at 16 weeks to assess satisfaction with the weight management program they participated in. The study aims were as follows:
Aim 1: Among overweight or obese Veterans with PTSD enrolled in PTSD care, test whether intervention participants have greater 6-month weight loss (primary outcome), and 6-month PTSD symptom reduction and 12-month weight loss and PTSD symptom reduction (secondary outcomes), relative to controls.

Aim 2: Assess whether compared to control, intervention participants have greater improvements on 6-month treatment targets: physical activity, eating behavior, insomnia, depression, and social support.

Aim 3: Estimate intervention and control condition costs and utilization, and identify MOVE!+UP implementation barriers and facilitators, to contextualize Aim 1 and inform future implementation.

4.0 Resources and Study Roles

<u>VA Puget Sound Healthcare System</u>
Katherine Hoerster, PhD, MPH (Principal Investigator)
Dr. Hoerster is a VA Puget Sound Health Care System (VAPSHCS) HSR&D Core Investigator and Staff Psychologist, and Associate Professor in the University of Washington (UW) Department of Psychiatry and Behavioral Sciences (PBSCI). She is responsible for the overall conduct of the study, including scientific design, methods, and measurement procedures. She oversees staff hiring and training, subject recruitment and retention, intervention implementation, data analysis and interpretation, manuscript preparation, and reporting of final results. She provided clinical supervision to the MOVE!+UP facilitators. Dr. Hoerster's salary was covered by her HSR&D career development award (CDA) through the end of the CDA award (May 2020) and then by VA Puget Sound mental health service (MHS) as she is a permanent staff psychologist.

Karin Nelson, MD, MSHS (Co-Investigator)
Dr. Nelson is a VAPSHCS HSR&D Core Investigator and staff physician in primary care and women's clinic, and Professor in the UW Department of Medicine Division of General Internal Medicine. She directs the Primary Care Analytics Team for the Office of Primary Care for the Veterans Health Administration. She is a well-funded investigator. She and the PI have collaborated closely on research projects since 2009, and she was primary mentor on Dr. Hoerster's 5-year HSR&D CDA focused on developing MOVE!+UP. She is applying her extensive expertise in chronic disease management, peer support interventions, and conducting large VA behavioral interventions to the current project.

Tracy Simpson, PhD (Co-Investigator)
Dr. Simpson is a VAPSHCS Psychologist and Investigator in the Center for Substance Abuse Treatment and Education, and Professor at the UW Department of PBSCI. She has been funded extensively by NIH and VA. She is an expert on PTSD treatment, with a specific emphasis on treatments that address substance use disorders in this population, expertise she applies to the current project. She and Dr. Hoerster have collaborated on several projects since 2010, and she served as a Co-mentor on Dr. Hoerster's HSR&D CDA.

Edwin Wong, PhD (Co-Investigator)
Dr. Wong will oversee cost-related data analyses that will be conducted if the trial results demonstrate a significant effect (this is the only appropriate context for a cost analysis). Dr. Wong is a Research Associate Professor in the Department of Health Services at the UW and an Investigator at the VAPSHCS HSR&D COIN. Dr. Wong is a health economist and health services researcher whose research, in part, examines the economic implications of interventions delivered at the health system level. This includes experience measuring the costs and population cost-effectiveness of enhanced implementation of evidence-based, trauma-focused psychotherapy for treatment of post-traumatic stress disorder in the VA. He also has extensive expertise in modeling costs and other health services related outcomes using a wide array of econometric methods. Dr. Wong will lead the economic analyses proposed in this study if relevant, including overseeing data acquisition, the development of analytic plans, and supervising the analysis of cost and utilization data by the study analyst, as well as preparing dissemination products for economic analyses.

Kristen Gray, PhD (Co-Investigator)
Dr. Gray is a VAPSHCS HSR&D Core Investigator and a Research Assistant Professor in the UW School of Public Health Department of Health Systems and Population Health. Dr. Gray is a PhD-trained epidemiologist with content expertise in social support, chronic disease self-management, and health promotion and disease prevention, as well as methodological expertise in study design and data analysis for observational and interventional studies. Dr. Gray has collaborated closely with Dr. Hoerster on several projects, including as MPI with Dr. Hoerster of the ongoing HSR&D-funded trial of a virtual peer health coaching intervention for Veterans with multiple chronic health and mental health conditions (HSR&D IIR 21-100) and on an ongoing evaluation of MOVE in partnership with the VA NCP. Dr. Gray will provide guidance on data analysis for the trial and will assist with interpretation of results.

Nadiyah Sulayman, BA (Research Coordinator)
Ms. Sulayman is a Research Health Science Specialist at the VAPSHCS HSR&D COIN who has been with VA for over five years. She has coordinated multiple studies, including trials and qualitative research. She was the project coordinator for Dr. Hoerster's MOVE!+UP pilot, overseeing all procedures, including performing data entry and data quality control procedures, preparing and maintaining IRB and other study materials, conducting assessments and qualitative interviews, and performing qualitative analyses under the guidance of Dr. Hoerster. Ms. Sulayman oversaw all recruitment, assessment, retention, data monitoring, and data quality control activities for the proposed project.

Vyshnika Sriskantharajah, BS (Data Manager)
Mrs. Sriskantharajah is a VAPSHCS HSR&D COIN data manager. She has a BS in Biotechnology from the University of Houston and a graduate certificate in Biomedical

Informatics from the University of Texas Health Science Center School of Biomedical Informatics. She has prior experience with REDCap, SQL, R, and Python from her previous work experience. She built the study's databases.

Lamont Tanksley, MA
Mr. Tanksley wasis a MOVE!+UP co-facilitator. Mr. Tanksley co-led MOVE!+UP with Dr. Brier. Mr. Tanksley is the peer support counselor who delivered MOVE!+UP during the pilot. He is paid by MHS and had time protected for delivering MOVE!+UP, CPRS documentation, intervention follow-up activities (e.g., care coordination), and ongoing training and supervision.

Ashley Paschke, RD
Ms. Paschke delivered the 2 dietician visits that are part of MOVE!+UP. She received training in addressing weight among Veterans with PTSD. Ms. Paschke worked with the most recent MOVE!+UP pilot.

Dakota Houseknecht (Research Assistant)
Mx. Houseknecht completes MOVE!+UP administrative tasks and other assessment-related activities.

Eric Epler, BS (Research Coordinator)
Mr. Epler completes MOVE!+UP administrative tasks and other assessment-related activities.

Lucas Donovan, MD, MS (Study DSMC Member/AE Rater)
Dr. Donovan is a VA board certified physician and staff member of the HRS&D service line in Seattle. He serves as one of the DSMC members in evaluation of study data and report on patient safety issues/concerns. He is involved in determining AEs (independently) throughout the study.

Collaborating Site, Boise VA Health Care System
Moriah Brier, PhD, Site Principal Investigator
Dr. Brier co-led MOVE!+UP. She is affiliated with the CRH and Boise VA, and has an MOU to provide clinical services to VA Puget Sound patients. Her time was protected for delivering MOVE!+UP, CPRS documentation, documenting clinical data collected during intervention visits, intervention follow-up activities (e.g., care coordination), and ongoing training and supervision. As site PI, she oversees regulatory issues for the Boise site. She will not engage in any other research activities (for example, recruitment, consenting, assessments, or analysis). She has access to VA Puget Sound data sources. She is approved to work in this capacity by the Boise VA.

Boise VA Health Care System
Psychologist, V20 CRH TelePain
500 W Fort St, Boise, ID 83702
Phone: 206-277-1062
Email: Moriah.Brier@va.gov

5.0 Study Procedures

5.1 Study Design

We conducted a two-site hybrid type 1 randomized controlled trial[31] with overweight or obese Veterans with PTSD currently enrolled in VA Puget Sound PTSD care, comparing those randomized to usual care enhanced with enrollment in *MOVE!* (control) versus usual care enhanced with *MOVE!*+UP (intervention). The primary outcome of the trial is 6-month weight loss. Because *MOVE!*+UP also targets PTSD symptoms and long-term weight loss maintenance is an important goal of weight loss treatments,[18] this study will explore effects on secondary outcomes of 6- and 12-month PTSD symptom reduction and 12-month weight loss. To contextualize those findings, Aim 2 will compare intervention and control conditions on several key treatment targets including physical activity, eating behavior, insomnia, depression, and social support. Aim 3 will focus on implementation-related data (e.g., utilization), and assess reach and fidelity. If the trial is positive, we also will assess costs and collect qualitative data to further contextualize Aim 1 findings and inform future implementation efforts if *MOVE!*+UP is effective.

If the trial is positive, we will interview 15 clinical leaders and providers (stakeholders) from VA Puget Sound after 6-month trial outcomes are collected. Because pilot interview feedback from stakeholders most closely connected to *MOVE!*+UP was of greatest value, we will specifically target *MOVE!*+UP facilitators, and *MOVE!* and mental health leaders. Approximately 50-minute semi-structured interviews will be conducted by phone. Each interview will begin by viewing a 6-minute video to orient individuals to *MOVE!*+UP, including information on training, hiring, safety, and findings. We developed the *MOVE!*+UP pilot video within one month of concluding cohort 5, partnering with VA Puget Sound Medical Media, using volunteer staff actors. We will use a similar process after 6-month data have been collected and analyzed. The interview guide (see attached Stakeholder Interview Guide) will include open-ended questions about implementation challenges and facilitators, informed by the five Consolidated Framework for Implementation Research (CFIR) domains: intervention characteristics, outer setting, inner setting, characteristics of individuals, and process. Specific salient constructs will be selected based on our experience in the trial, and additional questions will be tailored based on whether *MOVE!*+UP affected weight and PTSD. Interviews will be audio-recorded and transcribed, with identifiable information redacted and stored separately from identifiers (in secure SFFX folder). As in the *MOVE!*+UP pilot, initial and ongoing training, and periodic quality assurance audits, will be conducted by the PI and research team with input from collaborators as needed.

Risks and Benefits. The primary potential risks for human subjects are minimal and involve those related to breach of confidentiality, lost time, and the stresses associated with these. All procedures are designed specifically to minimize these general and unique risks to Veteran and VA employee participants. Unique risk issues for Veteran and VA employee participants are discussed in a and b below.

<u>a. Veteran participants (Aims 1 and 2)</u>

Therapeutic risks.

The intervention itself poses no undue risks. Participants may have become aware of previously unrecognized health issues that may cause them concern. Change in both physical activity and diet could potentially lead to injury. This risk is highest for high intensity strenuous activity, which was not explicitly encouraged in this intervention. There is slight risk of injury involved with walking, which is part of the *MOVE!*+UP group sessions. We included a safety protocol to address these risks (see attached Safety Protocol). Because *MOVE!*+UP group walking sessions were held in the community, there is a slight increased risk for breach of confidentiality. *MOVE!*+UP arm participants were asked to complete a walking safety audit (see ALT Walking Audit) in their home community prior to participating in their individual neighborhood walks, and the facilitators worked with participants unable to find a safe walking route in their neighborhood.

Research risks and possible benefits.

We anticipated minimal risks to the participants responding to the study questionnaires. The mental health-related questionnaires could potentially induce stress secondary to invasion of privacy. Although unlikely, some respondents may find some questions intrusive or offensive. Participants will be free to skip such questions. Participants may become aware of previously unrecognized health issues that may cause them concern. It is possible that participation in this study will not provide subjects with any direct benefits. While studies of similar treatments have benefited patients by improving their quality of life, no one can know in advance if it will be helpful in a subject's particular case. The results of this study may provide important information regarding ways that health and mental health can be improved for overweight Veterans with PTSD symptoms.

<u>b. VA Employees (Aim 3)</u>

All potential risks to VA employees are research-related as VA employees are not participating in any therapeutic activities. VA employees may feel uncomfortable discussing *MOVE!*+UP and *MOVE!* in Aim 3's qualitative interviews. They may worry that participating, not participating, and/or being candid in interviews will affect their standing at work.

Study Population.

<u>a. Veteran participants (Aims 1 and 2)</u>

Inclusion and exclusion criteria are described in the text below, as well as in Table 4, in **Section 5.4 Inclusion/Exclusion Criteria**. We will conduct a brief chart review for eligibility prior to conducting screening with Veterans using the Phone Screen.

Veterans must have been receiving care within the VAPSHCS system to ensure accessibility to medical records and care coordination, as this trial was being conducted among VAPSHCS patients. In general, to be eligible, participants must have been enrolled in PTSD care. Veterans were instructed during the orientation and informed consent that they should continue to receive medical

and mental health care as determined collaboratively with their providers. Because *MOVE!*+UP is designed to augment standard care and to promote comparable PTSD care across conditions for PTSD analyses, participants had to be engaged in PTSD care, determined by medical chart review (or Veteran self-report if they are receiving non-VA care). Specifically, Veterans must have received VA/DoD PTSD guideline-recommended psychotherapy (e.g., cognitive behavior therapy, Cognitive Processing Therapy, Prolonged Exposure)[11] in two visits with a PTSD diagnostic code assigned in the past three months and have an upcoming mental health visit. Veterans would also be eligible if they had a current prescription of any of the following VA/DoD PTSD guideline-recommended medications: sertraline, paroxetine, fluoxetine, or venlafaxine; prazosin (for the treatment of PTSD-associated nightmares); or nefazodone, imipramine, or phenelzine.[11] Veterans who expressed interest in the study but were not currently enrolled in PTSD care, were informed of study eligibility criteria, and provided with information about how to schedule a PTSD care intake (i.e., they can go through Primary Care (PCMH) or Emergency department and request PTSD care/enrollment). This information was included in a Mental Health Resources sheet that outlines various resources for initiating or reengaging in mental health treatment, as well as the Veterans crisis line. It was reviewed with Veterans expressing need for mental health care at any stage of the research process (and offered to have it mailed). In addition, it is explicitly mentioned in our Phone and Eligibility Screeners as an option to offer to Veterans who express that they are not engaged in care during screening.

Veterans had to be classified with overweight or obesity and meet criteria for current PTSD. A VA primary care provider provided approval to ensure fitness for participation. If we were unable to obtain approval from a VA PCP because they are unavailable/don't respond to the request or the Veteran does not have a VA PCP, another physician who cares for the Veteran was approached for approval (e.g., their psychiatrist).

Exclusion criteria were minimal, including those not fluent in English, those with severe hearing loss or no phone access, those currently participating in gold standard *MOVE!* (although they can participate if only doing Tele*MOVE!* Or self-guided *MOVE!* using the app or website), and those currently pregnant. Also, based on clinical judgment, those with the following conditions were unable to participate: a) acutely exacerbated substance use, mental health, or chronic medical conditions or b) moderate to severe chronic, progressive neurologic conditions such as Dementia. However, those with severe PTSD and co-morbid psychiatric conditions, were included to maximize generalizability.

While those currently enrolled in *MOVE!* were excluded, prior participation was not an exclusion criterion because of the chronic nature of weight management. The study included individuals with mobility limitations because they are common in this population and exercise is often recommended to improve functioning.[59] Based on *MOVE!*+UP pilot data, mobility limitations did not affect participation, nor outcomes.

This study excluded pregnant women because although obese women are discouraged from gaining excessive weight, they should gain 11-20 pounds,[60]

which would affect the primary outcome. If a participant became pregnant during the trial, they would have been able to continue with physician (e.g., primary care provider or gynecologist) approval, and outcomes would be excluded (though this did not occur). Likewise, we would have sought approval for continued participation among those receiving bariatric surgery during the intervention period.

We excluded people who had received bariatric surgery in the past 12 months or people who planned to have bariatric surgery in the next 6 months, based on self-report in the phone screen and/or upon medical record review. This is consistent with other similar trials, such as the RAINBOW trial (Ma et al, Contemporary Clinical Trials, Research Aimed at Improving Both Mood and Weight (RAINBOW) in Primary Care: A Type 1 hybrid design randomized controlled trial (nih.gov)), which co-intervened on depression and weight. In addition, a review by Wolfe et al (Treatment of Obesity: Weight Loss and Bariatric Surgery - PubMed (nih.gov)) shows that weight regain is common at the 12 month mark (meaning participants would benefit from behavioral weight management and would no longer be in the period of weight loss that immediately follows bariatric surgery).

b. VA Employees (Aim 3)
VA Puget Sound (Seattle or American Lake) stakeholders who delivered or supported *MOVE!*+UP (or could in the future) will be eligible. Staff participants will be adult VA employees and will range in age, training, and experience. None will be excluded based on demographic characteristics.

Interventions.

- Control group participants participated in the VA's standard *MOVE!* Program, delivered as part of usual care. *MOVE!* is an evidence-based program for obesity management. The curriculum uses cognitive-behavioral techniques like goal setting and self-monitoring and applying autonomy-supporting counseling like motivational interviewing mostly in in-person group sessions. *MOVE!* control participants participated via VA Video Connect (VVC). Participants were able to express an interest in other MOVE! modalities as well, if preferred, though the study encouraged participation in the group-based modality, to ensure comparability across treatment arms.

  Those randomized to the intervention group received *MOVE!*+UP, a program co-led by a psychologist with specialized PTSD training and a Veteran peer support counselor. It consists of 16 group sessions that involve general *MOVE!* weight loss support (e.g., goal setting, nutrition guidance, activity/diet monitoring). However, to address PTSD-related barriers, *MOVE!*+UP also incorporates CBT elements such as addressing unhelpful thinking patterns, relationship support, and sleep hygiene. Each

session also includes a 30-minute community walk because activity increases retention in *MOVE!*, and to address hypervigilance-based activity barriers through experiential learning. MOVE!+UP also provides two individual dietician visits. As-needed counseling calls and coordination with medical, mental health, and/or social services are also provided. *MOVE!*+UP sessions were offered by VVC and over the telephone using a backup conference line for those who may prefer to dial in and do not want to or cannot access VVC, dietician visits were held by phone or VVC, and the community walk was done individually during *MOVE!*+UP sessions in Veterans' own communities.

5.2 Recruitment Methods

<u>a. Veteran participants (Aims 1 and 2)</u>
We sought to recruit approximately 164 Veterans, in cohorts of 16-20.

Because key eligibility criteria are based on administrative data, the primary strategy was to send opt-out letters to those meeting criteria identified through the Corporate Data Warehouse/CPRS (see attached Recruitment Opt-In Opt-out Letter and Postcard). Research staff called Veterans who opted-in via postcard or by calling the study, or those who had not returned their postcard within 2 weeks.

To obtain our target sample, we sent letters to 3,482 Veterans over the course of the study.

We used additional strategies: Veterans could self-refer, and we reviewed patients scheduled in mental health clinics to alert providers to discuss the study with potentially-eligible patients. Clinicians could send the names and telephone numbers of interested Veterans (who prefer to be contacted by study staff) via encrypted email. Clinicians were instructed to ask permission from the Veteran prior to providing this information to study staff (see provider talking scripts and Provider_Referral_Email_Overview).

Initial eligibility was assessed by phone screen and medical chart review, but confirmed during a virtual enrollment (see ALT Phone Screen). Project staff contacted Veterans by telephone to assess eligibility and interest, and for those who were eligible based on screening criteria, a follow-up virtual eligibility confirmation and enrollment visit, held over phone or VVC (or other approved video conferencing modality should VVC not be working), was scheduled. During that virtual visit, eligibility was confirmed, the previously mailed information statement was reviewed, and verbal consent obtained (confirmed with signature by staff on the Oral Script), and the baseline measures were completed (see ALT Eligibility Measures, ALT Consent, and Baseline Assessment). Further details about consent and assessment procedures are described in **Sections 5.3.a. Informed Consent Procedures** and **5.5.a. Study Evaluations**, respectively.

We sought to ensure at least 20% of the sample included people of color and women Veterans, comparable to the approximately 30% of *MOVE!* +UP pilot participants who were people of color and/or women and demographics of VA Puget Sound overweight Veterans with PTSD. We performed demographic checks and enrollment numbers with each cohort, and altered recruitment strategies when not meeting recruitment goals. During peak recruitment, the PI and coordinator spoke frequently to ensure sufficient recruitment, and to problem-solve issues, at times with input from the larger team. We included a pre-enrollment orientation to increase research activity engagement, based on approaches from prior behavioral trials,[62] which we used in our prior pilot. Orientation materials included expected activities and time commitments for assessments and *MOVE!*+UP or *MOVE!*, and clarified that participants were encouraged to continue engaging in VA mental health and medical care as clinically indicated. Prior to consenting and enrolling, Veterans were asked to commit to all activities and identify personal reasons for doing so (see attached Orientation Script and Materials, including alternative version for pandemic). To facilitate retention we incentivized assessments, obtained alternate contacts and updated locator information regularly, and used a lost-to-follow-up protocol (see attached letters and phone/text scripts).

Subject Payment. Participants were paid $20 for completing self-report surveys and weight (including those who either screen-failed and did not enroll in the study or those who were eligible but declined to enroll in the study during the enrollment visit as long as they returned their accelerometer by mail), $20 for the 16-week satisfaction measure, and $20 for completing accelerometer assessments, for a total of $120. No incentive was given for attending intervention sessions intervention to maintain study generalizability and feasibility of wider adoption.

We believe the above payment structure is appropriate given the type of questions we asked, the duration of time we asked participants to set aside in their schedule, and the duration of time we asked them to remain involved in the study. Payments were paid via grant funds administered by the VA Puget Sound R&D. Subjects were originally paid by check. Checks were be mailed directly to participants by the Department of the Treasury, which could take up to 16 weeks to process. Participants were notified in the information statement and at all assessment visits about the timeline for receiving compensation. As of 8/25/23, payment via check was no longer allowed, due to the nationwide check moratorium.

Participant payments were also made via Direct Deposit. If participants do not have Direct Deposit established but would like to do so, our study staff help subjects get it set up. Participants who receive payments via check were contacted following the check moratorium deadline (9/30/23) to 1) vendorize them in order to set up Direct Deposit (should they choose), 2) be notified of, and given, the choice to select possible gift card payment for future assessments, or 3) be given the option to opt out of receiving study payments completely.

As gift cards have been approved for research subject payment (approved as of September 2023), the study also offers gift cards to those participants who do not want to become vendorized to receive payments via Direct Deposit. For those who opt to receive gift cards, they were given the option of digital or physical gift cards to be emailed or mailed to them, respectively, as an attachment to an encrypted email (see Digital Gift Card Payment Email Template) or mailed with a hard copy letter (see Physical Gift Card Payment Letter Template).

Should the study receive notification of canceled payment(s) from the VA Puget Sound R&D fiscal administrators, study staff work with Veteran and fiscal administrators to have Veteran submit an updated VA10091 form in order to process their study payment(s) (see Request for VA10091 Letter).

b. VA Employees (Aim 3)

We will invite VA Puget Sound stakeholders to participate in a single qualitative semi-structured interview that takes place by phone or in person. Because pilot interview feedback from stakeholders most closely connected to *MOVE!*+UP was of greatest value, we will specifically target *MOVE!*+UP facilitators, and *MOVE!* and mental health leaders. We will use snowball sampling techniques to identify additional participants. Sampling and recruitment will proceed in an iterative fashion until thematic saturation is reached.

We will establish communication with a point-of-contact at each VA Puget Sound site to introduce the study and identify the optimal recruitment and interviewing methods for minimizing disruption of patient care. Based on their input, interviews will be arranged via email, telephone, Webex messaging, and/or through on-site recruitment (see various attached Stakeholder recruitment and engagement materials: email, Webex /phone recruitment, scheduling, confirmation and follow-up scripts, and flyer/overview). They will be given the opportunity to opt into study participation, or to decline. All potential stakeholder participants will receive the Information Statement (see attached Information Statement) that describes the study.

Should they express interest in participating and decide to opt-in, eligible stakeholders will then be followed-up with via their preferred method to schedule the interview over the phone or via Webex (unless there are circumstances that prohibit these modalities being used) at a convenient time for the participant. Once the interview is scheduled, a confirmation email will be sent, reminding participants about their scheduled interviews and encouraging them to print the attached notes sheet to have on hand during the interview while watching the *MOVE!*+UP video (see *MOVE!*+UP Video Notes Sheet). An Outlook calendar invite will also be sent to all participants with the time/date and if relevant, location (e.g., the participant needs us to reserve a sound-enabled computer, quiet room for the phone interview). Relevant instructions from the email scripts and attachments will also be included on the calendar invite.

Because all recruitment and study activities take place within VA technology and space, recruitment materials specify employee requirement for participation, and participant role and employment information is collected prior to the interview, a formal eligibility screen will not be conducted. If at any point during the recruitment or study phase it becomes clear that the participant is not a VA employee, study staff will thank them for their time and terminate participation.

We will seek verbal consent and will record them providing it, without identifiers, at the beginning of the audio-recording that will be taken during the interview. Verbal consent is proposed instead of written consent because pilot employee stakeholders appreciated the straightforward phone consent and interview procedures. Using a verbal consent approach with employees under these study's conditions is common and ethical. The questions that will be posed and procedures used are minimal risk with no potential impact on employment.

This study involves no procedures for which written consent is normally required outside the research context given that it is common for clinicians and other stakeholders to provide their input on various clinical programs. We will seek a waiver of documentation of consent from the VA Puget Sound IRB.

Subject Payment. Consistent with VA policy, stakeholder participants (as VA employees) will not be provided with compensation for participating in this study.

Occasion Cards. To ensure study engagement and participant retention throughout the study, the study staff inquired about specific events that are meaningful to the participants. During those specified times, study staff sent a study approved card related to the event (see occasion reminder cards). Study cards have been designed for holidays (religious and non-religious), positive life events (i.e. birthday, anniversary, etc), study completion, and tragedy events (i.e. loss of a loved one). Submission of these cards served the purpose of keeping subjects engaged, as well as recognition of momentous occasions in which the study wishes to acknowledge. The staff sent out no more than 6 cards per subject to reduce study contact fatigue. The cards were primarily selected based on occasions identified as being meaningful to the Veteran during the enrollment visit after the Veteran has provided verbal consent to participate (see Eligibility screener).

We note in the thank you for participation card that we will follow-up with the Veteran at the end of the trial to share final trial results. A letter and materials will be submitted to the IRB toward the end of the study prior to sending the post-trial findings letter/materials.

5.3 Informed Consent Procedures

Consent was obtained by a trained research staff member who meets all facility education requirements in the protection of human research subjects or by one of the study investigators. Study staff received thorough training on informed consent procedures from the study PI. The Oral Scripts that document verbal consent has been obtained for

participation were sent to the RCO using standard compliance procedures (encrypted email).

a. Veteran participants (Aims 1 and 2)

Study staff contact Veterans by telephone to assess eligibility and interest. For those eligible based on screening criteria, a follow-up virtual eligibility confirmation and enrollment visit, held over phone or VVC (or other approved video conferencing modality should VVC not be working), was scheduled. The Information Statement was mailed to the Veteran and was reviewed over the phone or VVC using our Oral Script that includes a protocol and space for obtaining staff signature to confirm verbal consent was provided. Staff ensured that participants were given an opportunity to ask questions about all aspects of the study, and that they understood what study participation will involve. Potential participants were informed that their decision regarding participation will in no way affect the services they receive, and that they may decline to answer any questions or decline any services offered as part of the project. Declining any one service would not affect their ability to receive other services from the project. They also were informed that this study is a randomized trial and that they hadan equal chance of receiving MOVE! or MOVE!+UP. Veterans also provided verbal consent for Use of Picture and/or Voice during the screening process, Study staff also documented whether or not they provided verbal consent to participate over the phone or by VVC.

Staff also used this time to discuss the Group VVC Telehealth consent agreement, which details the use of VVC to deliver the *MOVE!* and *MOVE!*+UP group sessions (see ALT Group VVC Telehealth Agreement).

b. VA Employees (Aim 3)
For the stakeholder interview phase of our study we request a waiver of documentation of consent because this is a low-risk study, and it would be impractical to obtain written consent from all study participants given that nearly all recruitment activities will take place over email and the interviews will take place over the phone. As such, nearly no in-person contact will take place as part of these stakeholder interviews. These recruitment and interview procedures are designed this way to increase convenience for the stakeholders, particularly given we plan to recruit stakeholders outside of the Seattle VA, where the research staff are located. Moreover, participants will be fully informed of the procedures, risks and benefits of the study through the information statement and will be reminded of the voluntary nature of study procedures at the start of the interview. Verbal consent will be obtained prior to the interview.

Using procedures from our pilot, we will provide a thorough overview of procedures and potential risks and benefits, including the fact that participation or lack thereof will not affect employment, through an IRB-approved written information statement emailed to employees prior to performing the interview (see Information Statement). We will seek verbal consent and will record them providing it, without identifiers, at the beginning of the audio-recording that will be taken during the interview.

Verbal consent is proposed instead of written consent because pilot employee stakeholders appreciated the straightforward phone consent and interview procedures. Using a verbal consent approach with employees under these study's conditions is common and ethical. The questions that will be posed and procedures used are minimal risk with no potential impact on employment. This study involves no procedures for which written consent is normally required outside the research context given that it is common for clinicians and other stakeholders to provide their input on various clinical programs.

5.4 Inclusion/Exclusion Criteria

We also discuss inclusion and exclusion criteria in **Section 5.1 Study Design** above when discussing the study population. We also summarize our criteria for the Veteran participants (Aims 1 and 2) in Table 4:

Table 4. Inclusion/Exclusion Criteria

<u>Inclusion Criteria:</u>	<u>Exclusion Criteria:</u>
- Must have active VAPSHCS medical record.<br>- PTSD Diagnosis: standard criteria for PTSD Checklist for DSM-5 (PCL-5): lifetime<br>-Experience of trauma and a score of ≥33[61]<br>-Overweight or obese based on Body Mass<br>-Index (BMI) of ≥ 25 kg/m2<br>-VA Primary Care Provider or other<br>physician approval<br>-Willing to participate in all intervention or<br>control and assessment activities<br>-Enrolled in PTSD treatment	-Not fluent in English, severe hearing loss, no phone access<br>-Current MOVE! participation<br>-Past year bariatric surgery or planning to have bariatric surgery in next 6 months<br>-Current pregnancy<br>-Based on clinical judgment, would be unable to participate because of a) acutely exacerbated substance use, mental health, or chronic medical conditions or b) moderate to severe chronic, progressive neurologic conditions such as Dementia.<br>- Can't safely weigh themselves using the mailed study scale and/or have a baseline weight >440 lbs

5.5 Study Evaluations

<u>a. Veteran participants (Aims 1 and 2)</u>
Per IRB-approved procedures, this study involved several assessment components: initial phone screen to ascertain interest and basic

inclusion/exclusion eligibility, eligibility confirmation, consent and enrollment, baseline assessment, 16-week satisfaction questionnaire, and 6-and 12-month follow-up outcome assessments.

Table 5. Quantitative Measures for RCT: Covariates, and Aim 1 and 2 Outcomes

Domain	Measure
***Baseline Covariates Associated with Weight or PTSD** (will be adjusted in [a]weight or [b]PTSD analyses)*	
African-American race/ethnicity[a]	Self-reported African-American race/ethnicity[80]
Aim 1: Test MOVE!+UP's Effects on Weight (Primary) and PTSD (Secondary) Outcomes	
Primary outcome: weight loss	Standardized protocol and equipment at baseline and 6 months. Will also explore 12-month weight loss in secondary analyses.
Secondary outcome: PTSD symptoms	PTSD Checklist for DSM-5[61,77] PTSD symptomology will be recorded at baseline, 6 months, and 12 months.
Aim 2: Exploratory Changes on Treatment Targets from Baseline to 6 months	
Diet quality and eating habits	Starting the Conversation[82] and PACE eating habits measure
Night eating	Night Eating Questionnaire[83]
Emotional eating	Emotional Overeating Questionnaire[84]
Binge eating with purging vs binge eating without purging vs no binge eating	PHQ-15[85] modified to reflect DSM-5 changes plus items from PACE eating habits
Two outcomes: Average physical activity (light, moderate, and vigorous; moderate and vigorous)	Actigraph accelerometers, gold standard[78,79]
Social support	Helpful and unhelpful behaviors from others for activity/diet[86,87]
Insomnia severity	Insomnia Severity Index[88]
Depression symptoms	PHQ-8[89]
Internalized Weight Bias	Modified Weight Bias Internalization Scale

Phone screen (5-20 minutes; see ALT Phone Screen).

Study personnel provide an overview of the study and, for those still interested, conducted an initial pre-consent screening, which consists of questions corresponding to inclusion criteria/exclusion. To proceed to the in-person eligibility assessment phase, the potential participant must 1) be age 18 or older, (2) be a Veteran of the US military, (3) be a VA Puget Sound patient, (4) screen positive on the 4-item Primary Care PTSD Screen (PC-PTSD) as a likely indication of PTSD, (5) report a BMI that is classified as obese or as overweight, (6) be enrolled in PTSD care, (7) be motivated and willing to participate in all intervention or control and assessment activities, (8) be fluent in English, (9) have telephone access, and (10) receive VA primary care provider approval. The potential

participant must also deny (1) severe hearing loss, (2) being pregnant, (3) current participation in *MOVE*!, and (4) past year bariatric surgery or planned for the coming 6 months. We also assessed if the potential participant can safely weigh themselves at home using a study-provided scale. Should they be unable to weigh themselves safely at home using the mailed study scale during the virtual eligibility assessment visit (e.g., they are fully wheelchair-bound), they were considered ineligible, as we had no means of safely weighing them in person using a wheelchair-accessible scale. In addition, veterans were excluded during the phone screening if they report that they have a baseline weight >440 lbs.

We also assessed access to VVC enabled technology, secure messaging, and permission to email materials (and instructions for that process if used via one-way email from study staff to participants, using the already IRB-approved text scripts to craft messages; see Telehealth Team Guidance Outlook email).

The purpose of the pre-consent eligibility screening was to quickly determine which candidates are most likely to qualify, thus eliminating the need to conduct consent and full eligibility assessments for all potential participants. If potential participants are found to likely be eligible following the pre-consent eligibility screening, the study coordinator scheduled the virtual eligibility and baseline assessment visit, which was conducted virtually, ideally via VVC (but via phone if preferred or Veteran cannot or does not want to use VVC or other approved video conferencing modality should VVC not be working), allowing adequate time for study materials (and CVT connect program equipment to be mailed to participants who need it for VVC sessions and assessments).

The following also occurred:
a) Participants who did not have an internet-enabled tablet or computer from which they can join study visits and intervention sessions were be sent one through the CVT connect program by study staff placing a consult in their chart, using standard procedures.

b) Participants were mailed several study materials, including the study-provided scale; the Information Statement; orientation materials and the group VVC Telehealth agreement (discussed further below, see ALT Orientation Script & Materials and ALT Group VVC Telehealth Agreement); a hard copy of the baseline measures (see Baseline Assessment) accelerometer and accelerometer instructions and the hard copy logs (see Activity Monitor Instructions and Activity Monitor Daily Tracking Log and Daily Food Beverage & Activity Log); and a postage-paid envelope for returning the accelerometer to the study team.

If a subject preferred to receive the materials in person, rather than by having USPS/UPS deliver study materials to their home (or being unable to receive USPS/UPS deliveries), study staff offered to meet with the Veteran in-person at the VA in a clinical setting for a face-to-face visit in order to hand off the materials. All VA policies with regard to masking and

distancing during the SARS-CoV-2 pandemic were followed during this interaction. This option was also offered to enrolled study subjects for all follow-up assessments and visits. Staff were sure to confirm identity of the participant prior to interacting and did so in a private location to protect the identity of the individual.

c) Study staff confirmed approval with Veteran's PCP or other physician for participation following the screening and eligibility visit, so it was possible someone's physician could disallow participation after they've provided verbal consent to participate in the study (see ALT PCP Approval Template). If the potential participant was scheduled for an assessment less than seven days after they completed the phone screen, or if the participant was scheduled to be assessed within 48 hours but the approval had not been received, study staff sent an email to the PCP or other physician (see ALT PCP Email template). For Veterans who did not have a PCP but who are established with another physician for care (e.g., psychiatry), we sought that physician's approval using the same procedures used for PCP approval.

We had IRB approval to allow potential participants to participate in *MOVE!*+UP who didn't have VA PCPs or other physician. In those cases, we would obtain community PCP approval of *MOVE!*+UP participation using the standard PCP approval template, obtained through fax or other secure means after a release is signed by the participant. The PCP approval template would be entered into CPRS with Dr. Hoerster as the signer and a note at the top of the CPRS note saying "this is for Veteran's outside VA PCP to review." This note would be sent to the participant by study staff after obtaining the ROI to take it to their PCP for PCP signature and returned to study staff by mail or using secure messaging.

Eligibility assessment (15-30 minutes; see ALT Eligibility Measures). The follow-up eligibility assessment was conducted virtually, ideally by VVC, at a time that allowed study materials to have been mailed to the participant. The various components of the assessment visit can be completed on different dates as needed (as long as all components are completed within the acceptable assessment visit window) to best accommodate Veterans. Ideally, all components would be completed on the same day during the same VVC visit.

Prior to the visit, study staff confirmed receipt of mailed materials and the study scale and would troubleshoot any issues with VVC connection. Should VVC be having issues, staff would send the participant a link, via one-way text or email to the participant, to another approved video conferencing modality from which they could join the assessment visit (see Text Scripts).

Study participants were asked to weigh themselves with the study-provided scale to confirm they meet the eligibility criteria for the study (overweight/obese status; BMI of at least 25). Height for BMI calculation was based on CPRS-based information.

They also completed a current assessment of PTSD symptoms, administered by study staff. Current PTSD was confirmed during this visit as indicated by endorsing at least one lifetime traumatic event on the Life Events Checklist for DSM-5 (LEC-5) and based on their score on the PTSD Checklist for DSM-5 (PCL-5). A provisional PTSD diagnosis can be made by following the DSM-5 diagnostic rule, which requires at least moderate severity endorsement (a score of at least 2) on the following: 1 B item (questions 1-5), 1 C item (questions 6-7), 2 D items (questions 8-14), and 2 E items (questions 15-20). In addition, to meet PTSD diagnosis criteria, the individual must obtain a score of ≥33 on the PCL-5. As long as they continue to meet those inclusion criteria, they may be officially enrolled in the study and designated for randomization.

Respondents who do not meet eligibility criteria were offered a resource list (see Mental Health Resources Handout), and given *MOVE!* contact information and told to follow-up with their primary care provider or mental health treatment coordinator for mental health treatment or other care coordination needs.

For those who were eligible, we collected contact information (e.g., address, telephone number, and whether they provide permission for us to text reminders to a mobile telephone number) and contact information for at least one friend or family member who typically would know the whereabouts of the participant should study staff have difficulty making contact will be recorded. Eligible patients were invited to learn more about the study, and proceed with orientation (process described below) and verbal consent (process described in **Section 5.3.a. Informed Consent Procedures**). After providing verbal consent to proceed with enrollment, they completed baseline measures, and be randomized to either *MOVE!* (control) or *MOVE!*+UP (intervention).

Orientation content (10-15 minutes; see ALT Orientation Script & Materials). Study staff spent 10-15 minutes during the enrollment session prior to reviewing the consent orienting participants to the process of research participation and the importance of accepting randomization group and following through with follow-up assessments, using the mailed orientation materials. This is to better outline study activity expectations, including the total amount of time expected in weekly activities for the program and study. We also offered an opportunity for participants to consider the pros and cons of participation, a standard motivational interviewing technique. They also were asked to consider whether they can make two personal commitments: 1) to working on their health and weight and 2) to participate in the study procedures. Because this conversation took place during the virtual enrollment visit, it was not used to attract people to the study but rather to fully inform them about all involved in participation, including randomization into the control versus the intervention group, therefore enhancing the informed consent process (i.e., this process was not trying to unduly influence people to enroll in the study, rather give them more opportunity to consider NOT enrolling if they

don't think they can follow through with the study's expectations). Materials and plans are based on a model found to be very successful in other interventions facing difficult retention situations, and used in our pilot work. Participants then proceeded to informed consent (detailed process described in **Section 5.3.a. Informed Consent Procedures**). After providing verbal consent to enroll in the study, they proceeded to complete baseline measures. They were then randomized, and notified of their assignment and details/logistics, and reminded that materials would be mailed to them for their program (*MOVE!*+UP or *MOVE!*).

Exploratory measures, accelerometer, and logs (45 minutes). To ensure that all exploratory behavioral and psychological measures were collected prior to randomization, study staff administered the questionnaire during the virtual visit, starting with cohort 2 (prior to that, participants were able to complete it on their own and mail it back, leading to some lost baseline questionnaires). Administering the questionnaire in real-time helped ensure the data were collected in a timely and reliable manner.

Participants were asked to wear an accelerometer and complete associated logs (see Activity Monitor Daily Tracking Log and Daily Food Beverage & Activity Log) for one week following the baseline visit. Participants received a reminder mid-week to complete the logs, and at the end of the week to return them in the pre-addressed, pre-paid envelope. Study staff reminded the participant to remove the accelerometer and facilitated expedited return via UPS. If accelerometer data were invalid upon return (e.g., empty data) or not returned, we occasionally asked a participant—but did not require—to re-wear an accelerometer. If the accelerometer and logs had not been returned one week after the participant was scheduled to take it off, study staff would call or text the participant to remind them to return it. If it still had not been returned after another 3-5 days, staff texted or emailed and called again and sent a reminder letter (see letters and text/email reminder scripts).

Should a subject prefer to return the materials in person, rather than by having USPS/UPS pick up study materials from their home, study staff offered to meet with Veteran in-person at the VA in a clinical setting for a face-to-face visit in order to hand off the materials. All VA policies with regard to masking and distancing during the SARS-CoV-2 pandemic were followed during this interaction. This option was also offered to enrolled study subjects for all follow-up assessments and visits. Staff were sure to confirm identity of the participant prior to interacting and in a private location to protect the identity of the individual.

Randomization and group logistics (~15 minutes). Participants who completed verbal consent and baseline measures were randomized to either *MOVE!* (control) or *MOVE!*+UP (intervention). Those randomly assigned to the control condition were scheduled directly into *MOVE!* VVC, and we made reminder calls and sent letters to promote attendance of initial sessions (see ALT Letters and Phone/text Scripts). Those

randomized to *MOVE!*+UP were instructed to not participate in *MOVE!* during the study. Study staff went over the details of group session dates/times and how to access the respective VVC groups and will walk randomized participants through that process. They were reminded that a participant manual would be sent to them for *MOVE!*+UP or *MOVE!* in the mail prior to the group sessions start. *MOVE!*+UP participants were encouraged by study staff to map out a safe and pleasant walking route using the audit that sent to them with their manual before their first session (see ALT Walking Audit).

Follow-up assessments (30-60 minutes) The 16-week satisfaction questionnaire tailored for MOVE!+UP and MOVE!, was mailed to be completed on hard copy, or emailed with a personalized Qualtrics URL link to be completed electronically, to participants at approximately 15 weeks so they could complete and/or return it in a postage-paid, pre-addressed envelope at approximately 16 weeks. If the survey was not received, staff contacted the subjects via phone to obtain the 16-week data. Participants received follow-up reminder letters, emails, or phone calls if the 16-week assessment had not been completed. If subjects did not return the measure via mail or complete the measure via the emailed personal link, they had the option to complete the assessment via phone.The measure assesses 30 *MOVE!*+UP content and structure features with a Likert satisfaction scale, with space for Veterans to suggest changes for each. We modified that measure to remove the specific *MOVE!*+UP domains to assess *MOVE!* satisfaction among control participants. We sent emails to individual participants only one email at a time (rather than blind carbon copy) to avoid disclosing the email addresses of other consented subjects.

The 6-month assessments mirror the baseline assessment procedures. The various components of the follow-up assessment visits could be completed on different dates as needed (as long as all components are completed within the acceptable assessment visit window) to best accommodate Veterans. Ideally, all components would be completed on the same day during the same VVC visit. Ideally the subject would show their study issue scale readout to staff during the VVC session. If this is not possible, they may take a photo of the scale reading with their cell phone and text it to a staff member's GFE iPhone. This allows for the message to be encrypted. Staff do not text the Veteran back. This will only be done in the rare circumstance that the scale cannot be visualized.

12-month follow-up assessment (15-30 minutes). At the final assessment visit at 12 months, all participants have a final weight measurement taken. PTSD symptomology, non-VA utilization, Adverse Events, and pregnancy will also be recorded at this time. All participants will be thanked for their participation in the study. This visit takes place

via VVC (or other approved video conferencing modality should VVC not be working).

Manually tracked and administrative data utilization, and process variables.

For Aim 3, which seeks to place Aim 1 and 2 findings in context and identify areas needing refinement for future implementation, we will capture various process and administrative data.

1. *Intervention only: MOVE!*+UP group and phone session attendance, no shows, and cancellations, including participation in the walks and reasons for non-participation in any aspect of *MOVE!*+UP sessions.
2. *Intervention only:* We will assess time and costs associated with all *MOVE!*+UP activities. Personnel time will be captured during start-up activities like initial training, and once *MOVE!+UP* is running, for one week each quarter, modeled prior research.[96] Research and clinical personnel will enter time spent that week on *MOVE!+UP* training, recruitment, supervision, delivery, and documentation into a system developed by Liu et al.[97] To estimate personnel costs, we will multiply hours each research and clinical personnel spent by their hourly wage rate, as recorded in the VA Personnel and Accounting Integrated Data, or imputed using Bureau of Labor Statistics data.[98] Co-I Wong is applying these methods to analyze implementation costs in a QUERI partnered evaluation. Facilitators and research staff also provide observations and input throughout the course of the study to further assess and inform implementation= (in secure SFFX folder).

3. *For intervention only:* The following information was collected on a basic spreadsheet (see weight goal tracker: weigh-in weights; physical activity and diet quality goals and goals progress (whether meeting them fully, partially, or not); and any additional relevant information in an open-ended comments box. We also copied, scanned, and provide feedback on weekly diet and activity logs, again to be used for clinical purposes and to be evaluated as an exploratory diet quality measure. Both the activity/diet log copies, and the weekly weight/goal tracking documents were stored securely in a locked cabinet, with only a subject ID listed.

4. *For all participants:* We will capture CDW/CPRS administrative utilization data from 12 months prior to baseline through 12 months post-baseline, including MOVE! visits, dietician visits, primary care, mental health group and individual psychotherapy, and psychiatry care. We also will assess prescription of psychiatric and obesogenic and leptogenic medication from VA pharmacy files. Outpatient utilization will be drawn from administrative data, classified using an algorithm developed by a study team including Co-I Wong that defines groupings of CPT codes and provider specialty codes.[99] Inpatient care will include all cause, psychiatric, and ambulatory care-sensitive condition hospitalizations, as identified in prior studies.[100,101] We also

will pull bariatric surgery from the EHR from baseline through 12 months post-enrollment to be included in a previously planned sensitivity analysis.

If the trial is positive, costs for encounters in these categories will be measured using the VA Managerial Cost Accounting (MCA) system, adjusted for inflation. We also will capture # of Veterans eligible, ineligible, contacted, recruited and retained, and psychiatric and medical conditions associated visits in the year prior to and 12 months following enrollment (pulled through administrative data).

Starting with cohort 4, we informed participants at enrollment into the study that administrative data will be pulled even if they cease participation in the study, to ensure we have sufficient power to conduct exploratory analyses examining these administrative data-based outcomes, while introducing no additional burden to study participants.

For participants from cohorts 1-3 who withdrew from the study, administrative data will be pulled up through the date of withdrawal. However, participants from cohort 2 and 3 were asked whether they were willing to allow us to pull data from the medical record through 15 months using our phone script. We documented their response in a NTF, submit it to the IRB, and document their decision in our database.

Post-participation activities Several activities take place following the conclusion of Veterans' study participation. First, we sent an IRB-approved closeout letter thanking Veterans for their participation in the study. Also, once the study's primary findings are published (in approximately 2025), we will send a thank you mailing to provide findings to participants. Study staff will pull participants' current mailing addresses from CPRS to send thank you letters and hard copies of the study's primary outcomes paper. Participants will not be asked to contribute any additional data or effort to the study. The purpose of that mailing will be to inform participants of our findings and thank them for their participation. We view this concluding step as essential to sharing findings with some of our most important collaborators in the research process: the study participants themselves.

<u>b. VA Employees (Aim 3)</u>

Semi-Structured Stakeholder Interviews (50 minutes; see attached Stakeholder Interview Guide and *MOVE!*+UP video). If the trial results are positive, semi-structured qualitative interviews will be conducted with VA Puget Sound employees to assess implementation barriers and facilitators. Interviews will last approximately 30-40 minutes, with the whole process taking approximately 40-50 minutes because participants will watch a 6-minute *MOVE!* +UP video describing *MOVE!*+UP's key features and record their reactions on a provided notes sheet (see Stakeholder Interview Guide). Because stakeholders will represent different roles, certain questions will be tailored to their particular group. Participants also will be asked several basic demographic questions (e.g., profession, length of VA employment) to characterize the sample.

We will interview 15 clinical leaders and providers (stakeholders) from the two VA Puget Sound trial sites after 6-month trial outcomes are collected. Approximately 50-minute semi-structured interviews will be conducted by phone by our research coordinator, experienced in qualitative methods. Each interview will begin by viewing a 6-minute video to orient individuals to *MOVE!*+UP, including information on training, hiring, safety, and findings.

The interview guide will include open-ended questions about implementation challenges and facilitators, informed by the five CFIR domains: intervention characteristics, outer setting, inner setting, characteristics of individuals, and process.[95,103] Specific salient constructs will be selected based on our experience in the trial, and additional questions will be tailored based on whether *MOVE!*+UP affected weight and PTSD. Interviews will be audio recorded and transcribed, with identifiable information redacted and stored separately from identifiers (in secure SFFX study folder).

5.6.a. Intervention Content and Delivery.

The curriculum for the 16 two-hour MOVE!+UP group sessions is in Table 6 below, with sessions 1-4 on basic weight loss, and 11-16 on maintenance. While CBT elements are included throughout, sessions 5-10 are dedicated to addressing PTSD-related barriers through challenging unhelpful thinking, problem-solving, promoting relationships and community engagement, and sleep hygiene. Facilitators use behavioral counseling skills such as motivational enhancement.[37] All MOVE! materials were included, but many were modified to allow for PTSD content. (See attached Participant and Facilitator manuals). MOVE!+UP is led by a Veteran peer support counselor in recovery from mental illness, a model prioritized by OMHSP and VA Mission Act,[51] and a psychologist who provides specialized PTSD care. MOVE!+UP pilot observations demonstrated value in the psychologist addressing PTSD-focused topics (e.g., sleep), and the peer counselor addressing those outlined in the peer support toolkit (e.g., social/community engagement, personal recovery).[27] Facilitators alternate leading less specialized sections (e.g., weekly check-in). Two individual dietician visits are provided, and counseling calls are offered as needed.

**Week**	**Content (***Content unique to *MOVE!*+UP in bold***)**
1: Orientation/*MOVE!*+UP Overview	Energy balance, goal setting, behavioral strategies
2: Healthy Eating to Tip the Scale	Healthy plate, portion control, nutrition facts/labels
3: Physical Activity (PA) to Tip Scale	PA benefits & types (e.g., strength training); overcoming barriers
4: Eating Mindfully and Problem-Solving Barriers	**Experiential mindful eating exercise,** hunger/fullness scale, mindful eating tips, snack ideas, and problem-solving barriers
5: Coping w/ PTSD to Pursue Health	**General coping, thought challenging**, grocery shopping tips
6: Pursuing Wellness	**Sleep assessment and improvement plan**, finding joy, cooking
7: The Role of Relationships in	**Benefits of relationships for health/PTSD; strategies for**

Healthy Living I	**enhancing relationships to improve health;** meal planning
8: Relationships in Healthy Living II	**Family/friend welcome; communication; relationships & PTSD**
9: Community Engagement	**Community engagement benefits/tips, problem-solving review**
10: Health at any Size	**Ways to enhance body image; thought challenging**
11: Moving Toward Maintenance	Overcoming weight plateaus, adding challenge
12: Finesse your Food	Review strategies, e.g., healthy plate/nutrition facts
13: Stay Active!	PA motivators, personal values, long-term planning
14: Handling Slips and Setbacks	Relapse prevention, review of key diet tips. **Thought challenging.**
15/16: *MOVE!*+UP Progress Review	Review of progress and how to continue gains going forward.
1-16: ***CBT for PTSD***; set and track activity, diet, and weight goals; and experiential learning activities.	

Table 6. Curriculum for *MOVE!*+UP Group Sessions

Information was recorded in CPRS using standard procedures because these are clinical visits, likewise for MOVE! visits for the control condition.

1) *MOVE!*+UP sessions were offered by VVC and over the telephone using a backup conference line for those who may prefer to dial in and do not want to or cannot access VVC, dietician visits will be held by phone or VVC, and the community walk will be done individually during *MOVE!*+UP sessions in Veterans' own communities. In the highly unlikely case where VVC was not working, we had approval to switch to another VA-approved modality as listed in the Telehealth memorandum, titled "Use of Video Communication Technology Under COVID-19 (VIEWS 02576895)" and dated March 19, 2020.

2) Minor modifications were made to the facilitator and participant manual (see ALT Session 1 Facilitator Manual and ALT Participant Manual) from that used during the pilot to reflect information relevant to VVC delivery and recommendations appropriate during the pandemic and social distancing recommendations. Perhaps the biggest change is removing the family/friends invitation session so that it remains focused on social support but non-participants won't join (since that adds another layer of complexity for ensuring confidentiality). This is unlikely to have a major impact given no family or friends attended that session during the *MOVE!*+UP pilot.

3) We relied on phone, secure messaging, and VA's BHL Touch program for sharing clinical datapoints back and forth between facilitators and Veterans in *MOVE!*+UP, including weekly weighing, PTSD symptom assessments, diet/activity logging, photos of food labels and walking routes, and general support. Veterans were also given the option of sharing information like their weight during the group, just as is done in standard *MOVE!*. We attempted to set all study participants up with secure messaging after study enrollment to facilitate this important data sharing.

4) During each session, instead of the 40-minute facilitator-led group walk around the VA campus that was done during the pilot, participants were asked to walk in their own communities. Before the first session, they were asked to conduct walking safety audits in their community to ensure they have a safe

walk, using the walking audit form (see ALT Walking Audit). Those who weren't able to find a safe walking route in their immediate neighborhood for the walking component were encouraged to work with facilitators to identify other activities they could do during the 40 minutes of the walking component. We provided detailed instructions on how to walk safely, including how to handle emergencies, in the walking safety handout in the additional resources at the back of the participant manual. These safety promoting considerations are reviewed thoroughly in the first couple of sessions and then reiterated in brief at each subsequent session just prior to the walk

MOVE!+UP has numerous procedures for ensuring safety and confidentiality, and addressing other barriers to walk participation. Protocols for handling medical and mental health emergencies were developed with mental health leadership and physician Co-Is (see attached Safety Protocol).

5) We offered for Veterans to call into the backup conference line during the 40-minute walk in order to receive any additional support and build cohesion.

Training and Quality Control. The PI, a licensed clinical psychologist who developed *MOVE!*+UP and provided supervision in the pilot, trained and supervised peer and psychologist facilitators, with the goal of ensuring *MOVE!*+UP was delivered as intended and with high quality, optimizing *MOVE!*+UP benefits and validity and reproducibility of trial findings.[65,66] Training and quality control procedures, used successfully in the *MOVE!*+UP pilot, incorporated elements used nationally by NCP to train and support *MOVE!* facilitators and by OMHSP to train peer support counselors[27] and evidence-based PTSD treatment facilitators.[54]

<u>Initial Training and Ongoing Supervision.</u> Prior to delivering *MOVE!*+UP to participants, facilitators built health behavior intervention skills and relevant knowledge, and familiarized themselves with *MOVE!*+UP content and structure. They reviewed the general structure and specific content for each session, as outlined in participant and facilitator manuals developed for our pilot. Facilitators participated in the online *MOVE!* Talent Management System (TMS) training, and NCP's nationally-disseminated TEACH for Success program, a one-day program that teaches evidence-based health coaching and motivational interviewing skills to VA primary care personnel.[67] To promote safety and confidentiality, they took VA courses in basic life support; prevention and management of disruptive behavior; and information security; and were oriented to *MOVE!*+UP protocols for handling medical and mental health emergencies and identifying safety threats during the off-site walks. Peer support and psychologist facilitators role played in at least four audio-recorded sessions with volunteer staff acting as standard Veterans.[65,66] Prior to delivering *MOVE!*+UP to RCT participants, facilitators must deliver sample *MOVE!*+UP content as intended and with high quality (≥ 80% of components)[65,66] using the standard rating forms we developed for each *MOVE!*+UP session during the *MOVE!*+UP pilot (see attached Fidelity form sample). Based on the *MOVE!*+UP pilot, we anticipated initial facilitator training would take a total of approximately 15 hours

over the course of two months. The Dietician received approximately two hours of brief training to orient them to *MOVE!*+UP logistics and equip them to address the needs of Veterans with PTSD. Once facilitators are trained, the PI listened to audio-recorded group sessions and rated fidelity with the standard forms created for the pilot, initially for at least 50% of sessions.[65,66] Feedback was provided to facilitators during weekly supervision based on these ratings, and logistical issues were addressed. Study staff also listened to 20% of study sessions for fidelity. Fidelity ratings and qualitative input from supervision will be summarized and reported in study products.

Peer Counselor Training and Competency Assessment. Because there are unique training needs for this role, peer support counselors received additional training, guided by the VA peer support implementation toolkit.[27] Peer counselors often facilitate groups and share their recovery experience but are not intended to replace or provide psychotherapy. Ongoing training and supervision focused on clarifying roles and responsibilities and the importance of maintaining boundaries and confidentiality. A national standard in VA, any new *MOVE!*+UP peer counselor completed 20 hours of online OMHSP training, after which they had to pass a general competency test. They also participated in the national bi-monthly training webinars hosted by OMHSP.

5.6.b. Data Analysis

Power and Sample Size Calculations. This study is powered on weight change at six months, comparing intervention and control conditions. We used formulas specified by Friedman et al. for use in clustered randomized controlled designs.[70] Sample size was calculated for continuous outcomes and inflated to account for clustered randomization, using the inflation factor $1 + (m - 1)\rho$ for cluster size m and intraclass correlation ρ, and attrition. Based on outcomes in prior weight loss trials,[18] **we expect those in MOVE!+UP to lose 12 pounds on average, which would likely correspond to clinically meaningful weight loss (5% of baseline weight).**[18] The specified expected weight loss is plausible, given *MOVE!*+UP pilot participants lost an average of 14 pounds, corresponding to 6% of baseline weight. We expect those in the control condition to achieve weight loss of 3.6 pounds on average consistent with Veterans with PTSD who participate in *MOVE!*, according to administrative data.

In the power calculation, the standard deviation of the outcome was assumed to be 13 based on previous findings. We calculated this value based on the marginal SD of 50 for baseline weight observed among Veterans with PTSD in *MOVE!*,[19] between baseline and 6-month weight observed in the MOVE!+UP pilot (comparable to other weight loss effectiveness trials).[71] Comparing these outcomes, an 8.4 pound difference in weight lost corresponds to prior behavioral weight loss trials[18] and would yield a medium Cohen's d effect size of 0.65.

Table 7 displays the sample size required for varying cluster sizes *m* and intraclass correlation coefficients ρ assuming 90% power. The sample size of N=*164* total Veterans (*82* Veterans per treatment arm) chosen for this study assumes an ICC of 0.03,[72] cluster size of 8 Veterans per randomized group, 90% power, a two-sided α of 0.05, and a 6-month attrition rate of 25%, similar to MOVE!+UP pilot's overall retention and a meta-analysis of missing data in weight loss RCTs.[73] We will also be powered to detect a meaningful change in PTSD symptoms in the intervention condition at six months, aligned with the average PTSD symptom reduction of 18 (SD=12) observed among *MOVE!*+UP pilot cohort 5 participants and assuming a 9-point clinically meaningful reduction[53] in the control condition.

Table 7. Sample size considerations for *MOVE!*+UP hybrid type 1 trial

ICC (ρ)	Cluster size (m)	Total Sample Size
0.05	10	196
0.05	8	182
0.03	10	172
**0.03**	**8**	**164**
0.01	10	148
0.01	8	144

Note: assumes a two-sided α 0.05, 90% power, a 12 – 3.6 = 8.4 lb difference in weight loss between groups, a weight change SD of 13, a 0.97 within-person correlation, and 25% attrition. The bolded row displays the selected parameters for the proposed study.

Quantitative Statistical Analysis Overview: Aims 1 and 2. We will use intent-to-treat analyses to assess differences between intervention and control arms. We will use mixed effects models to account for clustering since participants in the intervention arm in the same enrollment cohort receive group treatment together. We will attempt to reduce attrition and missing data, which can lead to selection bias.

Missing data. In descriptive analyses, we will report summary statistics on proportion of patients with missing data in each of the study arms. We will report overall rates of missing data as well as specific rates for important variables such as study outcomes and baseline adjustment variables. We will provide descriptive statistics summarizing associations between available auxiliary variables and missingness in important model variables.

Methods for handling missing data in the primary analyses will be pre-specified and not influenced by the descriptive analyses described above. Missing data in the primary and secondary analyses will be accounted for through multiple imputation (MI) methods described below. Prior to multiple imputation, we will attempt to fill in missing baseline values of African-American race/ethnicity from the EHR.

Analyses of primary and secondary outcomes will account for missing data in outcome or baseline adjustment variables through Multiple Imputation (MI). We will perform MI using the Multiple Imputation by Chained Equations (MICE) method. MICE is a general purpose MI method that can handle missingness in variables of mixed data types (e.g. continuous and categorical) as well as

clustered data. Missing values will be imputed separately for each faux treatment group.[1] MI will be performed using the outcome and baseline variables pre-specified for adjustment, and pre-specified auxiliary variables that are either likely associated with missingness and/or the outcome (gender, age, race, employment status, educational attainment, medication usage associated with a) substantial weight loss or b) substantial weight gain). We will create 50 imputed datasets, which will then be analyzed and combined according to Rubin's rules for multiple imputation.

MICE requires specifying a statistical model to generate predictions for each variable with missingness. Table 9 lists the statistical models for each variable that will be used in the imputation procedure. Final imputed values will be based on the predictive mean matching approach, which can provide more plausible values when the distribution of a variable exhibits features such as bimodality or skewness.

Imputation Models

Variable Name	Variable Type	Statistical Model for MICE procedure	Rationale for inclusion
6-month Weight	Continuous	Linear regression	Outcome being imputed
Baseline Weight	Continuous	Linear regression	Typically associated with outcome
African-American race/ethnicity	Binary	Logistic regression	Typically associated with outcome and with missingness in a prior WL trial among Veterans
Employment status	Categorical	Multinomial regression	Associated with missingness in a prior WL trial among Veterans
Educational achievement	Categorical	Multinomial regression	Associated with missingness in a prior WL trial

[1] Citation: https://www.ncbi.nlm.nih.gov/pmc/articles/PMC6408317/

			among Veterans
Age	Continuous	Linear regression	Associated with missingness in a prior WL trial among Veterans
Gender	Categorical	Multinomial regression	Typically associated with outcome and was associated with missingness in a prior WL trial among Veterans
Weight management medication usage in the 6 mos following enrollment (Yes vs. no)	Binary	Logistic regression	Typically associated with outcome
Weight gain meds in the 6 mos following enrollment (Yes vs. no)	Binary	Logistic regression	Typically associated with outcome

References: Prior WL trial among Veterans: https://jamanetwork.com/journals/jama/fullarticle/2799407; Kate will add references for gender, Af-Am race

MICE requires specifying a statistical model to generate predictions for each variable with missingness. Final imputed values will be based on the predictive mean matching approach, which can provide more plausible values when the distribution of a variable exhibits features such as bimodality or skewness.

As suggested by Althouse, in addition to reporting statistical significance based on an alpha of .05, we will report effect sizes and confidence intervals so readers can determine whether the effects warrant policy and clinical practice change.[91] Also recommended by Althouse, although this study includes a single primary outcome for determining effectiveness, therefore reducing likelihood of Type I error, we will also report the actual p-value for all analyses, which would allow a reader to adjust for multiple comparisons.[91] We will report summary statistics for baseline covariates and outcomes (means, standard deviations, and quartiles for continuous variables; frequencies and percentages for binary variables) by randomized group, including the proportion achieving clinically meaningful weight loss (≥ 5% baseline weight).[18]

Statistical Analysis—Specific Aim 1 Primary Outcome.
We will test the primary hypothesis that among Veterans with overweight or obesity, MOVE!+UP will result in significantly greater weight loss using linear mixed effects models. Because this is an RCT, no imbalance across conditions is anticipated. To increase precision of outcome effect estimates, the analysis will adjust for baseline weight and an indicator of self-reported African-American status. Participants in the same enrollment cohort who are assigned to the same treatment arm receive group treatments together, so their outcomes are correlated. To account for this, we will include a random intercept for group treatment cluster in the model. The primary analysis will be based on the following model:

$$Y_{ij}^{(6)} - Y_{ij}^{(0)} = \beta_0 + \beta_1 Intervention_i + \beta_2 Y_{ij}^{(0)} + \beta_4 x_i + \tau_j + \varepsilon_{ij}$$

Where:

- $Y_{ij}^{(6)} - Y_{ij}^{(0)}$ is the *difference in weight at 6 months* $\left(Y_{ij}^{(6)}\right)$ and baseline ($Y_{ij}^{(0)}$¿for subject *i* in group treatment cluster *j*;
- $Intervention_i$ is an indicator of assignment to the intervention MOVE!+UP;
- $Y_{ij}^{(0)}$is baseline weight;
- x_i are baseline covariates; and
- τ_j is the random intercept for group treatment cluster *j*.

Our main goal is to test whether $\beta_1 = 0$, which is the difference in weight change between intervention and control groups. A strength of our approach includes accounting for correlated responses among intervention participants in the same treatment cluster. The approach we chose assumes a homogenous variance between the clusters (cohorts); a recent study showed this may be acceptable in scenarios where ICCs are low, as assumed in our study. Our approach of adjusting for baseline weight and pre-specified baseline covariates will provide the most efficient way of estimating the effect of intervention.

We will calculate a point estimate and 95% confidence interval for the effect of intervention, β_1 , based on the fitted mixed effects model. A test of the null hypothesis of no intervention effect, $\beta_1 = 0$, will be conducted using a significance threshold of 0.05. The test will be based on a t-statistic with denominator degrees of freedom calculated using the Kenward-Rogers approximation. Since we are only testing a single hypothesis for the primary analysis, no adjustment for multiplicity will be needed.

Aim 1 Secondary and Aim 2 Exploratory Analyses

We will compare intervention and control conditions on 6-month PTSD symptom reduction, 12-month weight loss and PTSD symptom reduction, and 6-month change from baseline for other treatment targets. The primary outcome model will be used for continuous outcomes. Note that

only the weight models will include African-American race as a covariate. Binge eating disorder at 6 months will be analyzed using a random intercept logistic regression model with an indicator variable for intervention arm. We will use the same statistical test as described for the primary analysis. Given analyses are exploratory, no adjustment for multiple comparisons will be made.

Note: Aim 3 analyses are not covered in this statistical analysis plan.

Table 1. Statistical Models[2]

Analysis	Outcome(s)	Independent Variables
Primary analysis	6-month absolute weight change from baseline (pounds)	Binary treatment indicator<br><br>Baseline weight<br><br>Binary African-American race indicator
Secondary analyses	1. 6-month absolute change in PTSD symptoms from baseline<br>2. 12-month absolute change in PTSD symptoms from baseline<br>3. 12-month absolute weight change from baseline (pounds)	Binary treatment indicator<br><br>Baseline outcome<br><br>Binary African-American indicator (weight analysis only)
Exploratory analyses	6-month absolute change from baseline in:<br><br>1. Diet quality<br>2. Eating habits<br>3. Night eating<br>4. Emotional eating<br>5. Average MVPA and average LMVPA (adjust for total # of valid days at baseline and 6 months)<br>6. Social support – Diet<br>7. Social discouragement – Diet<br>8. Social support – physical activity<br>9. Social discouragement – physical activity<br>10. Insomnia Severity Index (ISI)<br>11. Depression symptoms<br>12. Internalized Weight Bias	Binary treatment indicator<br><br>Baseline outcome

[2] All models account for clustering through the inclusion of a random intercept for group treatment cluster.

Analysis	Outcome(s)	Independent Variables
Exploratory Analyses	Binge eating at 6 months	Binary treatment indicator
Sensitivity analyses:<br><br>adjusting for prior weight loss treatment	1. 6-month absolute weight change from baseline (pounds)<br>2. 12-month absolute weight change from baseline (pounds)	Binary treatment indicator<br><br>Baseline outcome<br><br>Binary African-American indicator<br><br>Total number of MOVE! visits 12 months prior to baseline<br><br>Binary indicator of prescription weight loss medications at baseline
Sensitivity analyses:<br><br>adjusting for prior mental health treatment	1. 6-month absolute change in PTSD symptoms from baseline<br>2. 12-month absolute change in PTSD symptoms from baseline	Binary treatment indicator<br><br>Baseline outcome<br><br>Total number of mental health visits 12 months prior to baseline, per EHR<br><br>Number of PTSD visits in the prior year<br><br>Binary indicator of PTSD medications in the last year
Sensitivity analyses:<br><br>Including auxiliary variables to assess impact of missing data	1. 6-month absolute weight change from baseline (pounds)<br>2.	Binary treatment indicator<br><br>Baseline outcome<br><br>Binary African-American indicator (weight analyses only)<br><br>Auxiliary variables discussed in the Handling of Missing Data section. Plus those that appeared possibly associated with missingness on 6-month weight in descriptive comparisons.
Sensitivity: bariatric surgery	1. 6-month absolute weight change from baseline (pounds)<br>2. 12-month absolute weight change	Binary treatment indicator

Analysis	Outcome(s)	Independent Variables
influence	from baseline (pounds)	Baseline outcome<br><br>Binary African-American indicator (weight analyses only)<br><br>Remove veteran outcome with bariatric surgery. However no one had bariatric surgery during the study period so this analysis is not being conducted.
Sensitivity analysis: examine differences in utilization across tx conditions and the influence of these factors on weight loss outcome.	1. 6-month absolute weight change from baseline (pounds)<br>2. 12-month absolute weight change from baseline (pounds)<br>3.	Binary treatment indicator<br><br>Baseline outcome<br><br>Binary African-American indicator (weight analyses only)<br><br>-Binary indicator of prescription weight loss medications from baseline to 6 and 12 months reported in EHR and/or self report<br><br>-binary indicator of non-MOVE! +UP/MOVE! weight management care (i.e., any self-reported non-Va program, dietician visits TeleMOVE!). Will not include self-reported use of websites, Fitbits etc.<br><br>If the pattern of results changes from what was found in the primary analysis, we would pursue a formal mediation analysis.
Sensitivity analysis: If we see substantial issues with the q-q plots when the primary analysis is	1. 6-month log-transformed weight change from baseline (pounds)<br>1.	Binary treatment indicator<br><br>Baseline outcome<br><br>Binary African-American indicator (weight analyses only

Analysis	Outcome(s)	Independent Variables
run, we will do a sensitivity analysis with a log-transformed outcome		

Qualitative Data Coding and Analysis—Aim 3. If we have a positive trial, we will use template analysis, a method for thematic coding of text well suited when the aim is to investigate themes with a clear structure.[105] The CFIR will guide coding and analysis of qualitative interview transcripts, field notes, and suggestions on Veteran satisfaction measures, using CFIR coding guidelines.[95,103] Coding discrepancies will be resolved by a consensus approach with input from our co-investigators. After codes have been applied to each data source, memos will be written with summary statements and corresponding supporting quotes. Findings will be summarized in products and will guide adaptations to *MOVE!*+UP content and procedures, and inform future implementation activities.

5.7 Withdrawal of Subjects

a. Veteran participants (Aims 1 and 2)
Participants were reminded that they are free to withdraw from the study at any time without loss of any materials they have obtained through the study and that their information will be kept confidential. If an immediate and serious risk is discovered, staff will consult with the PI, Dr. Hoerster.

If the data and safety monitoring committee (DSMC) member(s) determine that involvement in the study was not in a Veteran's best interest, we would withdraw them from the study. Although we did not identify hard-and-fast stopping rules because we wanted to make Veteran involvement as individualized as possible, we would likely withdraw a participant from the study if they expressed suicidal intent or had an extreme exacerbation of PTSD symptoms (i.e., an increase of 10 points on the PTSD Checklist in the course of a week) that they and/or PI or DSMC attributed to study participation. Any adverse effect of moderate or greater severity deemed to be definitely associated with the study will be reported to the participant and his/her health care provider(s). Serious adverse events (SAEs) will be reported to the IRB following standard IRB requirements. We will consult the DSMC to assess the impact of significant data loss due to problems in recruitment, retention or data collection.

Should something come up that may affect the subject’s health or welfare (for example a subject becomes pregnant or plans bariatric surgery during the study)

we would require that subject speak with their primary care provider or other physician to confirm that it is okay for them to continue participating in the study.

If a participant decided to withdraw, or if they were terminated from the study, a person from the study team may need to meet with them to discuss the necessary steps that they may need to take to end Veteran participation in the study. Veterans were also reminded that If they decided to withdraw from the study, no new information would be collected from them (aside from prospective CDW data, noted in the consent as described above); however, data already collected will continue to be part of the analyses.

b. VA Employees (Aim 3)
VA staff participants will be assured that they can, at any time, refuse to answer any question, and at any time elect to withdraw from the study. They also will be assured that their participation or lack thereof will not be reported to their supervisor or peers nor will it have any impact on their work environment and that they are free to participate in the presence of union representation.

5.8 Union Notification

Of note, during the pilot, we notified the union of our research and recruitment of VA employees (Aim 3) for the purposes of that (essentially identical) study, and the Union Representative reviewed our interview guide and determined that as our study does not concern employees' conditions of employment, union notification is not required (see submitted Union Approval).

Regarding our Veteran participants (Aim 1 and 2), we likely won't know if a Veteran is also an employee until we open up their medical chart in CPRS. However, we confirmed eligibility (diagnoses and participation in care) in the Veteran's medical record in CPRS during the recruitment and screening process. Furthermore, because study participation may involve weekly intervention sessions, any Veteran employees who might be interested in participating would also need to participate on their own time (that is, during their off hours), so we would likely be able to ascertain if an interested participant is also a Veteran employee fairly early on. Regardless, because we do not explicitly discuss conditions of Veteran employees' employment in any of our questionnaires or interviews, we felt we did not need to pursue union notification/approval as we do not meet that criteria (given to us by the union representative as noted above) for union review.

6.0 Reporting

Safety Assessment. Opportunities for participants to systematically report safety data occured during the baseline assessment visit, 6-month assessment visit, and the 12-month assessment visit (see attached Medical History AE Assessment). Specifically, study staff administered a medical history AE assessment at the 6- and 12-month visits. Safety information also was collected when reported in clinical or other encounters with members of the study team (e.g., interventionists, MOVE! clinicians, and/or research staff). Dr. Hoerster and Dr. Donovan (DSMC

member) independently assesed each AE reported on the Medical History AE form or to study team members for expectedness in the study population, severity, start and resolution times and relation to study activity. They also w identified any important follow-up activities to ensure appropriate care is received. With respect to actions that need to be taken on SAEs, Dr. Donovan in consultation with the DSMC, would make recommendations regarding the following: consideration of early termination of the study due to treatment safety concerns, and modification of the study protocol regarding areas of recruitment, participant retention, outcome assessment, statistical analysis, or general trial operations. For AEs reported during the intervention phase, they were monitored on at least a weekly basis until deemed to have been resolved. If they were ongoing after the 16-session intervention phase, they were reassessed on a weekly basis for participants whose care team wass not yet engaged; once the care team is engaged in managing the issue, the AE wouldl only be reassessed at the 6-month (and 12-month if needed) assessment timeframe until it is resolved. No assessment of AEs will continue beyond the 12-month assessment timeframe unless deemed necessary by the DSMC.

Development or exacerbation of the following conditions or states are expected AEs in participants who have a high BMI and PTSD and adopt healthy eating and physical activity program:

- Gastrointestinal symptoms related to diet;
- Musculoskeletal symptoms or injury resulting from physical activity;
- Weight associated disorders including diabetes, hypertension, liver disease, cardiovascular disease, asthma, COPD, clotting problems, and other lung related conditions;
- Other conditions associated with unhealthy health behaviors, such as from tobacco and alcohol disorders (e.g. cancer);
- Age-related illnesses;
- Mental health symptom exacerbations;
- COVID-19 (SARS-CoV-2) infection due to population prevalence;
- Unhealthy eating behaviors (e.g., binge eating)

Study progress and safety are reviewed in weekly staff meetings (and more frequently if needed). The frequency and type of data review for this study differs according to the type of data and can be summarized in Table 8 below.

Table 8. Data Review and Monitoring

Data type	Frequency of review	Reviewer
Subject accrual (adherence to protocol regarding demographics, inclusion/exclusion)	Daily in peak recruitment phases; monthly otherwise	Principal Investigator (PI) and research coordinator (RC)
Adverse event rates	Weekly	PI and RC, and 2nd DSMC AE rater
Compliance to treatment	Weekly	PI and RC
Data quality	Weekly	PI and RC

Data and safety monitoring committee (DSMC) reports	Quarterly for chair and annually for full committee, with additional consultation as needed	PI, RC, and DSMC

Data and Safety Monitoring Committee. Upon discovering an SAE, study staff wouldprovide the IRB and DSMC with a report describing the duration (start and stop dates and times), severity, outcome, and relation to study activity, according to the required timelines. The DSMC would potentially request additional information if it deems additional deliberation is warranted. Serious problems will also be reported within 5 days to the IRB.

For all other events, staff summarized and reported them to the DSMC the numbers and types of all AEs (not by treatment arm) on a quarterly basis to the chair and full committee annually. At their discretion, the DSMC could y request unblinded results in order to determine the nature and extent of effect of the intervention. Were the DSMC to make this request, we will maintain blinding of the investigators and the staff involved in follow-up data collection and analysis. If, at any time, the investigators believe they are seeing an increase in SAEs that is a cause of concern, they would bring this to the attention of the DSMC.

At the annual meeting, the DSMC reviewed AE/SAEs and the quality of data, as well as study progress (and provided o recommendations, as appropriate, with respect to):

- Determination of any actions to be taken in response to SAEs;
- Reports related to study operations and the quality of the data;
- Consideration of early termination of the study because of treatment safety concerns;
- Modifications in the study protocol concerning recruitment, participant retention, data quality, outcome assessment, statistical analysis, or general trial operations.

7.0 Privacy and Confidentiality

Protected health information (PHI) is obtained during the course of this study. Every effort is made to reduce risks to Veteran and VA employee participants. A number of steps were taken to ensure confidentiality and data protection throughout the study, with additional detail in **Section 9.0 Information Security and Data Storage/Movement**, below. All study staff complete the appropriate human subjects research trainings including VA Human Subjects Protection, VA Privacy and Information Security Awareness and Rules of Behavior, and Privacy and HIPAA Training. Clinical notes were written for Veterans when they participate in MOVE! +UP or MOVE! because those are part of standard clinical care. Standard VA clinical documentation practices were used to ensure protection of privacy and confidentiality. Special

measures were taken with all digitally-recorded interviews and sessions to ensure confidentiality of Veteran and VA staff participant information. Digital recordings, and subsequent transcriptions and rating sheets, contain no identifying information, but will contain only a unique study ID assigned to the participant. The digital recorder remains securely in the research staff's possession at all times, and staff will securely transfer the digitally recorded interview data to a secure, project-specific, folder (SFFX folder) on the protected server on a drive specifically designed to house and protect research data. After downloading the digital recording to the secure drive, the interviewer deletes the data entirely from the digital recorder before transporting it. In this way, the only source of digital recordings will be on the secure server. To record VVC sessions for *MOVE!*+UP fidelity assessment, we have found that the most secure and efficient way to record the sessions is to use the VA-approved software Audacity, allowing us to store a recording of the session only directly into the secure research folder.

As a result of these measures, we do not expect any breach of confidentiality for Veteran and VA staff participants. Participation is voluntary, and Veteran and VA staff participants could withdraw from the study at any time and are free to skip assessment questions. Unique risk protection considerations for Veterans and employees are discussed in a and b below.

<u>a. Veteran participants (Aims 1 and 2)</u>
Participants bear no financial risk from any adverse effects of encounters from this study. Primary Care Provider (or other VA physician) approval was obtained before enrollment into the intervention or control condition of the study, and participants were engaged in PTSD care, which facilitated care coordination for medical and mental health issues that arose. For recruitment mailings to prospective participants, they were made via an introductory letter with a stamped postcard allowing them to opt out of further contact by the study team thus limiting risk for invasion of privacy. Participants completed assessment measures via VVC <u>(or other approved video conferencing modality should VVC not be working)</u> or over the phone. However, accelerometers were collected via mail in the pre-packaged envelope that only includes their subject ID and no identifiers. Study staff reminded the participants to remove the accelerometer and arranged for UPS to pick up the accelerometer return envelope directly from the participant or for them to be returned by mail.

The safety procedures for the MOVE!+UP community walk are detailed earlier, including the pre-walk audit for a safe route.

<u>b. VA Employees (Aim 3)</u>
ll interviews with employees will be conducted via Webex for Business, the method now encouraged by VA. Interviews will be transcribed, with individually identifiable information redacted and stored separately from unique subject identifiers. Aside from for recruitment purposes, no identifying information will be needed for research involving VA staff. We will ensure that all VA staff participants understand they will be assigned a unique study ID that will be used to label all study data such that data could not be linked to individuals.

8.0 Project Management and Communication Plan

8.1 Project Management

Dr. Hoerster oversees all aspects of the study. To ensure all elements of the study protocol are followed and that study goals are met, the PI conducted regular meetings with project staff to review study procedures and status, barriers encountered, and develop responses to any identified issues.The experienced research coordinator coordinated recruitment, retention, and assessment; manage blinded research assistants She will conduct qualitative interviews and analysis with oversight from the PI if the trial is positive. To protect the blind and equipoise, research assistants blinded to study condition and hypotheses conducted assessments. Dr. Hoerster train and provided weekly clinical supervision to the Veteran peer support and psychologist facilitators, based on fidelity assessments of audio-recorded sessions. A data provisioner pulled administrative data to be used in recruitment, and will pull additional variables to be used as covariates in Aim 1 analyses, and in Aim 3 analyses. The study biostatisticians will run analyses for Aims 1 and 2, and quantitative Aim 3 analyses. The PI will provide guidance, and if relevant, Dr. Wong will oversee cost and utilization analyses.

We modified an Operations Manual developed for *MOVE!*+UP's pilot. Dr. Hoerster coordinates communication among all study staff and Co-Is. Weekly staff meetings were convened to ensure all study targets are met in a timely fashion and to address issues. All Co-Is meet in-person or by phone/video conference at least quarterly. The research coordinator distributes minutes to all staff and Co-Is following weekly meetings to ensure reliable communication of key study management issues. We presented to our COIN Veteran Engagement Board (VEB) to obtain input prior to the RCT and will again regarding implementation and dissemination, and as needed throughout.

8.2 Communication Plan

Information about the communication of study progress and safety is discussed in detail in **Section** above.

9.0 Information Security and Data Storage/Movement

All data will are stored in offices at VA Puget Sound HSR&D with locked filing cabinets and password-protected computers. The administrative data extracted for recruitment is stored and accessed within the VINCI environment as well as within the PI's secure VA research data folder. The investigators, project coordinator, and research assistants are the only staff to have access to confidential records. Following assessments, we will transport completed questionnaires in a locking medical bag to transport for data entry and secure storage.

ActiGraph accelerometer data will be processed and downloaded using the ActiLife software available on a non-VA network laptop (i.e., one purchased through Seattle Institute For Biomedical And Clinical Research (SIBCR) funds for this specific use for the study) as we and the IT support staff who have attempted to help us, are currently unable to download the software onto the VA network because of Federal Information Processing Standards (FIPS) incompatibility. However, the ActiLife program is still approved for use per VA TRM. All downloaded accelerometer data will then be transferred using a VA-approved external hard drive and saved to the PI's secure research data folder on the VA network. All data will be deleted from the laptop and the external hard drive as soon as we are able to confirm that the accelerometer data have transferred to the secure network, which will require having ActiLife software installed. As such, we are storing the deidentified (subject ID only) accelerometer data on the laptop until we are able to install ActiLife on the VA network since we will need that software for processing and analyzing the accelerometer data. The laptop and external hard drive will be locked in a file cabinet in the PI's HSR&D closed door office. Access to the laptop is also password protected, with only select IRB-approved study staff accessing it.

REDCap (a VA-approved, secure Web application that facilitates the collection and entry of research data) is used to maintain non-PHI study data (i.e., screening information, study attempts for contact, etc.). Only approved and trained study staff have access to the REDCap project database. No identifiers are stored in the database, and all information is linked via a unique subject ID. This system replaced our prior study database because it is a more user-friendly interface for data entry by multiple users and to facilitate study management. There will be no Veteran-facing REDCap interface (e.g., for questionnaire data collection).

ID numbers rather than names will be used whenever possible. Lists of Study ID numbers will be generated randomly using software prior to enrolling subjects in either the RCT or the employee interviews and ID numbers will be assigned in the order in which they were generated. A single master list ("crosswalk") contains subject information, as well as participants' unique Study ID. The crosswalks are stored in a password-protected file in a password-protected computer, in a restricted file on the VA network (in secure SFFX folder) separate at all times from the study data. Study data will never contain any identifying information. Aggregate data will be presented to external audiences. Individual identifiers will be deleted when they are no longer necessary for the project. Data will be maintained and destroyed in accordance with the most current VA Records Control Schedule. Additional information about storage of recordings for fidelity assessment is in **Section 7.0 Privacy and Confidentiality** above.

10.0 References

1.Dedert EA, Calhoun PS, et al. Posttraumatic stress disorder, cardiovascular, and metabolic disease: a review of the evidence. *Ann Behav Med.* 2010;39:61-78.
2.Kilbourne AM, Ignacio RV, et al. Datapoints: are VA patients with serious mental illness dying younger? *Psychiatr Serv.* 2009;60:589.
3.Schnurr PP, Lunney CA, et al. Posttraumatic stress disorder and quality of life: extension of findings to veterans of the wars in Iraq and Afghanistan. *Clin Psychol Rev.* 2009;29:727-35.
4.Danaei G, Ding EL, et al. The preventable causes of death in the United States: comparative risk assessment of dietary, lifestyle, and metabolic risk factors. *PLoS Med.* 2009;6:e1000058.
5.Strohle A. Physical activity, exercise, depression and anxiety disorders. *J Neural Transm.* 2009;116:777-84.
6.Walsh R. Lifestyle and mental health. *Am Psychol.* 2011;66:579-92.
7.Goldstein LA, Mehling WE, et al. Veterans Group Exercise: A randomized pilot trial of an Integrative Exercise program for veterans with posttraumatic stress. *Journal of affective disorders.* 2018;227:345-52.
8.Hall KS, Gregg J, et al. Physical Activity Counseling Promotes Physical and Psychological Resilience in Older Veterans with Posttraumatic Stress Disorder. *Ment Health Phys Act.* 2016;11:53-59.
9.LeardMann CA, Kelton ML, et al. Prospectively assessed posttraumatic stress disorder and associated physical activity. *Public Health Rep.* 2011;126:371-83.
10.Johannessen KB, Berntsen D. Losing the symptoms: weight loss and decrease in posttraumatic stress disorder symptoms. *J Clin Psychol.* 2013;69:655-60.
11.U.S. Department of Veterans Affairs, U.S. Department of Defense. VA/DoD Clinical Practice Guideline for the Management of Posttraumatic Stress Disorder and Acute Stress Disorder. 2017; https://www.healthquality.va.gov/guidelines/MH/ptsd/VADoDPTSDCPGFinal012418.pdf. Accessed 5/1/19.
12.Steenkamp MM, Litz BT, et al. Psychotherapy for Military-Related PTSD: A Review of Randomized Clinical Trials. *JAMA.* 2015;314:489-500.
13.Shiner B, Westgate CL, et al. A Retrospective Comparative Effectiveness Study of Medications for Posttraumatic Stress Disorder in Routine Practice. *J Clin Psychiatry.* 2018;79.
14.Shalev A, Liberzon I, et al. Post-Traumatic Stress Disorder. *N Engl J Med.* 2017;376:2459-69.
15.Chan D, Cheadle AD, et al. Health care utilization and its costs for depressed veterans with and without comorbid PTSD symptoms. *Psychiatr Serv.* 2009;60:1612-7.
16.Le QA, Doctor JN, et al. Cost-effectiveness of prolonged exposure therapy versus pharmacotherapy and treatment choice in posttraumatic stress disorder (the Optimizing PTSD Treatment Trial): a doubly randomized preference trial. *J Clin Psychiatry.* 2014;75:222-30.
17.Kahwati LC, Lance TX, et al. RE-AIM evaluation of the Veterans Health Administration's MOVE! Weight Management Program. *Translational Behavioral Medicine.* 2011;1:551-60.
18.Jensen MD, Ryan DH, et al. 2013 AHA/ACC/TOS guideline for the management of overweight and obesity in adults: a report of the American College of Cardiology/American Heart Association Task Force on Practice Guidelines and The Obesity Society. *Circulation.* 2014;129:S102-38.
19.Hoerster KD, Lai Z, et al. Weight Loss After Participation in a National VA Weight Management Program Among Veterans With or Without PTSD. *Psychiatr Serv.* 2014;65:1385-88.

20.Klingaman EA, Hoerster KD, et al. Veterans with PTSD report more weight loss barriers than Veterans with no mental health disorders. *Gen Hosp Psychiatry.* 2016;39:1-7.
21.Hall KS, Hoerster KD, et al. Post-traumatic stress disorder, physical activity, and eating behaviors. *Epidemiologic Reviews.* 2015;37:103-15.
22.Hoerster KD, Jakupcak M, et al. PTSD and depression symptoms are associated with binge eating among US Iraq and Afghanistan veterans. *Eating Behaviors.* 2015;17:115-8.
23.Hoerster KC, Afari N, et al. Mental health considerations for optimizing behavioral weight management interventions with obese Veterans (symposium). 35th Annual Meeting of the Society of Behavioral Medicine; 2014;*Annals of Behavioral Medicine*;47:s145. Philadelphia, PA.
24.Hoerster K, Gray K, et al. Predictors of diet quality and physical activity improvements in a weight loss program for overweight Veterans with PTSD. poster presented at 39th Annual Meeting of the Society of Behavioral Medicine; 2018;*Annals of Behavioral Medicine*;52:S79. New Orleans, LA.
25.Hoerster K, Nelson K, et al. Developing a peer support weight loss program: a model for iterative refinement and partnered research. paper presented at 38th annual meeting of the Society of Behavioral Medicine; 2017;*Annals of Behavioral Medicine*; 51:s689. San Diego, CA.
26.Rutter LA, Weatheril RP, et al. Posttraumatic Stress Disorder Symptoms, Depressive Symptoms, Exercise, and Health in College Students. *Psychol Trauma.* 2013;5:56-61.
27.Chinman M, Henze K, et al. Peer Specialist Toolkit: Implementing Peer Support Services in VHA. 2012; https://www.mirecc.va.gov/visn4/docs/Peer_Specialist_Toolkit_FINAL.pdf. Accessed September 13, 2013.
28.Jain S, McLean C, et al. Does the integration of peers into the treatment of adults with posttraumatic stress disorder improve access to mental health care? A literature review and conceptual model. *J Trauma Stress Disor Treat* 2013;2:3.
29.Watts BV, Schnurr PP, et al. Meta-analysis of the efficacy of treatments for posttraumatic stress disorder. *J Clin Psychiatry.* 2013;74:e541-50.
30.Centers for Disease Control and Prevention. How much physical activity do adults need? 2014; http://www.cdc.gov/physicalactivity/everyone/guidelines/adults.html. Accessed October 12, 2014.
31.Curran GM, Bauer M, et al. Effectiveness-implementation hybrid designs: combining elements of clinical effectiveness and implementation research to enhance public health impact. *Med Care.* 2012;50:217-26.
32.U.S. Department of Veterans Affairs and Department of Defense. VA/DOD Clinical Practice Guideline for Screening and Management of Overweight and Obesity. 2014; https://www.healthquality.va.gov/guidelines/cd/obesity/. Accessed April 1, 2018.
33.Dorflinger LM, Masheb RM. PTSD is associated with emotional eating among veterans seeking treatment for overweight/obesity. *Eating behaviors.* 2018;31:8-11.
34.Talbot LS, Maguen S, et al. Posttraumatic stress disorder is associated with emotional eating. *J Trauma Stress.* 2013;26:521-5.
35.Dorflinger LM, Ruser CB, et al. Night eating among veterans with obesity. *Appetite.* 2017;117:330-34.
36.Kinsinger LS, Jones KR, et al. Design and dissemination of the MOVE! Weight-Management Program for Veterans. *Prev Chronic Dis.* 2009;6:A98.
37.Samdal GB, Eide GE, et al. Effective behaviour change techniques for physical activity and healthy eating in overweight and obese adults; systematic review and meta-regression analyses. *Int J Behav Nutr Phys Act.* 2017;14:42.
38.Baron KG, Reid KJ, et al. Exercise to improve sleep in insomnia: exploration of the bidirectional effects. *Journal of clinical sleep medicine : JCSM : official publication of the American Academy of Sleep Medicine.* 2013;9:819-24.

39.Boutcher SH, Dunn SL. Factors that may impede the weight loss response to exercise-based interventions. *Obes Rev.* 2009;10:671-80.
40.St-Onge MP. The role of sleep duration in the regulation of energy balance: effects on energy intakes and expenditure. *Journal of clinical sleep medicine.* 2013;9:73-80.
41.Brug J. Determinants of healthy eating: motivation, abilities and environmental opportunities. *Fam Pract.* 2008;25 Suppl 1:i50-5.
42.Sherwood NE, Jeffery RW. The behavioral determinants of exercise: implications for physical activity interventions. *Annu Rev Nutr.* 2000;20:21-44.
43.Hoerster KD, Campbell SB, et al. PTSD is associated with Poor Health Behavior and Greater Body Mass Index through Depression, increasing Cardiovascular Disease and Diabetes Risk among U.S. Veterans. *Preventive Medicine Reports.* in press.
44.Ma J, Rosas LG, et al. Effect of Integrated Behavioral Weight Loss Treatment and Problem-Solving Therapy on Body Mass Index and Depressive Symptoms Among Patients With Obesity and Depression: The RAINBOW Randomized Clinical Trial. *JAMA.* 2019;321:869-79.
45.Naslund JA, Whiteman KL, et al. Lifestyle interventions for weight loss among overweight and obese adults with serious mental illness: A systematic review and meta-analysis. *Gen Hosp Psychiatry.* 2017;47:83-102.
46.Watkins LE, Sprang KR, et al. Treating PTSD: A Review of Evidence-Based Psychotherapy Interventions. *Front Behav Neurosci.* 2018;12:258.
47.Abramowitz JS. The practice of exposure therapy: relevance of cognitive-behavioral theory and extinction theory. *Behav Ther.* 2013;44:548-58.
48.Leahey TM, Wing RR. A Randomized Controlled Pilot Study Testing Three Types of Health Coaches for Obesity Treatment: Professional, Peer, and Mentor. *Obesity.* 2013;21:928-34.
49.Chinman M, Oberman RS, et al. A Cluster Randomized Trial of Adding Peer Specialists to Intensive Case Management Teams in the Veterans Health Administration. *The journal of behavioral health services & research.* 2015;42:109-21.
50.Webel AR, Okonsky J, et al. A systematic review of the effectiveness of peer-based interventions on health-related behaviors in adults. *Am J Public Health.* 2010;100:247-53.
51.U.S. Congress. VA MISSION Act of 2018. 2018; https://www.congress.gov/115/bills/s2372/BILLS-115s2372enr.pdf. Accessed May 15, 2019.
52.Spring B, Sohn MW, et al. Individual, facility, and program factors affecting retention in a national weight management program. *BMC Public Health.* 2014;14:363.
53.Stefanovics EA, Rosenheck RA, et al. Minimal Clinically Important Differences (MCID) in Assessing Outcomes of Post-Traumatic Stress Disorder. *Psychiatr Q.* 2018;89:141-55.
54.Eftekhari A, Ruzek JI, et al. Effectiveness of national implementation of prolonged exposure therapy in Veterans Affairs care. *JAMA Psychiatry.* 2013;70:949-55.
55.Morin CM, Belleville G, et al. The Insomnia Severity Index: psychometric indicators to detect insomnia cases and evaluate treatment response. *Sleep.* 2011;34:601-8.
56.Masheb RM, Chan SH, et al. State of the art conference on weight management in VA: Policy and research recommendations for advancing behavioral interventions. *J Gen Intern Med.* 2017;32:74-78.
57.U.S. Department of Veterans Affairs. HSR&D Priority Domains. 2019; https://www.hsrd.research.va.gov/funding/PriorityDomains2019.pdf. Accessed May 1, 2018.
58.Pietrzak RH, Goldstein RB, et al. Prevalence and Axis I comorbidity of full and partial posttraumatic stress disorder in the United States: results from Wave 2 of the National Epidemiologic Survey on Alcohol and Related Conditions. *J Anxiety Disord.* 2011;25:456-65.
59.Satariano WA, Guralnik JM, et al. Mobility and aging: new directions for public health action. *Am J Public Health.* 2012;102:1508-15.
60.American College of O, Gynecologists. ACOG Committee opinion no. 548: weight gain during pregnancy. *Obstet Gynecol.* 2013;121:210-2.

61.Bovin MJ, Marx BP, et al. Psychometric properties of the PTSD Checklist for Diagnostic and Statistical Manual of Mental Disorders-Fifth Edition (PCL-5) in veterans. *Psychol Assess.* 2016;28:1379-91.
62.Goldberg JH, Kiernan M. Innovative techniques to address retention in a behavioral weight-loss trial. *Health Educ Res.* 2005;20:439-47.
63.Dannenberg AL, Cramer TW, et al. Assessing the walkability of the workplace: a new audit tool. *Am J Health Promot.* 2005;20:39-44.
64.Goodrich DE, Larkin AR, et al. Adverse events among high-risk participants in a home-based walking study: a descriptive study. *Int J Behav Nutr Phys Act.* 2007;4:20.
65.Resnick B, Bellg AJ, et al. Examples of implementation and evaluation of treatment fidelity in the BCC studies: where we are and where we need to go. *Ann Behav Med.* 2005;29 Suppl:46-54.
66.Borrelli B. The assessment, monitoring, and enhancement of treatment fidelity in public health clinical trials. *Journal of public health dentistry.* 2011;71 Suppl 1:S52-63.
67.Kearney LK, Post EP, et al. The role of mental and behavioral health in the application of the patient-centered medical home in the Department of Veterans Affairs. *Transl Behav Med.* 2011;1:624-8.
68.U.S. Department of Veterans Affairs. MOVE! Weight Management Program 2018; https://www.move.va.gov/. Accessed March 15, 2018.
69.Freedland KE, Mohr DC, et al. Usual and unusual care: existing practice control groups in randomized controlled trials of behavioral interventions. *Psychosom Med.* 2011;73:323-35.
70.Friedman L, Furberg C, et al. *Fundamentals of Clinical Trials.* Springer; 2015.
71.Moin T, Damschroder LJ, et al. Diabetes Prevention Program Translation in the Veterans Health Administration. *Am J Prev Med.* 2017;53:70-77.
72.Wing RR, Leahey T, et al. Do weight loss and adherence cluster within behavioral treatment groups? *Obesity (Silver Spring).* 2014;22:638-44.
73.Elobeid MA, Padilla MA, et al. Missing data in randomized clinical trials for weight loss: scope of the problem, state of the field, and performance of statistical methods. *PLoS One.* 2009;4:e6624.
74.Allen DD. Validity and reliability of the movement ability measure: a self-report instrument proposed for assessing movement across diagnoses and ability levels. *Phys Ther.* 2007;87:899-916.
75.Pinto-Carral A, Fernandez-Villa T, et al. Patient-Reported Mobility: A Systematic Review. *Arch Phys Med Rehabil.* 2016;97:1182-94.
76.PhenX Toolkit. National Research Action Plan: PhenX Toolkit (Ver 22.2). 2018; http://www.phenxtoolkit.org Accessed May 15, 2018.
77.Blevins CA, Weathers FW, et al. The Posttraumatic Stress Disorder Checklist for DSM-5 (PCL-5): Development and Initial Psychometric Evaluation. *J Trauma Stress.* 2015;28:489-98.
78.Freedson PS, Miller K. Objective monitoring of physical activity using motion sensors and heart rate. *Res Q Exerc Sport.* 2000;71:S21-9.
79.Hendelman D, Miller K, et al. Validity of accelerometry for the assessment of moderate intensity physical activity in the field. *Med Sci Sports Exerc.* 2000;32:S442-9.
80.Maciejewski ML, Shepherd-Banigan M, et al. Systematic Review of Behavioral Weight Management Program MOVE! for Veterans. *Am J Prev Med.* 2018;54:704-14.
81.Domecq JP, Prutsky G, et al. Clinical review: Drugs commonly associated with weight change: a systematic review and meta-analysis. *J Clin Endocrinol Metab.* 2015;100:363-70.
82.Paxton AE, Strycker LA, et al. Starting the conversation performance of a brief dietary assessment and intervention tool for health professionals. *Am J Prev Med.* 2011;40:67-71.

83.Allison KC, Lundgren JD, et al. The Night Eating Questionnaire (NEQ): psychometric properties of a measure of severity of the Night Eating Syndrome. *Eating behaviors.* 2008;9:62-72.
84.Masheb RM, Grilo CM. Emotional overeating and its associations with eating disorder psychopathology among overweight patients with binge eating disorder. *Int J Eat Disord.* 2006;39:141-6.
85.Kroenke K, Spitzer RL, et al. The PHQ-15: validity of a new measure for evaluating the severity of somatic symptoms. *Psychosom Med.* 2002;64:258-66.
86.Carlson JA, Sallis JF, et al. Brief Physical Activity-Related Psychosocial Measures: Reliability and Construct Validity. *J Phys Act Health.* 2012;9:1178-86.
87.Norman GJ, Carlson JA, et al. Reliability and validity of brief psychosocial measures related to dietary behaviors. *Int J Behav Nutr Phys Act.* 2010;7:56.
88.Bastien CH, Vallieres A, et al. Validation of the Insomnia Severity Index as an outcome measure for insomnia research. *Sleep Med.* 2001;2:297-307.
89.Kroenke K, Spitzer RL, et al. The PHQ-9: validity of a brief depression severity measure. *J Gen Intern Med.* 2001;16:606-13.
90.Schafer J. *Analysis of Incomplete Multivariate Data.* New York, NY: Chapman and Hall; 1997.
91.Althouse AD. Adjust for Multiple Comparisons? It's Not That Simple. *Ann Thorac Surg.* 2016;101:1644-5.
92.Pocock SJ, McMurray JJV, et al. Statistical Controversies in Reporting of Clinical Trials: Part 2 of a 4-Part Series on Statistics for Clinical Trials. *J Am Coll Cardiol.* 2015;66:2648-62.
93.Flight L, Allison A, et al. Recommendations for the analysis of individually randomised controlled trials with clustering in one arm - a case of continuous outcomes. *BMC Med Res Methodol.* 2016;16:165.
94.Zhang S, Paul J, et al. Empirical comparison of four baseline covariate adjustment methods in analysis of continuous outcomes in randomized controlled trials. *Clin Epidemiol.* 2014;6:227-35.
95.Damschroder LJ, Aron DC, et al. Fostering implementation of health services research findings into practice: a consolidated framework for advancing implementation science. *Implement Sci.* 2009;4:50.
96.Lutes LD, Dinatale E, et al. A randomized trial of a small changes approach for weight loss in veterans: design, rationale, and baseline characteristics of the ASPIRE-VA trial. *Contemp Clin Trials.* 2013;34:161-72.
97.Liu CF, Rubenstein LV, et al. Organizational cost of quality improvement for depression care. *Health Serv Res.* 2009;44:225-44.
98.Shaw WD. *Searching for the Opportunity Cost of an Individual's Time.* 1992.
99.Liu CF, Batten A, et al. Fee-for-Service Medicare-Enrolled Elderly Veterans Are Increasingly Voting with Their Feet to Use More VA and Less Medicare, 2003-2014. *Health Serv Res.* 2018;53 Suppl 3:5140-58.
100.Hebert PL, Liu CF, et al. Patient-centered medical home initiative produced modest economic results for Veterans Health Administration, 2010-12. *Health Aff (Millwood).* 2014;33:980-7.
101.Agency for Healthcare Research and Quality. Guide to Prevention Quality Indicators: Hospital Admission for Ambulatory Care Sensitive Conditions. 2002; https://www.ahrq.gov/downloads/pub/ahrqqi/pqiguide.pdf. Accessed Aug 1, 2018.
102.Phibbs CS, Bhandari A, et al. Estimating the costs of VA ambulatory care. *Med Care Res Rev.* 2003;60:54S-73S.
103.Consolidated Framework for Implementation Research. Consolidated Framework for Implementation Research. 2019; https://cfirguide.org/. Accessed May 15, 2019.

104.Lincoln Y, Guba E. *Naturalistic inquiry.* Newburry Park, CA: Sage Publications; 1985.
105.King N. Template Analysis. In: Syman G, Cassell C, eds. *Qualitative Methods and Analysis in Organisational Research: A Practical Guide.* London: Sage; 1998.
106.Kilbourne AM, Neumann MS, et al. Implementing evidence-based interventions in health care: application of the replicating effective programs framework. *Implement Sci.* 2007;2:42.
107.Spoont MR, Murdoch M, et al. Treatment receipt by veterans after a PTSD diagnosis in PTSD, mental health, or general medical clinics. *Psychiatr Serv.* 2010;61:58-63.
