# Supplementary material for: Enhanced Weight Management Program for Veterans With Posttraumatic Stress Disorder: A Randomized Clinical Trial
Source: JAMA Netw Open. 2026 Mar 27;9(3):e261904. doi: 10.1001/jamanetworkopen.2026.1904 (PMC13032152; doi:10.1001/jamanetworkopen.2026.1904)
Supplement: Supplement 2. — eTable 1. Quality of weights taken during study visits eTable 2. Characteristics at baseline, stratified by missingness on primary outcome (N=174) eTable 3. Clinically meaningful change in weight and PTSD symptoms eTable 4. Satisfaction with MOVE! eTable 5. Satisfaction with MOVE!+UP eTable 6. Sensitivity analyses eTable 7. Weight visualization sensitivity analysis eTable 8. Body systems involved in adverse events through 12 months eTable 9. Number of participants in baseline and follow-up for exploratory measures eFigure 1. Sensitivity analyses eFigure 2. Weight visualization sensitivity analyses [file jamanetwopen-e261904-s002.pdf]

Supplemental Online Content

Hoerster KD, Sulayman N, Hunter-Merrill R, et al. Effectiveness of an enhanced weight management program for veterans with posttraumatic stress disorder: a randomized clinical trial. *JAMA Netw Open*. 2026;9(3):e261904. doi:10.1001/jamanetworkopen.2026.1904

eTable 1. Quality of weights taken during study visits

eTable 2. Characteristics at baseline, stratified by missingness on primary outcome (N=174)

eTable 3. Clinically meaningful change in weight and PTSD symptoms

eTable 4. Satisfaction with MOVE!

eTable 5. Satisfaction with MOVE!+UP

eTable 6. Sensitivity analyses

eTable 7. Weight visualization sensitivity analysis

eTable 8. Body systems involved in adverse events through 12 months

eTable 9. Number of participants in baseline and follow-up for exploratory measures

eFigure 1. Sensitivity analyses

eFigure 2. Weight visualization sensitivity analyses

This supplemental material has been provided by the authors to give readers additional information about their work.

© 2026 Hoerster KD et al. *JAMA Network Open*.

eTable 1. Quality of weights taken during study visits

**Weight Visualization Status**	**Eligibility/Baseline** N (%)	**6 Months** N (%)	**12 Months** N (%)
Visualized on study scale during study visit	109 (62.6%)	109 (62.6%)	107 (61.5%)
Assumed visualized on study scale during study visit (i.e., no notes, especially in early cohorts when assessors were instructed to make notes if deviated)	30 (17.2%)	5 (2.9%)	5 (2.9%)
Visualized, on study scale, not during study visit (e.g., text photo)	0 (0.0%)	2 (1.1%)	8 (4.6%)
Visualized, not on study scale, not during study visit (e.g., text photo)	0 (0.0%)	0 (0.0%)	1 (0.6%)
Visualized, not on study scale, during study visit	0 (0.0%)	0 (0.0%)	0 (0.0%)
Not visualized, on study scale, during study visit	34 (19.5%)	24 (13.8%)	17 (9.8%)
Not visualized, on study scale, not during study visit	1 (0.6%)	5 (2.9%)	3 (1.7%)
Not visualized, not on study scale, during study visit	0 (0.0%)	1 (0.6%)	0 (0.0%)
Not visualized, not on study scale, not during study visit	0 (0.0%)	1 (0.6%)	3 (1.7%)
No weight recorded	0 (0.0%)	27 (15.5%)	30 (17.2%)

eTable 2. Characteristics at baseline, stratified by missingness on primary outcome (N=174)

**Characteristic**	**Not Missing 6-Month Weight** (N = 146)	**Missing 6-Month Weight** (N = 28)
**Randomization assignment**		
Control (n=89)	72 (49%)	17 (61%)
Intervention (n=85)	74 (51%)	11 (39%)
**Age, Mean (SD)**	55 (13)	55 (13)
**Body Mass Index, Mean (SD)**	34.2 (5.4)	35.1 (5.8)
**Weight, Mean (SD)**	228 (45)	240 (46)
**Depression symptom severity, Mean (SD)**	13.9 (4.5)	13.3 (4.8)
**Insomnia Severity Index, Mean (SD)**	17.1 (5.5)	16.5 (6.4)
**PTSD symptom severity, Mean (SD)**	53 (10)	48 (11)
**Weight loss medications, 12 months prior to baseline, Mean (SD)**	2.2 (3.9)	1.8 (5.6)
**MOVE! visits, 12 months prior to baseline, Mean (SD)**	0.64 (3.03)	1.39 (4.37)
**Mental health treatment, 12 months prior to baseline, Mean (SD)**	12 (16)	9 (10)
**Psychiatric medications, 12 months prior to baseline, Mean (SD)**	2.63 (3.34)	3.29 (2.98)
**Eating habits, Mean (SD)**	1.60 (0.76)	1.70 (0.66)
**Binge eating, N (%)**	25 (17%)	5 (20%)
**Light, moderate and vigorous physical activity, Mean (SD)**	225 (95)	211 (82)
**moderate and vigorous physical activity, Mean (SD)**	69 (56)	51 (30)
**Social support for healthy eating, Mean (SD)**	0.86 (0.64)	0.82 (0.74)
**Discouragement for healthy eating, Mean (SD)**	0.63 (0.55)	0.52 (0.67)
**Social support for physical activity, Mean (SD)**	0.78 (0.65)	0.63 (0.60)
**Discouragement for physical activity, Mean (SD)**	0.54 (0.54)	0.55 (0.76)
**Weight bias internalization**	3.62 (1.13)	4.05 (0.97)
**Adaptive Late-Life Function and Disability, Mean (SD)**	34 (11)	37 (12)
**Gender**		

© 2026 Hoerster KD et al. *JAMA Network Open*.

Female	57 (39%)	4 (14%)
Male	89 (61%)	24 (86%)
**Race**		
American Indian or Alaskan Native	3 (2.1%)	0 (0%)
Black or African-American	25 (17%)	6 (21%)
Filipino	3 (2.1%)	0 (0%)
Korean	1 (0.7%)	0 (0%)
Multiple categories	22 (15%)	2 (7.1%)
Other Asian	1 (0.7%)	0 (0%)
Other Pacific Islander	2 (1.4%)	0 (0%)
Some Other Race	2 (1.4%)	0 (0%)
White	87 (60%)	20 (71%)
**Hispanic, Latino or Spanish origin**		
No	131 (90%)	26 (93%)
Yes, Cuban	1 (0.7%)	0 (0%)
Yes, Mexican, Mexican American, Chicano	6 (4.1%)	0 (0%)
Yes, Puerto Rican	1 (0.7%)	2 (7.1%)
Yes, another Hispanic, Latino or Spanish origin (e.g., Argentinian, Colombian, Dominican, Nicaraguan, Salvadoran, Spaniard)	7 (4.8%)	0 (0%)
**Educational Attainment**		
Some high school or less	1 (0.7%)	0 (0%)
General equivalency diploma (GED)	0 (0%)	1 (3.6%)
Completed high school	4 (2.7%)	0 (0%)
Some college or vocational training	45 (31%)	5 (18%)
Completed associates degree	22 (15%)	7 (25%)
Completed college	44 (30%)	6 (21.4%)
Completed a graduate degree	30 (21%)	7 (25%)
Missing	0 (0%)	2 (7.1%)
**Employment Status**		
Employed full-time	25 (17%)	10 (36%)
Employed part-time	7 (4.8%)	2 (7.1%)
Disabled/receiving disability	56 (38%)	8 (29%)
Full-time student	2 (1.4%)	0 (0%)
Multiple categories	28 (19%)	3 (11%)
Part-time student	1 (0.7%)	0 (0%)
Retired	21 (14%)	2 (7.1%)
Unemployed (fired from, laid off from, or quit job)	6 (4.1%)	1 (3.6%)
Missing	0 (0%)	2 (7.1%)
**Relationship Status**		
Married	83 (57%)	14 (50%)
Living with significant other	7 (4.8%)	3 (11%)
Divorced	27 (18%)	7 (25%)
Multiple categories	3 (2.1%)	0 (0%)
Never married	18 (12%)	1 (3.6%)
Separated	3 (2.1%)	0 (0%)
Widowed	5 (3.4%)	1 (3.6%)
Missing	0 (0%)	2 (7.1%)
**Annual Household Family Income**		
< $20,000	7 (4.8%)	1 (3.6%)
$20,000-$40,000	25 (17%)	3 (11%)
$40,001-60,000	29 (20%)	5 (18%)

© 2026 Hoerster KD et al. *JAMA Network Open*.

$60,001-$80,000	21 (14%)	5 (18%)
$80,001-$100,000	23 (16%)	7 (25%)
>$100,000	37 (25%)	5 (18%)
Missing	4 (2.7%)	2 (7.1%)
**Military Branch**		
Air Force	19 (13%)	1 (3.6%)
Army	67 (46%)	14 (50%)
Coast Guard	3 (2.1%)	0 (0%)
Marines	10 (6.8%)	6 (21%)
Multiple categories	8 (5.5%)	0 (0%)
Navy	39 (27%)	5 (18%)
Missing	0 (0%)	2 (7.1%)
**Service Connected**		
10	2 (1.4%)	2 (7.1%)
20	1 (0.7%)	0 (0%)
40	2 (1.4%)	0 (0%)
50	4 (2.7%)	1 (3.6%)
60	4 (2.7%)	1 (3.6%)
70	15 (10%)	4 (14%)
80	15 (10%)	3 (11%)
90	19 (13%)	2 (7.1%)
100	80 (55%)	12 (43%)
Missing	4 (2.7%)	3 (11%)

Note: In this table, we did not collapse categorical variable response options. If a participant selected a category, it is presented. Because some race and ethnicity categories were not selected by anyone, those categories are not presented.

© 2026 Hoerster KD et al. *JAMA Network Open*.

eTable 3. Clinically meaningful change in weight and PTSD symptoms

**Outcome**	MOVE!, N = 89	MOVE!+UP, N = 85
Lost ≥ 5% of baseline weight, 6 months, N (%)	25 (35%)	30 (41%)
Missing	17 (19%)	11 (13%)
Lost ≥ 5% of baseline weight, 12 months, N (%)	29 (43%)	30 (40%)
Missing	21 (24%)	10 (12%)
≥ 9-point reduction on the PTSD symptom measure, 6 months, N (%)	28 (39%)	33 (45%)
Missing	17 (19%)	11 (13%)
≥ 9-point reduction on the PTSD symptom measure, 12 months, N (%)	38 (57%)	31 (42%)
Missing	22 (25%)	11 (13%)

© 2026 Hoerster KD et al. *JAMA Network Open*.

eTable 4. Satisfaction with MOVE!

Satisfaction item. How useful was…. ? (range 1 (not at all useful) -5 (very useful)).	Mean (SD)
…the content on avoiding added sugars and processed foods?	4.4 (0.9)
… the content on calorie goal setting?	3.9 (1.2)
… the content on coping with mental health symptoms such as PTSD?	3.8 (1.2)
… the content on creating a healthy plate including portion size and eating fruits and vegetables?	4.5 (0.9)
… the content on grocery shopping cooking and restaurant food?	4.3 (0.9)
… the content on mindful eating and balancing hunger and fullness?	4.5 (0.9)
… the content on physical activity including information regarding strength training?	4.2 (1.0)
… the content on problem solving barriers?	4.3 (0.9)
… the content on relationship strategies like communication skills?	4.1 (0.9)
… the content on setting activity and healthy eating beverage goals including using SMART goal method?	4.4 (0.9)
… the content on strategies for meeting goals like using reminders and rewards?	4.3 (0.9)
… the content on strategies for sleeping well?	4.0 (1.0)
… the content on sustaining progress and handling slips and setbacks?	4.3 (1.0)
… the content on understanding nutrition facts and reading food labels and ingredients lists?	4.4 (1.0)
… the content on using values to guide activity and eating choices?	4.0 (1.0)
… was the delivery modality (video, phone, etc)?	4.3 (1.0)
… the mindful eating exercise?	4.3 (0.9)
… the overall manual?	4.2 (1.0)
… the session activity: “Checking in on activity and diet goals and progress”?	4.4 (1.0)
… the session activity: “Checking in on weight goals and progress”?	4.2 (1.2)
… the session activity: “Introducing new information and skills”?	4.4 (0.9)
… the session activity: “Review of the prior session”?	3.8 (1.2)

© 2026 Hoerster KD et al. *JAMA Network Open*.

Satisfaction item. How useful was…. ? (range 1 (not at all useful) -5 (very useful)).	Mean (SD)
… the session activity: "Setting goals for coming week"?	4.3 (1.0)
… the support and guidance from the facilitators?	4.5 (0.9)
… the weekly homework?	3.5 (1.4)
… the phone counseling calls or email messaging?	4.1 (1.2)
… the weekly physical activity and eating beverage log including feedback from facilitators?	4.0 (1.3)
How satisfied were you with the length of group sessions?[a]	2.7 (0.7)
How satisfied were you with the number of group sessions?[b]	3.0 (0.8)

[a]Anchors were 1 (too short) to 5 (too long). Because of these different anchors from the rest, these numbers were not included in the average overall satisfaction figure presented in the manuscript results section.

[b]Anchors were 1 (too few) to 5 (too many). Because of these different anchors from the rest, these numbers were not included in the average overall satisfaction figure presented in the manuscript results section.

© 2026 Hoerster KD et al. *JAMA Network Open*.

eTable 5. Satisfaction with MOVE!+UP

Satisfaction item (range 1-5; higher = better). How useful was….	Mean (SD)
… the content on avoiding added sugars and processed foods?	4.3 (0.9)
… the content on calorie goal setting?	4.2 (1.0)
… the content on coping with mental health symptoms such as PTSD?	4.0 (1.2)
… the content on creating a healthy plate, including portion size and eating fruits and vegetables?	4.5 (0.8)
… the content on grocery shopping cooking and restaurant food?	4.3 (0.9)
… the content on mindful eating and balancing hunger and fullness?	4.4 (0.9)
… the content on physical activity including information regarding strength training?	4.4 (0.9)
… the content on problem solving barriers?	4.3 (0.9)
… the content on relationship strategies like communication skills?	4.4 (0.9)
… the content on setting activity and healthy eating beverage goals, including using the SMART goal method?	4.6 (0.7)
… the content on strategies for meeting goals like using reminders and rewards?	4.2 (1.1)
… the content on strategies for sleeping well?	4.0 (1.0)
… the content on sustaining progress and handling slips and setbacks.	4.5 (0.9)
… the content on understanding nutrition facts and reading food labels and ingredients lists?	4.4 (0.9)
… the content on using values to guide activity and eating choices?	4.2 (1.0)
… the delivery modality (video, phone, etc)?	4.4 (1.2)
… the mindful eating exercise?	4.4 (0.9)
… the overall manual?	4.4 (0.8)
… the session activity: “Checking in on activity and diet goals and progress”?	4.4 (1.0)
… the session activity: “Checking in on weight goals and progress”?	4.4 (0.9)
… the session activity: “Introducing new information and skills .	4.4 (0.9)
… the session activity: “Review of the prior session”?	4.2 (1.2)

© 2026 Hoerster KD et al. *JAMA Network Open*.

Satisfaction item (range 1-5; higher = better). How useful was….	Mean (SD)
… the session activity: "Setting goals for coming week"?	4.5 (0.8)
… the support and guidance from the 2 dietician visits?	4.0 (1.2)
… the support and guidance from the other facilitator?	4.5 (0.9)
… the support and guidance from the peer support counselor?	4.3 (1.1)
… the walking activity?	4.3 (1.2)
… the weekly homework?	4.0 (1.0)
… the phone counseling calls or email messaging?	4.3 (1.1)
… the weekly physical activity and eating beverage log including feedback from facilitators?	4.1 (1.1)
How satisfied were you with the length of group sessions? [a]	3.0 (0.6)
How satisfied were you with the number of group sessions[b]	3.2 (0.9)

[a]Anchors were 1 (too short) to 5 (too long). Because of these different anchors from the rest, these numbers were not included in the average overall satisfaction figure presented in the manuscript results section.

[b]Anchors were 1 (too few) to 5 (too many). Because of these different anchors from the rest, these numbers were not included in the average overall satisfaction figure presented in the manuscript results section.

© 2026 Hoerster KD et al. *JAMA Network Open*.

eTable 6. Sensitivity Analyses

	Treatment Effect (95% CI)	P-value
Weight Change		
At 6 months, adjusting for total MOVE! visits and whether took ≥ 1 weight management medication[a] in 12 months prior to baseline, EHR	-1.37 (-5.81, 3.07)	0.54
At 12 months, adjusting for total MOVE! visits and whether took ≥ 1 weight management medication[a] in 12 months prior to baseline, EHR	-1.89 (-7.33, 3.55)	0.49
At 6 months, adjusting for weight management co-intervention[b]	-2.35 (-7.21, 2.51)	0.34
At 12 months, adjusting for weight management co-intervention[b]	-2.66 (-8.49, 3.17)	0.37
At 6 months, with additional variables included in multiple imputation	-1.69 (-6.16, 2.79)	0.46
PCL-5/PTSD Symptom Change		
At 6 months, adjusting for total number of mental health visits and whether took ≥ 1 medication for PTSD[c] in 12 months prior to baseline, per EHR	-0.57 (-4.08, 2.94)	0.75
At 12 months, adjusting for total number of mental health visits and whether took ≥ 1 medication for PTSD[c] in 12 months prior to baseline, per EHR	0.9 (-3.42, 5.22)	0.68

[a]Binary indicator of prescription weight loss medications in EHR (Dulaglutide. Empagliflozin, Fluvoxamine, Furosemide, Lamotrigine, Liraglutide Losartan, Phentermine, Semaglutide, Topiramate)

[b]Binary indicator of prescription weight loss medications from baseline to 6 and 12 months reported in EHR and/or self report; binary indicator of non-MOVE!+UP/MOVE! weight management care (e.g., any self-reported non-VA program, dietician visits).

[c]sertraline, paroxetine, fluoxetine, venlafaxine, prazosin, nefazodone, imipramine, or phenelzine

© 2026 Hoerster KD et al. *JAMA Network Open*.

eTable 7. Weight Visualization Sensitivity Analysis

**Sensitivity Parameter**	**Treatment Effect** (95% CI)	**P-value**
0.75	-2.68 (-17.47, 12.11)	0.72
0.80	-2.35 (-15.1, 10.4)	0.72
0.85	-2.04 (-12.52, 8.44)	0.70
0.90	-1.8 (-9.84, 6.25)	0.66
0.95	-1.64 (-7.33, 4.05)	0.57
1.00	-1.52 (-5.93, 2.89)	0.50
1.05	-1.5 (-6.07, 3.07)	0.52
1.10	-1.62 (-7.15, 3.91)	0.56
1.15	-1.87 (-9.23, 5.48)	0.62
1.20	-2.18 (-11.69, 7.33)	0.65
1.25	-2.5 (-14.12, 9.11)	0.67

Note: We examined how potential under-reporting in unvisualized follow-up weights could impact primary findings by assuming that unvisualized weights are equal to the visualized weight multiplied by a factor, wherein Baseline: $Unverified = Verified \times \varepsilon$ and 6-months: $Unverified = Verified \times \delta$. We specified a sequence of m values $\varepsilon_1, \varepsilon_1, \dots, \varepsilon_m$ for ε, and another sequence of n values $\delta_1, \delta_2, \dots, \delta_n$ for δ. For each ε_i and δ_j, we created a corresponding dataset where all unverified weights were replaced by "verified" versions. We then re-ran the primary analysis, generating a sequence of effect estimates $\Delta_1, \Delta_2, \dots, \Delta_{mxn}$, which were compared to the treatment effect estimate from the primary analysis. The sensitivity parameters for ε and δ ranged from 0.5 to 1.5 in increments of 0.05, allowing us to assess how extreme the bias in unvisualized weights would have to be before the primary results would be impacted.

© 2026 Hoerster KD et al. *JAMA Network Open*.

eTable 8. Body systems involved in adverse events through 12 months

Body System Involved	Serious Adverse Events (10 events total)		Non-serious Adverse Events (268 events total)	
	**MOVE!+UP** (5 events)	**MOVE!** (5[a] events)	**MOVE!+UP** (140 events)	**MOVE!** (128 events)
**Cardiovascular**	20%	75%	8.6%	6.3%
**Dermatological**	0%	0%	1.4%	3.9%
**Gastrointestinal**	40%	0%	6.4%	7.0%
**Hematological**	20%	0%	0.7%	0.4%
**Hepatobiliary**	0%	0%	0%	0%
**Metabolic**	20%	0%	2.9%	2.3%
**Musculoskeletal**	0%	0%	26.4%	34.4%
**Neurological**	0%	25%	10.7%	9.4%
**Psychological**	0%	0%	22.1%	14.1%
**Renal/Urologic**	20%	0%	5.0%	5.5%
**Respiratory**	0%	0%	13.6%	14.8%
**Other**	0%	25%	6.4%	4.7%

Note: Any one event may have involved multiple body systems, so percentages rather than Ns are presented and add to more than 100%. Multiple events could be recorded for individual participants. Percentages reflect the proportion of events accounted for by each body system.

Note: This relatively high amount of AEs was expected given the high level of chronic medical and mental health conditions in the population. Furthermore, in addition to the follow-up survey assessments, facilitators of both groups were asked to report any new or worsening medical conditions, and ER visits or hospitalizations, yielding frequent reports.

[a]One SAE death involving a MOVE! participant (of two deaths total) was unable to be assessed and rated for relatedness, unexpectedness, and body system due to not having any details regarding participant death.

© 2026 Hoerster KD et al. *JAMA Network Open*.

eTable 9. Number of participants in baseline and follow-up for exploratory measures (0=MOVE! and 1=MOVE!+UP)

Variable	N
depression_baseline_0	87
depression_baseline_1	83
depression_6mo_0	69
depression_6mo_1	72
depression_change_6mo_0	68
depression_change_6mo_1	71
isi_baseline_0	87
isi_baseline_1	82
isi_6mo_0	67
isi_6mo_1	71
isi_change_6mo_0	66
isi_change_6mo_1	70
Social support pa_baseline_0	88
Social support pa_baseline_1	83
Social support pa_6mo_0	68
Social support pa_6mo_1	72
Social support pa_change_6mo_0	68
Social support pa_change_6mo_1	71
Diet quality_baseline_0	88
Diet quality _baseline_1	83
Diet quality _6mo_0	68
Diet quality _6mo_1	72
Diet quality _change_6mo_0	68
Diet quality _change_6mo_1	71
Social support healthy eating_baseline_0	88
Social support healthy eating _baseline_1	84
Social support healthy eating _6mo_0	68
Social support healthy eating _6mo_1	72
Social support healthy eating _change_6mo_0	68
Social support healthy eating _change_6mo_1	72
Discourage healthy eating_baseline_0	88
Discourage healthy eating _baseline_1	83
Discourage healthy eating _6mo_0	68
Discourage healthy eating _6mo_1	72
Discourage healthy eating _change_6mo_0	68
Discourage healthy eating _change_6mo_1	71
Emotional overeating_baseline_0	88
Emotional overeating _baseline_1	83
Emotional overeating _6mo_0	67
Emotional overeating _6mo_1	71
Emotional overeating _change_6mo_0	67
Emotional overeating _change_6mo_1	70

© 2026 Hoerster KD et al. *JAMA Network Open*.

Discourage pa_baseline_0	88
Discourage pa_baseline_1	84
Discourage pa_6mo_0	67
Discourage pa_6mo_1	72
Discourage pa_change_6mo_0	67
Discourage pa_change_6mo_1	72
wbis_baseline_0	88
wbis_baseline_1	84
wbis_6mo_0	68
wbis_6mo_1	72
wbis_change_6mo_0	68
wbis_change_6mo_1	72
Eating habits_baseline_0	88
Eating habits _baseline_1	84
Eating habits _6mo_0	68
Eating habits _6mo_1	72
Eating habits _change_6mo_0	68
Eating habits _change_6mo_1	72
Night eating baseline_0	88
Night eating _baseline_1	84
Night eating _6mo_0	68
Night eating _6mo_1	70
Night eating _change_6mo_0	68
Night eating _change_6mo_1	70
average_lmvpa_bl_0	83
average_lmvpa_bl_1	79
average_lmvpa_6mo_0	54
average_lmvpa_6mo_1	61
average_lmvpa_diff_0	54
average_lmvpa_diff_1	59
average_mvpa_bl_0	83
average_mvpa_bl_1	79
average_mvpa_6mo_0	54
average_mvpa_6mo_1	61
average_mvpa_diff_0	54
average_mvpa_diff_1	59

© 2026 Hoerster KD et al. *JAMA Network Open*.

eFigure 1. Sensitivity analyses

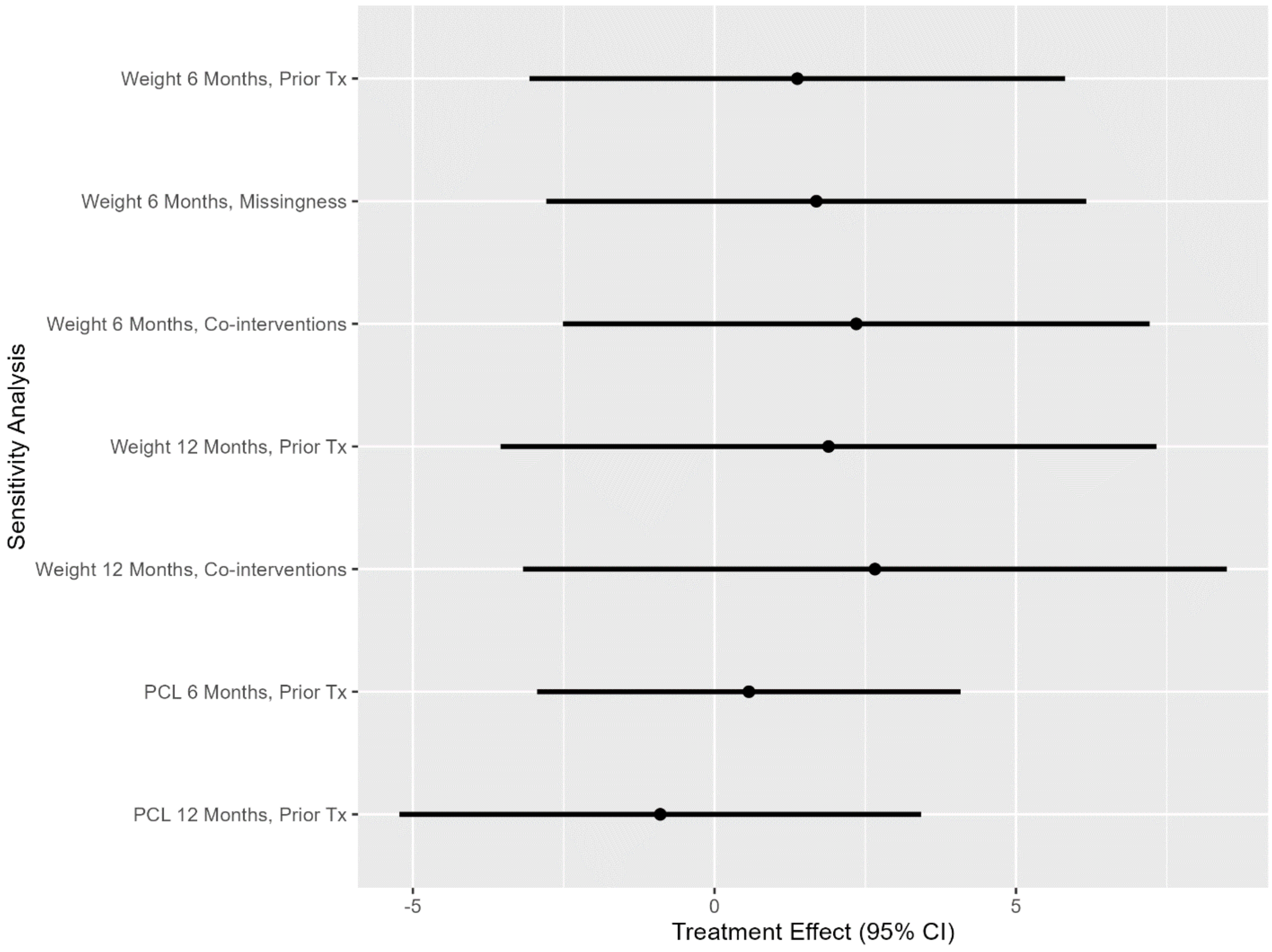

© 2026 Hoerster KD et al. *JAMA Network Open*.

eFigure 1. Sensitivity analyses

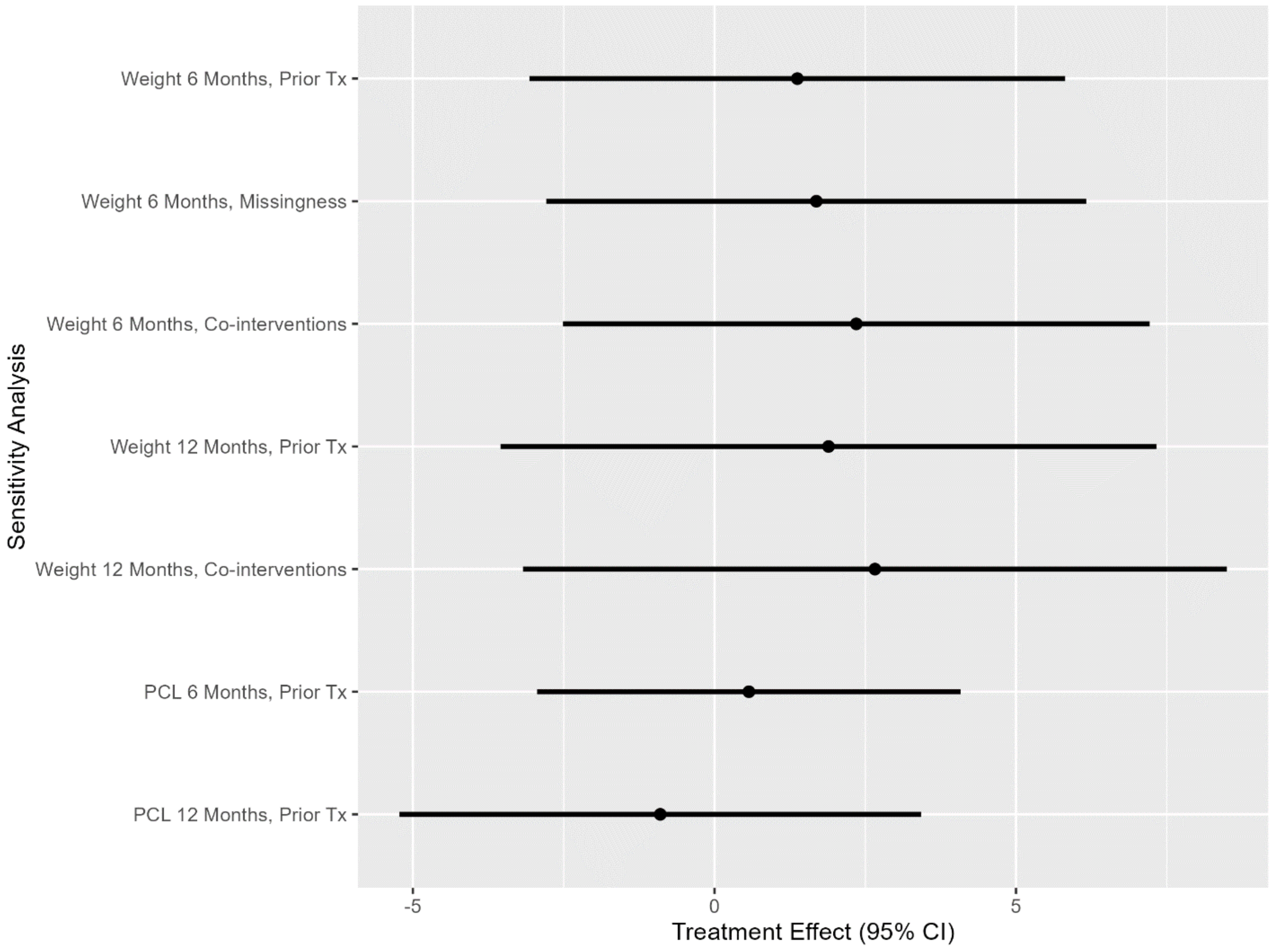

© 2026 Hoerster KD et al. *JAMA Network Open*.

eFigure 2. Weight visualization sensitivity analyses

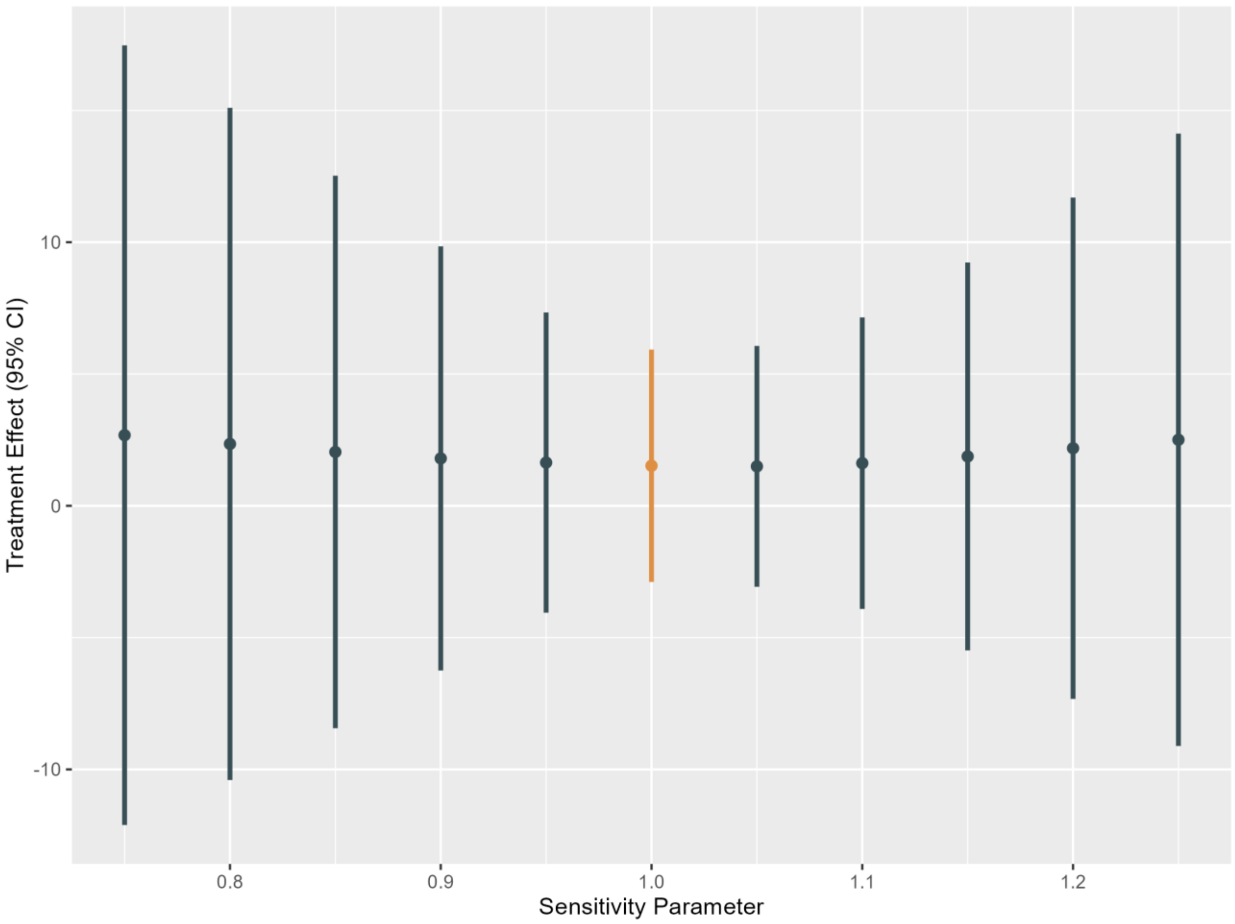

© 2026 Hoerster KD et al. *JAMA Network Open*.
