# Supplementary material for: Enhanced Weight Management Program for Veterans With Posttraumatic Stress Disorder: A Randomized Clinical Trial
Source: JAMA Netw Open. 2026 Mar 27;9(3):e261904. doi: 10.1001/jamanetworkopen.2026.1904 (PMC13032152; doi:10.1001/jamanetworkopen.2026.1904)
Supplement: Supplement 3. — Data Sharing Statement [file jamanetwopen-e261904-s003.pdf]

Data Sharing Statement

Hoerster. Enhanced Weight Management Program for Veterans With Posttraumatic Stress Disorder. *JAMA Netw Open*. Published March 25, 2026.
doi:10.1001/jamanetworkopen.2026.1904

Data
Additional Information: MOVE!+UP: Testing a Tailored Weight Management Program for Veterans With PTSD ClinicalTrials.gov ID: NCT04563741
Data available: Yes
Data types: Deidentified participant data, Data dictionary
How to access data: Requests for data should be made to the corresponding author. We will seek IRB approval to share deidentified data in these instances (which we anticipate would be provided quite swiftly).
When available: With publication

Supporting Documents
Document types: Statistical/analytic code, Informed consent form
How to access documents: Upon request to the corresponding author
When available: With publication

Additional Information
Who can access the data: These would be provided to anyone making a request to the corresponding author
Types of analyses: For use in replication analyses, meta-analysis, or other purpose (pending approval by our IRB).
Mechanisms of data availability: Our IRB will require use of a signed data access agreement
